# Recent research and progress of biodegradable zinc alloys and composites for biomedical applications: Biomechanical and biocorrosion perspectives

**DOI:** 10.1016/j.bioactmat.2020.09.013

**Published:** 2020-09-30

**Authors:** Humayun Kabir, Khurram Munir, Cuie Wen, Yuncang Li

**Affiliations:** School of Engineering, RMIT University, Melbourne, Victoria, 3001, Australia

**Keywords:** Biocorrosion, Biodegradable metals, Tissue engineering, Zinc-based alloys and composites

## Abstract

Biodegradable metals (BMs) gradually degrade *in vivo* by releasing corrosion products once exposed to the physiological environment in the body. Complete dissolution of biodegradable implants assists tissue healing, with no implant residues in the surrounding tissues. In recent years, three classes of BMs have been extensively investigated, including magnesium (Mg)-based, iron (Fe)-based, and zinc (Zn)-based BMs. Among these three BMs, Mg-based materials have undergone the most clinical trials. However, Mg-based BMs generally exhibit faster degradation rates, which may not match the healing periods for bone tissue, whereas Fe-based BMs exhibit slower and less complete *in vivo* degradation. Zn-based BMs are now considered a new class of BMs due to their intermediate degradation rates, which fall between those of Mg-based BMs and Fe-based BMs, thus requiring extensive research to validate their suitability for biomedical applications. In the present study, recent research and development on Zn-based BMs are reviewed in conjunction with discussion of their advantages and limitations in relation to existing BMs. The underlying roles of alloy composition, microstructure, and processing technique on the mechanical and corrosion properties of Zn-based BMs are also discussed.

## Highlights

• Zn alloys possess more suitable degradation rate than Fe, Mg, and their alloys.
• HE, HR, ECAP, and HPT are effective processes in improving mechanical properties.
• Nutrient elements Mg, Ca, Sr are also effective in boosting mechanical properties.
• Mechanically crucial alloying elements Cu, Fe, Mn are also good for bone health.
• Zr, Li, Ti, Ge, Al, Ag are vital elements in achieving good overall properties.

## Graphical abstract

**Figure:**
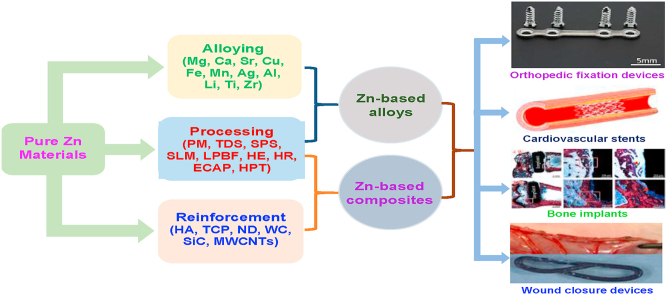

## 1 Introduction

Engineered or natural materials that are used directly to supplement the functions of living tissue are known as biomaterials and they have been utilized as implant materials for a long time in the field of medical science [[1], [2], [3], [4]]. Conventional non-degradable metallic biomaterials, such as stainless steels (SS), cobalt–chromium (Co–Cr) alloys, and titanium (Ti) and some of its alloys, are generally used as permanent or temporary implants to restore function by providing support to hard tissues. These metallic biomaterials have been extensively used for diverse biomedical applications, including joint replacement, fracture fixation, cardiovascular stents, and remodeling of bone, because of their high mechanical strength and corrosion resistance [[5], [6], [7], [8], [9], [10], [11], [12], [13], [14]]. However, these materials contain various alloying elements such as aluminum (Al), vanadium (V), chromium (Cr), and nickel (Ni) which adversely affect their biocompatibility for tissue-engineering applications. Ion release of these elements from metallic implants due to corrosion or excessive wear triggers inflammation and potentially several complex allergic reactions, which cause destruction of host tissues followed by loosening of the metallic implant [[15], [16], [17]]. Moreover, the mismatch between the elastic modulus of natural bone (3–30 GPa) and those of these metallic implant materials (190–200 GPa for SS, 210–240 GPa for Co–Cr alloys, and 90–110 GPa for Ti alloys) leads to stress shielding of the surrounding bone that causes bone resorption and subsequent implant loosening. Such implant failures often require additional complex revision surgeries to remove or replace them [[18], [19], [20]].

Therefore, biodegradable metals (BMs) have been developed to overcome these problems and to prevent the need for revision surgery generally required to remove metallic implants upon restoration of tissue function [[21], [22], [23], [24], [25]]. Compared to non-biodegradable metals, BMs can provide the necessary support to host tissues undergoing a regeneration process and they degrade naturally in the physiological environment and dissolve entirely after sufficient tissue healing, while their by-products can be metabolized by the body as they are usually non-toxic [[26], [27], [28], [29]]. Moreover, BMs may contain trace elements indispensable in the body for performing a variety of biological functions [[30], [31], [32], [33]]. Several key advantages and limitations of existing non-biodegradable metals and BMs are summarized in Table 1. Table 1 also includes various applications of both non-biodegradable metals and BMs., e.g., structural implants, such as stents, braces, rods, heart valves, bones, pins, hip prosthesis, eye, ear, skull implants and knee replacement implants.

**Table 1:** Major advantages and limitations of various metallic biomaterials [[34], [35], [36], [37], [38], [39], [40], [41], [42]].

Classification	Materials	Benefits	Limitations	Applications
**Non-biodegradable metallic materials**	316L SS	High tensile strength, toughness, and acceptable biocompatibility.	Low wear and corrosion resistance, high elastic modulus, localized corrosion with pitting, crevices and stress corrosion cracking.	Acetabular cup, bone plates, bone screws, pins, rods, hip nails, wires, total hip replacements, etc.
	Co–Cr alloys	Good corrosion, fatigue and wear resistance, and high mechanical strength.	High elastic modulus, toxicity due to release of Co, Cr, and Ni ions.	Short term implants, bone plates and wires, orthodontic wire, femoral stems, total joint replacements, etc.
	Ti alloys	Very good biocompatibility, tensile strength and corrosion resistance, lightweight, and MRI compatible.	Expensive, poor wear resistance and fatigue strength.	Joint replacements, dental implants, cardiovascular implants, prosthetic heart valves, fracture fixation plates, fasteners, nails, rods, screws, and wires.
**Biodegradable metallic materials**	Mg-based alloys	Good biocompatibility, ability to stimulate new bone formation, biodegradable in a physiological environment, density and elastic modulus close to those of natural bone, and MRI compatible.	High degradation rate, unwanted pH increase in surrounding tissues, inadequate mechanical strength for load-bearing implants, premature loss of mechanical integrity before sufficient bone tissue healing, high H_2_ gas evolution; Degradation via Mg + 2H_2_O → Mg(OH)_2_ + H_2_.	Bone screws, bone plates, bone pins, cardiovascular stents etc.
	Fe-based alloys	High tensile strength and formability, fair biocompatibility, MRI compatible (austenitic phase), and no H_2_ gas production during degradation.	Very low degradation rate, high elastic modulus; Degradation via 2Fe + 2H_2_O + O_2_ → 2Fe(OH)_2_.	Temporary cardiovascular and orthopedic implants
	Zn-based alloys	Intermediate corrosion rate (falling between corrosion rates of Mg and Fe), fair biocompatibility, no H_2_ gas evaluation and non-toxic corrosion products, good processability, low melting point, and less reactivity in molten state.	Low mechanical strength, age hardening; Degradation via 2Zn + 2H_2_O + O_2_ → 2Zn(OH)_2_.	Stents (cardiovascular and coronary stents), orthopedic fixation (sutures, screw, pins and plates).

Materials based on iron (Fe), zinc (Zn), and magnesium (Mg) have been widely investigated as potential BMs for orthopedic applications [15,22,31,37,[43], [44], [45], [46], [47], [48], [49], [50]]. The microstructure of Zn alloys mainly contains a matrix phase (α-Zn) and second phases called intermetallic phases, which are generally hard and brittle. The mechanical properties of Zn alloys are significantly influenced by those intermetallic phases, and their volume fractions, sizes, and distribution in Zn matrix; and these microstructural characteristics are dependent on the fabrication and processing methods [37]. The microstructure of Mg alloys typically consists of primary α-Mg matrix and multiple second phases mainly distributed along grain boundary. These secondary phases are precipitated from Mg matrix along grain boundaries and can promote the strength of Mg alloys by dispersion strengthening. The concentration and distribution of secondary phases affect the corrosion behavior of Mg alloys. A fine and continuous distribution of secondary phases significantly improves the corrosion performance of Mg alloys [14].

Compared to Zn alloys, Mg alloys exhibit higher strength, ductility and formability. Mg alloys also possess an elastic modulus (~45 MPa) approximating that of natural bone (10–30 GPa). However, Zn exhibits a lower corrosion rate than that of Mg because their electrode potential is −2.37 V and −0.76 V, respectively [14,51]. This makes Zn and its alloys a very hot research topic in the area of biodegradable metal alloys.

However, Mg and its alloys exhibit low density (1.7–2.0 g/cm^3^), low elastic modulus (40–45 GPa), and high specific strength relative to other BMs [52,53]. Their bone-mimicking elastic modulus is advantageous in minimizing the stress-shielding effect. Also, Mg is a vital nutrient and is responsible for many biological roles in the body [54,55]. Therefore, Mg-based BMs have been used in numerous biomedical applications, e.g., fracture fixation devices (bone screws, pins, plates), bioresorbable scaffolds for tissue engineering, and cardiovascular stents [23,24,32,[56], [57], [58]]. However, Mg-based BMs generally degrade rapidly in the body (within 2–3 months following implantation) and their rapid degradation is associated with the evolution of excessive hydrogen gas (H_2_) at the interface between the surrounding tissue and the implant [19,[59], [60], [61], [62]].

Fe-based BMs possess outstanding mechanical properties (such as tensile yield strength (σ_TYS_) = 250–950 MPa, ultimate tensile strength (σ_UTS_) = 300–1550 MPa, elongation (ε) = 2.0–19.5%, and micro-hardness (H) = 85–437 H V), superb formability, and good biocompatibility [63,64]. Fe is also a vital nutrient in the body which is responsible for numerous enzymatic functions. However, Fe and Fe-based BMs usually degrade very slowly (over more than 2–3 years), with degradation rates significantly below clinical requirements, and thus may generate analogous complications (such as fragment embolization) as noted with other non-biodegradable implants [[65], [66], [67], [68]]. Also, their corrosion by-products are not excreted from the body at a satisfactory rate and are retained in the surrounding tissues as well as biological matrices for long periods [69]. Moreover, Fe-based BMs exhibit a higher elastic modulus (200–210 GPa) compared to those of Mg-based BMs (40–45 GPa) and Zn-based BMs (90–100 GPa) [70,71].

**Fig. 1:**
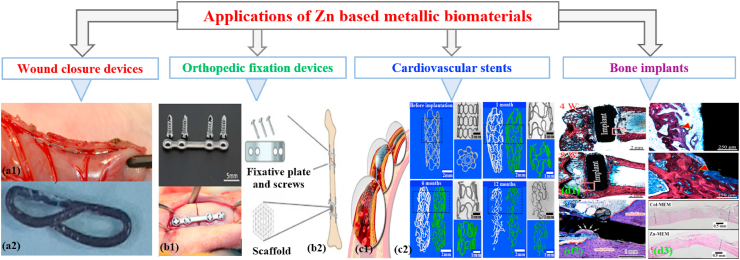
Potential biomedical applications of Zn-based materials: (a1) staple line made from Zn alloy [86], (a2) macroscopic appearance of Zn alloy staples [86], (b1) Zn alloy plate and screws, and fixed mandibular bone fractures immediately after surgery [87], (b2) Zn-based fixative plates, screws, and porous scaffolds providing temporary mechanical support for bone tissue regeneration [88], (c1) schematic illustration of stent implantation into a coronary vessel [89], (c2) selected 2D and 3D micro-CT images of Zn stents after different implantation time [90], (d1) histological characterization of hard tissue sections at implant sites for Zn-5HA composite at week 4 and 8, the red triangle indicates newly formed bone [91], (d2) histological observation of different parts of the implant in the bone environment at 6 months (blue arrows indicate the bones surrounding the implant in the medullary cavity, and white arrows mark the locally corroded site) [92], (d3) histological images showing the maturation of the newly formed bone in the Zn-MEM compared with the still un-mineralized bone matrix in the Col-MEM group [93].

However, Zn-based BMs exhibit intermediate degradation rates as compared to other BMs. Hence, Zn, its alloys and composites are emerging as a new class of BMs and are considered promising alternatives to Mg-based and Fe-based BMs for biomedical applications, particularly orthopedic regeneration, and cardiovascular therapy [68,[72], [73], [74], [75], [76], [77]]. This is mainly because Zn-based BMs exhibit more suitable degradation rates than those of Mg-based and Fe-based BMs, and their degradation products are fully bioresorbable without evolving excessive H_2_ gas [[78], [79], [80]]. In addition to the appropriate degradation rate, the importance of Zn as nutrient in the human body has been reported in several studies. For example, Zn is known as the “calcium” of the twenty-first century because of its many important biological roles in the body including nucleic acid metabolism, stimulation of new bone formation, signal transduction, preserving bone mass, apoptosis regulation, and gene expression [[81], [82], [83]]. Zn not only suppresses bone-tissue loss and inflammatory-related diseases, but also plays a significant role in cartilage matrix metabolism (SOX9) and cartilage II gene expression [84]. Consequently, compared to Mg-based and Fe-based BMs, Zn-based BMs have emerged as the next generation of BMs for bone-tissue engineering. Fig. 1 shows an illustration of various *in vivo* studies using Zn-based materials for potential clinical applications [[85], [86], [87], [88], [89], [90], [91], [92], [93]].

Nevertheless, pure Zn exhibits inadequate mechanical properties, such as poor σ_UTS_ (20–30 MPa), ε (0.25%), and H (37 HV) [94], and so it cannot be used for most clinical applications such as stents and orthopedic fixation devices. In addition, the relatively low fatigue strength and creep resistance, low-temperature recrystallization, and high susceptibility to natural aging of Zn and Zn alloys may lead to failure of medical devices during storage at room temperature (RT) and during utilization in the body [80].

A summary of some physical and mechanical properties of existing non-biodegradable and biodegradable metallic biomaterials, along with features of natural bone tissues, is given in Table 2. It can be seen that the pure Zn shows the lowest σ_UTS_, σ_TYS_, and ε among all the metallic biomaterials. Therefore, the development of Zn alloys with higher σ_UTS_, σ_TYS_, and ε is one of the main challenges to its suitability as a candidate material for biomedical applications. The mechanical properties of Zn alloys can be enhanced by tailoring their microstructures via alloying and special fabrication techniques followed by several post treatment [37,[101], [102], [103]]. In the past few years, several studies have reported advancements in the development of Zn-based BMs [36,37,68,79,80,88,89]. Zn-based materials can be used for a variety of biomedical applications, such as wound closure devices (biodegradable staples, surgical tacks, plugs, microclips, and rivets), orthopedic fixation devices (fixative plates, screws, and porous scaffolds), cardiovascular stents, and bone implants. In this study, the chemical compositions, various fabrication techniques, and a variety of post-thermomechanical processing routes for manufacturing Zn-based alloys and composites are analyzed in conjunction with analysis of their microstructures, mechanical properties, *in vitro* and *in vivo* degradation behaviors, and biocompatibility.

**Table 2:** Comparison of physical and mechanical properties of bone tissues along with existing non-biodegradable and biodegradable metallic materials.

**Tissue/Material**	**ρ (g/cm**^**3**^**)**	**σ**_**UTS**_**(MPa)**	**σ**_**TYS**_**(MPa)**	**E (GPa)**	**ε (%)**	Ref.
**Cortical bone**	1.8–2.0	35–283	105–114	5–23	1.07–2.1	[19,95]
**Trabecular bone**	1.0–1.4	1.5–38	1–12	0.01–1.6	2.20–8.5	[19,96]
**316L stainless steel**	8.0	450–650	200–300	190	30–40	[95]
**Co–Cr alloy (ASTMF90)**	9.2	860	310	210	20	[97]
**Ti–6Al–4V (Annealed)**	4.4	895–1025	825–869	110–114	6–10	[98]
**Pure Mg**	1.7–2.0	90–190	65–100	41–45	2–10	[52]
**Pure Fe (99.8%)**	7.8	180–210	120–150	211.4	40	[64,99]
**Pure Zn (As cast and hot-rolled)**	7.14	18–140	10–110	1.2–2.1	0.3–36	[79,100]

ρ: density, σ_UTS_: ultimate tensile strength, σ_TYS_: tensile yield strength, E: Young's modulus, ε: elongation.

## 2 Fabrication and post-thermomechanical processing of Zn-based biodegradable metals

### 2.1 Fabrication of Zn-based BMs

The fabrication processes for Zn-based alloys include casting, transient directional solidification, conventional powder metallurgy (PM), additive manufacturing (AM), and spark plasma sintering.

#### 2.1.1 Casting

Mass production of Zn-based alloys is performed using casting because this provides easy customization of alloy composition. Casting process can also produce complex shapes by designing complex internal cavities in molds, but the as-cast products contain defects in several forms of pores, shrinkages, pinholes, and cracks, and casting dimensional accuracy is low in comparison to machining components. However, the processing of alloys via casting involves melting the metal components of the alloy, then pouring the molten metal into a mold, and finally solidification. The melting is performed inside a furnace (typically, a resistance or induction furnace) at a temperature generally between 450 and 750 °C based on alloy composition, in a vacuum or a protective atmosphere of gases such as argon (Ar), CO_2_, or SF_6_. A controlled environment during casting is required in order to avoid oxidation reactions, and to control gas dissolution so as to minimize porosity. The molten metal is poured into a suitable steel or graphite mold with the desired shape of ingot for solidification [[104], [105], [106]].

The casting process can be classified into three subgroups, namely, squeeze casting (or die casting), gravity casting, and sand casting. Squeeze casting is the most common processing technique to fabricate Zn alloys. In this process, molten metal is forced into the mold cavity under elevated pressure [107,108]. Gravity casting involves the direct pouring of liquefied metals from the crucible into the mold [109]. Sand casting employs disposable sand molds to create metal parts with complex geometries. Some essential properties of as-cast Zn-based alloys along with their processing parameters are summarized in Table 3.

**Table 3:** Effects of fabrication techniques, alloying elements, and processing parameters on different properties of Zn alloys.

Fabrication method	**Composition (wt. %)**	Processing parameters	Key microstructures, mechanical and corrosion properties	Ref.
**Casting**	Zn-xMg (x = 0.5, 1.0, 2.0, 5.0)	Melting at 530 °C under air atmosphere in a gas fired pit furnace.	As-cast Zn–Mg alloys with ≤2.0% Mg contained mainly α-Zn and Mg_2_Zn_11_. One additional phase (MgZn_2_) was also observed in Zn–5Mg and Zn–7Mg alloys. The values of micro-hardness (H) and σ_UTS_ increased with increasing content of Mg due to the formation of Mg_2_Zn_11_ phase, but the values of ε did not follow any trend with Mg content. A maximum ε of 4.8% was observed in Zn-0.5 Mg.	[134]
	Zn-1.5 Mg, Zn-1.5Mg-0.1Ca, Zn-1.5Mg-0.1Sr	Melting at 470–520 °C under protection of CO_2_ gas and stirred for 30 min, and then was cast into a steel mold at about 430 °C.	As-cast ternary alloys were composed of matrix Zn and a mixture of precipitated phases of Mg_2_Zn_11_ and CaZn_13_ for Zn-1.5Mg-0.1Ca, Mg_2_Zn_11_ and SrZn_13_ for Zn-1.5Mg-0.1Sr, respectively. The addition of Ca/Sr refined the grain sizes. The ternary alloys exhibited much higher σ_TYS_, σ_UTS_ and ε than those of Zn-1.5 Mg. The corrosion rates (CRs) of the ternary alloys were slightly increased due to galvanic corrosion reaction.	[135]
	Zn-0.5Al-xMg (x = 0.1, 0.3, 0.5)	Melting at 580 °C in an electrical resistance furnace under protection of Ar + CO_2_ gas and stirred for 20 min.	As-cast Zn-0.5Al-xMg alloys were composed of α-Zn and Mg_2_(Zn, Al)_11_ with a fine lamellar structure, while Zn-0.5Al contained only α-Zn. The H, σ_UTS_ and ε were increased with increasing Mg content in ternary alloys from 71 to 94 Hv, 79–102 MPa and 1.5–2.1%, respectively. Conversely, corrosion current density, I_corr_ and CR were decreased from 20.4 to 9.5 μA/cm^2^ and 0.147–0.80 mm/y, respectively.	[136]
**TDS**	Zn-2.2 Mg, Zn-3.15 Mg	Melting at 550 °C in an electrical resistance furnace under protection of Ar gas and stirred for 20 min, and casting chamber of the DS apparatus was subjected to a forced Ar gas flow, position of thermocouple, *P* = 4–30 mm, solidification cooling rate, *T*˙ = 0.9–13.8 K/s, solidification growth rate (V_L_) = 0.28–1.51 mm/s.	Zn-2.2 Mg contained α-Zn exhibiting an equiaxed dendritic morphology with interdendritic regions constituted by a competitive mixture of stable (α-Zn + Zn_11_Mg_2_) and metastable (α-Zn + Zn_2_Mg) eutectic mixtures of lamellar morphology. Zn-3.15 Mg contained eutectic phase along with the entire DS casting with bimodal lamellar morphology. With decreasing cooling rate from 13.8 to 3.9 K/s, σ_UTS_ and ε of Zn-2.2 Mg reduced from 217 to 194 MPa and 4.9 to 3.1%, respectively, while Zn-3.15 Mg showed σ_UTS_ decreased from 259 to 193 MPa and ε decreased 4.9 to 2.1%. Also, Zn-3.15 Mg exhibited better corrosion resistance.	[137]
**PM**	Zn–20Mg	Ball type, BT = Stainless steel, Ball diameter, BD = 10 mm, ball to powder ratio, BPR = 10:1, Milling speed, MS = 250 rpm, Milling duration, MD = 8 h, Pressing pressure, PP = 30 MPa, Sintering temperature, ST = 410 °C, Sintering duration, SD = 30 min.	Zn–20Mg showed a density of 5.01 g/cm^3^ and a hardness of 82 Hv, respectively.	[114]
	Zn–1Mg, Zn–25Mg (wt.%)	MD = 8 h, ST = 430 °C, SD = 4 h.	Zn–1Mg alloy showed zinc matrix with a significant fine grain size (7.3 μm) and some MgZn_2_ intermetallic phase. Along with those phases, an additional Mg_2_Zn_11_ phase was observed in Zn–25Mg alloy. With increasing Mg content from 1 to 25 wt%, compressive yield strength (σ_CYS_), H, E, I_corr_ and CR was increased from 245 to 403 MPa, 81 to 140 Hv, 80–86 GPa, 7.24–12.99 μA/cm^2^ and 0.208–0.374 mm/y, respectively.	[138]
	Zn–0Mn, Zn–4Mn, Zn–24Mn	BT = Stainless steel, BPR = 20:1, MS = 250 rpm, MD = 8 h, PCA = Toluene, PP = 300 MPa, ST = 250–415 °C, SD = 1 h	Zn–4Mn showed higher σ_CYS_, ε and H than those of pure Zn and Zn–24Mn. Zn–4Mn and Zn–24Mn contained second phases that resulted in decreased CR in Zn–24Mn. The CR of Zn, Zn–4Mn and Zn–24Mn was 2.71, 0.72 and 0.02 mm/y, respectively.	[139]
**SPS**	Porous Zn	Sintered at 300 °C for 10 min under 5 MPa at a heating rate 100 °C/min in an argon atmosphere + extrusion at 300 °C with an extrusion ratio of 10 and at an extrusion rate of 0.3 mm/s.	Materials prepared from coarse Zn powders (size ~ 600–850 μm) are designated as CP, while that from fine powders (size ~ 40–100 μm) are as FP. The H, σ_CYS_, and E for CP and FP were 18.7 and 14.4 Hv, 43 and 31 MPa and 1.3 and 1.2 GPa, respectively, which are close to those values of trabecular bone (σ_CYS_ = 1–12 MPa and E = 0.1–0.4 GPa). The CR of porous Zn processed from CP and FP were 0.61 and 0.75 mm/y, respectively.	[100]
**SLM**	Pure Zn	Spot diameter, *d* = 75 μm, Defocus distance, *h* = 0 mm, Layer thickness, *D*s = 30 μm, Hatch spacing, *H*s = 70 μm, Laser power, *P* = 40–120 W, Scanning speed, *V* = 200–1000 mm/s, Volume energy, *E*_v_ = 20–200 J/mm^3^.	High density over 99.50% was obtained with *H*s = 70 μm, *D*s = 30 μm and *E*_v_ from 60 to 135 J/mm^3^. Deficient laser energy caused irregular cavities due to lack of fusion, while excessive laser energy resulted in circular pores due to gas entrapment. With high density, the mean values of H, σ_TYS_, σ_UTS_, E and ε were 42 HV, 114 MPa, 134 MPa, 23 GPa and 10.1%, respectively.	[140]
	Zn-xAg (x = 0, 2, 4, 6, 8)	*d* = 150 μm, *D*s = 100 μm, *P* = 70 W, *V* = 12 mms^−1^, Protective atmosphere = Ar gas.	The Zn–Ag alloys were significantly refined by alloying with Ag. Zn–6%Ag showed the smallest grain size of 25 μm, with a σ_CYS_ of 293 MPa and an H of 80 H V, 50% and 116% higher than those of pure Zn. The CR of alloys was increased compared to pure Zn owing to the formation of galvanic micro-cells between Zn matrix and AgZn_3_ phase.	[141]
	Zn-xMg (x = 0, 1, 2, 3, 4)	*d* = 150 μm, *D*s = 100 μm, *H*s = 80 μm, *P* = 200 W, *V* = 200 mms^−1^, Protective atmosphere = Ar gas.	Zn–Mg alloys contained fine equiaxed α-Zn grains with homogeneously precipitated Mg_2_Zn_11_ along grain boundaries, and the grains size of α-Zn was decreased from 104 to 5 μm with increasing Mg. The σ_UTS_ and ε of Zn–3Mg were increased by 361% and 423%, while the CR decreased from 0.18 to 0.10 mm/y, respectively.	[142]
	Zn–2Al	*d* = 150 μm, *D*s = 50 μm, *H*s = 70 μm, *P* = 60–160 W, *V* = 200–500 mm/s, *E*_v_ = 76–133 J/mm^3^, Protective atmosphere = Ar gas.	A low *E*_*v*_ caused formation of pores in Zn–2Al part, while a high *E*_*v*_ caused gasification of powder and failure of LPBF. A densification rate of 98.3% was achieved using a proper *E*_*v*_ (114.28 J/mm^3^). Zn–2Al part obtained at *E*_*v*_ of 114.28 J/mm^3^ exhibited an optimal H (64.5 Hv) and σ_UTS_ (192 MPa), and a CR of 0.14 mm/y.	[143]

TDS: transient directional solidification; PM: Powder metallurgy; SPS: Spark plasma sintering; SLM: selective laser melting.

#### 2.1.2 Transient directional solidification

The transient directional solidification (TDS) is the modified form of casting and can produce casting products without voids and internal cavities. It provides advantages to deal with the large variation of growth rate and cooling rate, which may allow a variety of microstructures and morphologies. The TDS has been developed to tailor the plate-like cellular microstructures of pure Zn and Zn-based alloys into dendritic-like equiaxed grains [110,111]. This technique is an alternative to traditional casting, where a special water-cooled apparatus is utilized to assist directional solidification (DS) and the process is carried out by controlling several conditions of heat flow. Moreover, a theoretical/experimental or combined approach is employed to calculate various solidification parameters (such as tip growth rate, cooling rate) that affect the microstructure of the resultant Zn alloys [112,113]. Zn–Mg alloys fabricated by TDS and their microstructures and mechanical properties are summarized in Table 3.

#### 2.1.3 Conventional powder metallurgy

A variety of metal and non-metal powders can be processed via powder metallurgy (PM) and excellent surface finish can be easily obtained. However, metal powders are prone to oxidation during processing which requires inert handling and processing of the metal powders. This processing technique generally consists of three steps: (i) milling or blending of different metal powders at different rotations per minute and intervals in the presence of a processing control agent; (ii) green compaction of metal powders; and (iii) sintering to attain semi-dense or fully dense solid materials. Sintering is performed under a controlled environment at a temperature 0.6–0.8 times the melting point of the metal powders. This process can also be used for the fabrication of biodegradable Zn-based alloys [114,115]. The maximum density of ~95% was achieved via hot pressing (HP), a process that simultaneously applies compression and sintering of the part [116,117]. The effects of alloying elements, fabrication techniques, and post-thermomechanical processes on the microstructure and mechanical properties of various Zn-based alloys are tabulated in Table 5.

**Table 4:** Effect of alloying elements and processing parameters on the different properties of Zn alloys after thermomechanical processing.

Thermomechanical processing	**Composition (wt.%)**	Processing parameters	Key microstructures, mechanical and corrosion properties	Ref.
**HE (Hot extrusion)**	Zn-xAg (x = 0, 2.5, 5.0, 7.0)	Melting at650 °C + homogenization at 410 °C for 12 h + HE at 250 °C with an extrusion ratio (ER) of 14: 1. Air cooling to room temperature (RT).	HE produced significant grain refinement (GR) of Zn–Ag alloys. The grain size (GS) decreased with increasing Ag content, with a remarkably fine and equiaxed microstructure and a mean grain size of about 1.5 μm for Zn-7.0Ag. Also, increasing Ag content monotonically improved σ_UTS_ from 203 to 287 MPa due to GR and a higher volume fraction (VF) of fine AgZn_3_ particles. The alloys showed slightly faster CRs compared to pure Zn.	[102]
	Zn-xCu (x = 1, 2, 3, and 4)	Melting at 650 °C + homogenization at 360 °C for 8 h followed by water quenching (WQ) + HE at 280 °C with an ER of 9: 1. Air cooling to RT.	The σ_TYS_, σ_UTS_ and ε of Zn-xCu alloys increased dramatically with increasing Cu content. Notably, the ε of Zn–4Cu reached 50.6%, which is beneficial for processing of micro-tubes for stent fabrication. The CRs of the alloys in SBF was low, varies from 22.1 to 33.0 μm/y.	[162]
	Zn–3Cu-xMg (x = 0, 0.1, 0.5 and 1.0)	Melting at 650–680 °C + homogenization at 360 °C for 8 h followed by WQ + then HE at 280 °C with an ER of 9: 1, Air cooling to RT.	The VF of Mg_2_Zn_11_ phase increased gradually with increasing Mg concentration. σ_TYS_ was improved from 213.7 to 426.7 MPa, while ε decreased from 47.1 to 0.9%. The CR increased from 11.4 to 43.2 μm/y.	[163]
	Pure Zn, Zn-0.8 Mg, Zn-1.6 Mg	Melting at 550–600 °C under air atmosphere + homogenization at 525 °C for 8 h followed by air cooling to RT + HE at 300 °C with an ER of 10: 1. Air cooling to RT.	The Zn–Mg alloys contained recrystallized Zn grains of 12 μm in size, and fine Mg_2_Zn_11_ particles arranged parallel to the extrusion direction. σ_CYS_, σ_TYS_, σ_UTS_ and H increased with increasing Mg content. Zn-0.8 Mg showed the best combination of mechanical properties (σ_TYS_ = 203 MPa, σ_UTS_ = 301 MPa and ε = 15%).	[164]
	Pure Zn, Zn-0.02Mg-0.02Cu	Melting at suitable temperature with Ar gas protection + HE at 180 °C with an ER of 16: 1. Air cooling to RT.	Compared with pure Zn, the Zn alloy showed higher mechanical properties (σ_TYS_ = 216 MPa, σ_UTS_ = 262 MPa, and H = 74 Hv).	[165]
	Pure Zn, Zn–1Mg-xZr (x = 0.1, 0.25, 0.4)	Melting at suitable temperature with Ar gas protection + homogenization at 343 °C for 36 h followed by WQ + HE at 250 °C with an ER of 16.7: 1. Air cooling to RT.	The HE seriously deformed the primary Zn-rich crystals and broke the Mg_2_Zn_11_ and Zn_22_Zr intermetallic compounds into small particles. Adding Mg and Zr to pure Zn significantly improved H (37–95 Hv), σ_TYS_ (61–248 MPa), σ_UTS_ (98–316 MPa) and σ_CYS_ (131–301 MPa). The addition of Zr to binary Zn–1Mg alloy slightly improved H, σ_UTS_, σ_TYS_ and σ_TYS_, and significantly improved ε from 0.8% to 4.7%.	[166]
**HR (Hot Rolling)**	Pure Zn, Zn–1Mg, Zn–1Ca, Zn–1Sr	Melting at 630 °C in mixed gas atmosphere (SF_6_ + CO_2_) for 0.5 h followed by air cooling to RT + rolling at 250 °C with total 81% reduction in thickness.	Hot rolling significantly increased the σ_TYS_, σ_UTS_ and ε of as-cast pure Zn, Zn–1Mg, Zn–1Ca and Zn–1Sr alloys from 10 to 30 MPa, 18–50 MPa and 0.3–5.8%; 130–192 MPa, 180–237 MPa and 2–9%; 119–206 MPa, 165–252 MPa and 2.1–12.7%, and 120–188 MPa, 171–229 MPa and 2–20%, respectively. However, H values remained almost steady for pure Zn and alloys except Zn–1Ca alloy. The sequence of CR is Zn < Zn–1Mg < Zn–1Ca < Zn–1Sr.	[147]
	Zn–4Ag	Melting at 750 °C with Ar gas protection + homogenization at 300 °C for 1 h followed by furnace cooling to RT + HR at 200 °C with total 70% reduction in diameter + annealed at 390 °C for 15 min + precipitation hardened in an oil bath for 10 min at 100 °C.	After thermomechanical treatment, σ_TYS_, σ_UTS_ and ε of the alloy are 157 MPa, 261 MPa, and 37%, respectively, rendering this alloy a promising material for bioresorbable stents.	[167]
	Pure Zn, Zn-5Ge	Melting at 500 °C with Ar gas protection + homogenization at 340 °C for 10 h followed by air cooling to RT + HR at 200 °C with total 80% reduction in thickness.	After hot rolling, the grains of the Zn–5Ge alloy were elongated along the deformation direction and the eutectic Ge phase was significantly refined. Hot rolling significantly increased the σ_TYS_, σ_UTS_, ε and CR of Zn-5Ge alloys from 48 to 175 MPa, 54–237 MPa, 1.1–22% and 0.127–0.225 mm/y, respectively, while the H values decreased from 68 to 60 Hv.	[168]
	Zn–1Cu-0.1Ti	Melting at 550–600 °C with Ar gas protection + homogenization at 340 °C for 10 h followed by air cooling to RT + rolling (HR) at 250 °C with total 80% reduction in thickness + cold rolling to a 40% total reduction in thickness.	HR and HR + CR Zn–1Cu-0.1Ti contained a η-Zn phase, a ε-CuZn_5_ phase, and an intermetallic phase of TiZn_16_. The HR + CR alloy exhibited a σ_TYS_ of 204 MPa, a σ_UTS_ of 250 MPa, and a ε of 75%; significantly higher than those of HR alloy. The CR in Hanks' solution was 0.032 mm/y for HR + CR alloy and 0.034 mm/y for HR alloy. The HR alloy showed the best wear resistance.	[152]
	Zn-0.8Mn-0.4Ca	Melting at 725 °C with Ar gas protection + homogenization at 360 °C for 6 h followed by water quenched + HR at 100 °C with total 64% reduction in thickness.	Hot rolling significantly refined Zn grains of Zn–Mn–Ca alloy from 289 to 5 μm and increased the σ_TYS_, σ_UTS_ and ε from 112 to 245 MPa, 120–323 MPa and 0.3–12%, respectively.	[169]
**ECAP (equal channel angular pressing)**	Pure Zn	Melting at 650 °C for 1 h + Annealing at 450 °C for 4 h + ECAP processing = 22 °C with extrusion rate (ER) of 0.1 mm/s + angle between channels (*ϕ*) = 90°	The ECAP caused GR of pure Zn with a mean GS decreased from 509 to 20 μm, leading to an increase in ε by ~ 45% and a decrease in σ_UTS_ from 97 to 91 MPa.	[170]
	Zn-0.8Ag	Melting at 650 °C for 1 h + Annealing at 450 °C for 4 h + ECAP processing = 22 °C with ER of 0.1 mm/s + *ϕ* = 90°	The alloy contained recrystallized, equiaxed grains with an average GS of ~2.7 μm, 2.6 μm, and 2.2 μ, displaying a ε of 390%, 448%, and 428% in X, Y, and Z directions, respectively.	[171]
	Zn-0.82Ag	Melting at 650 °C for 1 h + Annealing at 450 °C for 4 h + ECAP processing = 22 °C with ER of 0.1 mm/s + *ϕ* = 90°	ECAP caused grain refining to 3.2 μm and enhanced ε up to 245%. The GR increased grain boundary sliding, viscous glide, and diffusion creep that could be responsible for substantial ductility.	[170]
	Zn-0.3Al	Melting at 470 °C for 1 h + Homogenization at 320 °C for 12 h + Hot rolled = At 100 °C with a 35% reduction in thickness + ECAP processing = 22 °C with ER of 0.1 mm/s + *ϕ* = 90°	Multi-pass ECAP refined the coarse-grained (100–250 μm) microstructure into fine grains (~2 μm) and increased ε substantially (maximum 1000%).	[172]
	Zn-0.49Cu	Melting at 650 °C for 1 h + Annealing at 450 °C for 4 h + ECAP processing = 22 °C with ER of 0.1 mm/s + *ϕ* = 90°	ECAP resulted GR and increased ε by over 500%, but reduced σ_TYS_ and σ_UTS_ twice compared to the same alloys processed by HE. The GR concurrently raised the activity of grain boundary sliding, viscous glide and diffusion creep, leading to significant enhancement of ductility.	[170]
	Zn-0.5Cu	Melting at 650 °C for 1 h + Annealing at 450 °C for 4 h + ECAP processing = 22 °C with ER of 0.1 mm/s + *ϕ* = 90°	GR occurred due to four-pass ECAP with an average GS from 560 to 1 μm, leading to an increase in ε by 510%.	[173]
	Zn–3Mg	Melting at 550 °C for 1 h + homogenized at 370 °C for 15 h followed by WQ + ECAP processing = 22 °C with ER of 1 mm/s + *ϕ* = 120°	Two-pass ECAP led to GR decreased from 48 to 1.8 μm, which notable improved the σ_UTS_ and ε from 84 to 220 MPa and 1.3–6.3%, respectively, in addition to a decrease in CR from 0.30 to 0.24 mm/y.	[160]
	Zn-0.42Mn	Melting at 650 °C for 1 h + Annealing at 450 °C for 4 h + ECAP processing = 22 °C with ER of 0.1 mm/s + angle between channels = 90°	ECAP caused GR with mean GS decreased from 7.0 to 1.1 μm, resulting in an increase in ε by 108%, but a decrease in σ_TYS_ from 187 to 148 MPa and σ_UTS_ from 251 to 188 MPa, respectively.	[170]
**HPT (high-pressure torsion)**	Pure Zn	Pressure, *P* = 1 GPa, Rotation speed = 1 rpm, Number of turns, *N* = 0, 1, 3, 5.	Average GS increased from 59 to 80 μm and σ_TYS_ increased from 61 to 114 MPa with increasing *N*.	[156]
	Zn–Mg hybrids	Pressure, *P* = 6 GPa, Rotation speed = 1 rpm, Number of turns, *N* = 1, 5, 15, 30.	Homogenous microstructure obtained at 15 turns. GS decreased from 43 to 10 μm and H increased from 40 to 250 H V with increasing *N* from 1 to 30.	[174]
	Zn-0.5Cu	Pressure, *P* = 6 GPa, Rotation speed = 1 rpm, Number of turns, *N* = 0, ½, 1, 2, 5, 10.	HPT caused GR and texture sharpening, leading to an increase in ε by 285%. The σ_TYS_ and σ_UTS_ also increased with increasing *N*.	[175]

**Table 5:** Effect of alloying elements, fabrication techniques, and post thermomechanical processes on the microstructure and mechanical properties of various Zn-based alloys.

**Composition (wt.%)**	**Processing technique**	Microstructural properties		Mechanical properties						Ref.
		Main phase	GS (μm)	σ_TYS_ (MPa)	σ_UTS_ (MPa)	Ε (%)	σ_CYS_ (MPa)	H (HV)	E (GPa)	
**Pure Zn**										
**Zn**	Cast	α-Zn	–	10 ± 2	18 ± 3	0.3 ± 0.1	–	38 ± 1	–	[147]
	HR		–	30 ± 7	50 ± 9	5.8 ± 0.8	–	39 ± 4	–	
	HE		–	33 ± 7	64 ± 15	3.6 ± 1.8	103 ± 7	–	–	
	HE		151 ± 20	51 ± 4	111 ± 5	60 ± 6	–	34 ± 2	–	[178]
	SLM		–	114	134	10.1	–	42 ± 4	23 ± 9.2	[140]
	PM		–	–	–	16	33	18	9.1	[139]
	HE		100	–	24	0.3	–	25	–	[94]
	Cast		500	–	20	0.3	–	30	–	[214]
	HE		34	57 ± 8	112 ± 11	63 ± 12	–	–	–	[102]
	HE		20	55 ± 8	97 ± 10	7.7 ± 2.7	94 ± 13	44 ± 6	–	[164]
			82	60	117	14.0	–	–	–	[188]
	Cast		3094	28	30	0.6	–	33	–	[215]
	HR		122	85	118	26.7	–	33	–	
	HR	–	–	35 ± 6	49 ± 11	6 ± 2	–	40 ± 7	–	[216]
	HE	α-Zn	20	124	164	39.3	94	44		[213]
	SLM		104 ± 30	43 ± 3	61 ± 5	1.7 ± 0.1	–	50 ± 6	12 ± 2.4	[142]
**Zn plate**	HE + DW	–	–	–	35 ± 2	9.4 ± 1.2	–	38 ± 8	–	[71]
**Zn tube**		–	–	–	45 ± 2	7.6 ± 0.2	–	41 ± 7	–	
**Porous Zn**	LPBF (Hor)	α-Zn	–	55 ± 0.7	79 ± 0.6	12 ± 1.5	–	–	53 ± 3	[217]
	LPBF (Ver)		–	78 ± 0.4	100 ± 0.4	10 ± 0.5	–	–	88 ± 1.0	
**Zn Alloyed with nutrient elements (Ca, Mg and Sr)**										
**Zn-0.002 Mg**	HE + DW	α-Zn, Mg_2_Zn_11_	76 ± 10	34 ± 4	63 ± 9	17 ± 3	–	45 ± 1	–	[151]
**Zn-0.005 Mg**			7.2 ± 1.5	93 ± 1	202 ± 60	28 ± 2	–	93 ± 1	–	
**Zn-0.08 Mg**			6.3 ± 0.8	221 ± 14	339 ± 42	40 ± 3	–	103 ± 1	–	
**Zn-0.05 Mg**	HE		20	160	225	26	–	–	–	[148]
**Zn-0.1 Mg**			–	214	274	10.2	201	70	–	[213]
**Zn-0.4 Mg**			–	284	353	15.2	281	82	–	
**Zn-0.8 Mg**			–	297	386	9.3	304	96	–	
**Zn-0.15 Mg**			6.6 ± 0.8	114 ± 8	250 ± 9	22 ± 4	–	52 ± 5	–	[178]
**Zn-0.5 Mg**			4.1 ± 0.4	159 ± 9	297 ± 7	13 ± 1	–	65 ± 4	–	
**Zn-1.0 Mg**			4.4 ± 0.5	180 ± 7	340 ± 16	6 ± 1	–	75 ± 4	–	
**Zn-3.0 Mg**			–	291 ± 9	399 ± 14	1 ± 0.1	–	117 ± 6	–	
**Zn-0.02 Mg**	HE at 200 °C		45	132 ± 5	163 ± 8	26 ± 9	–	–	–	[218]
**Zn-0.05 Mg**			24	152 ± 5	232 ± 5	14 ± 2	–	–	–	
**Zn-0.2 Mg**			16	179 ± 5	234 ± 2	8 ± 1	–	–	–	
**Zn-0.5 Mg**			9	227 ± 5	268 ± 5	10 ± 3	–	–	–	
**Zn-1.0 Mg**			9	262 ± 10	326 ± 5	5 ± 1	–	–	–	
**Zn-0.02 Mg**	HE at 300 °C		117	103 ± 5	134 ± 5	6 ± 4	–	–	–	
**Zn-0.05 Mg**			85	122 ± 5	142 ± 5	3 ± 1	–	–	–	
**Zn-0.2 Mg**			38	170 ± 2	205 ± 2	1 ± 1	–	–	–	
**Zn-0.5 Mg**			14	209 ± 5	250 ± 5	1 ± 1	–	–	–	
**Zn-1.0 Mg**			11	252 ± 5	317 ± 5	2 ± 1	–	–	–	
**Zn-0.02 Mg**	Cast	–	25	136 ± 2	167 ± 6	27 ± 3	–	65 ± 4	–	[219]
**Zn-0.02 Mg**	HE + DW		1	388 ± 2	455 ± 2	5 ± 3	–	–	–	
**Zn-1.0 Mg**	Cast	α-Zn, MgZn_2_	–	130 ± 10	180 ± 21	2 ± 0.2	–	78 ± 3	–	[147]
	HE HR		–	210 ± 15	265 ± 16	9 ± 1	285 ± 17	–	–	
			–	192 ± 9	237 ± 20	13 ± 0.1	–	74 ± 4	–	
**Zn-0.8 Mg**	HE		20	203 ± 7	301 ± 8	13 ± 2	186 ± 10	83 ± 5	–	[164]
**Zn-1.6 Mg**			20	232 ± 8	368 ± 8	4 ± 0.3	257 ± 13	97 ± 4	–	
**Zn-1.0 Mg**	Cast		10	94 ± 5	138 ± 5	0.5 ± 0.1	–	–	–	[104]
	HE		10	180 ± 4	252 ± 6	13 ± 2	–	–	–	
**Zn-0.5 Mg**	Cast		–	–	134 ± 9	5 ± 0.7	–	67 ± 2	–	[134]
**Zn-1.0 Mg**			–	–	143 ± 15	3 ± 0.5	–	74 ± 2	–	
**Zn-2.0 Mg**			–	–	154 ± 37	2 ± 0.4	–	96 ± 4	–	
**Zn-5.0 Mg**		α-Zn, Mg_2_Zn_11_ MgZn_2_	–	–	–	–	–	101 ± 7	–	
**Zn-7.0 Mg**			–	–	–	–	–	106 ± 2	–	
**Zn-1.0 Mg**	Cast	α-Zn, Mg_2_Zn_11_	–	–	153	1.5	–	65	–	[214]
**Zn-1.5 Mg**			–	–	147	0.4	–	93	–	
**Zn-3.0 Mg**			–	–	28	0.2	–	206	–	
**Zn-1.2 Mg**	Cast		–	117 ± 1	130 ± 6	1.4 ± 0.6	–	93 ± 7	–	[220]
	HE		–	220 ± 15	363 ± 5	21 ± 2	–	96 ± 7	–	
**Zn-1.0 Mg**	HE		30	90 ± 20	155 ± 15	2 ± 0.2	–	65 ± 10	–	[94]
**Zn-1.5 Mg**			30	–	150 ± 25	1 ± 0.3	–	100 ± 10	–	
**Zn-3.0 Mg**			50	–	32 ± 9	0.2 ± 0.1	–	210 ± 10	–	
**Zn-1.0 Mg**	Cast		150	–	120	0.4	–	–	–	[154]
	HE		0.7	316	435	35	–	–	–	
**Zn-1.6 Mg**	Cast		35	–	172 ± 12	–	245 ± 12	82 ± 2	–	[221]
	HE		10	242 ± 14	365 ± 18	6 ± 2	292 ± 11	97 ± 3	–	
	RS + HE		2	332 ± 11	370 ± 16	9 ± 2	382 ± 15	122 ± 3	–	
**Zn-1.0 Mg**	SLM		10 ± 2.8	74 ± 4	126 ± 4	3.6 ± 0.2	–	93 ± 8	19 ± 3	[142]
**Zn-2.0 Mg**			6.7 ± 1.8	117 ± 5	162 ± 6	4.1 ± 0.2	–	134 ± 7	25 ± 3	
**Zn-3.0 Mg**			5.2 ± 1.3	152 ± 5	222 ± 8	7.2 ± 0.4	–	177 ± 9	48 ± 4	
**Zn-4.0 Mg**		α-Zn, Mg_2_Zn_11_, MgZn_2_	4.9 ± 1.4	132 ± 8	166 ± 7	3.1 ± 0.3	–	199 ± 9	58 ± 5	
**Zn-3.0 Mg**	Cast	α-Zn, Mg_2_Zn_11_	48	65 ± 9	84 ± 9	1.3 ± 0.3	–	200 ± 7	132 ± 4	[160]
	Hom		30	36 ± 3	46 ± 1	2.1 ± 0.1	–	175 ± 8	84 ± 3	
	1-pass ECAP		2.3	137 ± 2	153 ± 4	4.6 ± 0.5	–	180 ± 4	205 ± 9	
	2-pass ECAP		1.8	205 ± 4	220 ± 3	6.3 ± 0.9	–	186 ± 4	210 ± 8	
	Cast		–	–	104 ± 8	2.3 ± 0.3	–	201 ± 7	–	[222]
	Hom		–	–	88 ± 1	8.8 ± 0.1	–	175 ± 8	–	
**Zn-0.1 Mg**	Cast	α-Zn	160 ± 22	72 ± 8	81 ± 10	0.6 ± 0.3	–	45 ± 6	–	[215]
**Zn-0.2 Mg**		α-Zn, Mg_2_Zn_11_	100 ± 25	82 ± 9	100 ± 8	0.9 ± 0.4	–	47 ± 8	–	
**Zn-0.4 Mg**			70 ± 11	92 ± 11	108 ± 12	0.8 ± 0.4	–	53 ± 12	–	
**Zn-0.8 Mg**			65 ± 9	112 ± 11	125 ± 10	0.8 ± 0.4	–	71 ± 19	–	
**Zn-1.0 Mg**	PM	α-Zn, MgZn_2_	7.3	–	–	5.6 ± 1.4	245 ± 12	81 ± 5	80 ± 4	[138]
**Zn–25Mg**		α-Zn, Mg_2_Zn_11_, MgZn_2_	–	–	–	5.2 ± 1.5	403 ± 14	180 ± 8	86 ± 5	
**Zn–4Mg**	HR	–	–	236 ± 22	287 ± 21	16 ± 6	–	72 ± 11	–	[216]
**Zn-0.05 Mg**	HR	α-Zn, Mg_2_Zn_11_	0.4 ± 0.1	197 ± 4	227 ± 5	34 ± 3	–	–	–	[179]
**Zn-0.05Mg-0.1Mn**		α-Zn, Mg_2_Zn_11_, MnZn_13_	0.7 ± 0.2	230 ± 3	274 ± 5	41 ± 1	–	–	–	
**Zn-0.05Mg-0.5Cu**		α-Zn, Mg_2_Zn_11_, ε-CuZn_4_	0.5 ± 0.1	241 ± 5	312 ± 2	44 ± 2	–	–	–	
**Zn-1.5 Mg**	Cast	α-Zn, Mg_2_Zn_11_	–	120 ± 5	151 ± 13	1.3 ± 0.2	–	154 ± 23	–	[135]
**Zn-1.5Mg-0.1Ca**		α-Zn, Mg_2_Zn_11_, CaZn_13_	–	174 ± 15	241 ± 0.4	1.7 ± 0.1	–	150 ± 20	–	
**Zn-1.5Mg-0.1Sr**		α-Zn, MgZn_2_, SrZn_13_	–	130 ± 8	209 ± 10	2.0 ± 0.2	–	150 ± 20	–	
**Zn-1.5 Mg**	Cast	α-Zn, Mg_2_Zn_11_	–	–	150	–	245	93 ± 9	–	[223]
**Zn-1.5Mg-0.5Ca**		α-Zn, Mg_2_Zn_11_, CaZn_11_	–	93	128	–	317	122 ± 14	–	
**Zn-1.5 Mg**	HE	α-Zn, Mg_2_Zn_11_	–	242	367	–	315	101 ± 11	–	
**Zn-1.5Mg-0.5Ca**		α-Zn, Mg_2_Zn_11_, CaZn_11_	–	205	351	–	346	127 ± 9	–	
**Zn-0.5Mg-0.1Ca**	HE	α-Zn, Mg_2_Zn_11_, CaZn_13_	10–20	140 ± 7	273 ± 14	4.1 ± 0.2	–	85 ± 4	–	[180]
**Zn-1.0Mg-0.1Ca**			10–20	144 ± 5	370 ± 15	5.4 ± 0.2	–	100 ± 5	–	
**Zn-1.5Mg-0.1Ca**			10–20	160 ± 6	442 ± 18	4.9 ± 0.2	–	111 ± 7	–	
**Zn-1.0Mg-1.0Ca**	Cast		10–50	80 ± 9	131 ± 16	1.0 ± 0.3	–	92 ± 10	–	[55]
	HE		10–50	205 ± 10	257 ± 13	5.1 ± 1.0	300 ± 55	–	–	
	HR		10–50	138 ± 9	198 ± 20	8.5 ± 1.3	–	107 ± 10	–	
**Zn-1.0Mg-1.0Sr**	Cast	α-Zn, Mg_2_Zn_11_, SrZn_13_	10–50	87 ± 7	138 ± 9	1.3 ± 0.2	–	85 ± 2	–	
	HE		10–50	202 ± 5	253 ± 18	7.4 ± 1.3	383 ± 71	–	–	
	HR		10–50	140 ± 10	201 ± 10	9.7 ± 1	–	92 ± 5	–	
**Zn-0.02Mg-0.02Cu**	HE	α-Zn, Mg_2_Zn_11_,	13 ± 2	216 ± 3	262 ± 5	28 ± 2	–	74 ± 2	–	[224]
**Zn-1.0Mg-0.1Sr**	Cast	α-Zn, MgZn_2_, SrZn_13_	–	109 ± 14	132 ± 10	1.4 ± 0.4	–	94 ± 7	–	[225]
**Zn-1.0Mg-0.5Sr**			–	129 ± 5	144 ± 15	1.1 ± 0.1	–	109 ± 8	–	
**Zn-1.0Mg-0.1Sr**	HR	α-Zn, MgZn_2_, SrZn_13_	–	197 ± 13	300 ± 6	23 ± 3	–	104 ± 10	–	
**Zn-1.0Mg-0.1Mn**	Cast	α-Zn, MgZn_2_, Mn	–	114	132	1.1	–	98	–	[226]
	HR		–	195	299	26.1	–	108	–	
**Zn-1.5Mg-0.1Mn**	Cast		–	114	122	0.8	–	149	–	
**Zn–1Mg-0.1Zr**	HE	α-Zn, Mg_2_Zn_11_, Zn_22_Zr	–	248 ± 3	314 ± 2	2.5 ± 0.1	300 ± 2	94 ± 3	–	[227]
**Zn–1Mg-0.25Zr**			–	236 ± 2	300 ± 2	2.5 ± 0.1	289 ± 2	93 ± 3	–	
**Zn–1Mg-0.4Zr**			–	241 ± 4	316 ± 3	4.7 ± 0.1	301 ± 2	95 ± 4	–	
**Zn-0.1Ca**	HE	α-Zn, CaZn_13_	–	127	169	37.9	122	45	–	[213]
**Zn-0.4Ca**			–	116	166	26.7	111	44	–	
**Zn-0.8Ca**			–	127	173	27.9	111	44	–	
**Zn–1Ca**	Cast		–	119 ± 7	165 ± 14	2.1 ± 0.2	–	73 ± 7	–	[147]
	HE		–	200 ± 10	242 ± 10	7.7 ± 0.7	281 ± 29	–	–	
	HR		–	206 ± 7	252 ± 10	12.7 ± 1	–	63 ± 3	–	
**Zn–1Ca–1Sr**	Cast	α-Zn, CaZn_13_, SrZn_13_	10–50	86 ± 5	140 ± 9	1.2 ± 0.2	–	91 ± 12	–	[55]
	HE		10–50	212 ± 15	260 ± 15	6.7 ± 1.1	340 ± 43	–	–	
	HR		10–50	144 ± 9	203 ± 10	8.8 ± 1.2	–	87 ± 7	–	
**Zn-0.1Sr**	HE	α-Zn, SrZn_13_	–	89	139	34.5	88	44	–	[213]
**Zn-0.4Sr**			–	106	153	20.2	94	44	–	
**Zn-0.8Sr**			–	104	151	30.0	105	48	–	
**Zn–1Sr**	Cast		–	120 ± 6	171 ± 14	2.0 ± 0.2	–	62 ± 7	–	[147]
	HE		–	218 ± 6	264 ± 10	10.6 ± 1	341 ± 36	–	–	
	HR		–	188 ± 6	229 ± 10	20 ± 2	–	62 ± 5	–	
**Zn-1.1Sr**	HR	–	–	220 ± 25	250 ± 30	22 ± 4	–	74 ± 10	–	[216]
**Zn Alloyed with crucial elements (Cu, Fe and Mn)**										
**Zn-0.4Cu**	HE	α-Zn, CuZn_5_	–	150	197	40.2	139	59	–	[213]
**Zn-0.8Cu**			–	184	234	33.1	165	69	–	
**Zn–2Cu**			–	223	270	40.7	233	75	–	
**Zn–1Cu**	Cast		<237	26	33	3.9	–	–	–	[228]
**Zn–2Cu**			<237	50	60	3.4	–	–	–	
**Zn–4Cu**			<237	73	105	3.3	–	–	–	
**Zn–1Cu**	HR		27.5	236	291	38.9	–	–	–	
**Zn–2Cu**			27.5	275	328	48.8	–	–	–	
**Zn–4Cu**			27.5	327	393	44.6	–	–	–	
**Zn–1Cu**	HE		33.7	149 ± 1	186 ± 1	21 ± 4	–	–	–	[162]
**Zn–2Cu**			6.9	200 ± 4	240 ± 1	47 ± 1.4	–	–	–	
**Zn–3Cu**			4.7	213 ± 1	257 ± 1	47 ± 1	–	–	–	
**Zn–4Cu**			2.3	227 ± 5	271 ± 1	51 ± 3	–	–	–	
**Zn-0.5Cu**	HE		38 ± 19	131 ± 1	180 ± 1	27 ± 1	–	–	–	[170]
	ECAP		2.2 ± 1.7	48 ± 1	94 ± 1	345 ± 12	–	–	–	
**Zn–4Cu**	HE		–	250 ± 10	270 ± 10	51 ± 2	–	–	–	[149]
**Zn–3Cu**	HE		–	247 ± 8	288 ± 4	50 ± 3	–	67 ± 1	–	[105]
**Zn–3Cu-0.5Fe**		α-Zn, CuZn_5_, FeZn_13_	–	232 ± 3	284 ± 2	33 ± 4	–	76 ± 1	–	
**Zn–3Cu-1.0Fe**			–	222 ± 6	272 ± 7	20 ± 1.4	–	82 ± 1	–	
**Zn–3Cu**	HE	α-Zn, CuZn_5_	–	214	257	47.1	–	–	–	[163]
**Zn–3Cu-0.1 Mg**		α-Zn, CuZn_5_, Mg2Zn_11_	2–11	340 ± 15	360 ± 15	5 ± 1	–	–	–	
**Zn–3Cu-0.5 Mg**			2–11	400 ± 10	420 ± 5	2 ± 1	–	–	–	
**Zn–3Cu-1.0 Mg**			2–11	425 ± 5	440 ± 5	1 ± 0.5	–	–	–	
**Zn–1Cu-0.1Ti**	Cast	–	–	177	200	21	–	–	–	[86]
**Zn–1Cu-0.2Mn - 0.1Ti**		–	–	196	212	19	–	–	–	
**Zn–2Cu**	Cast	α-Zn, CuZn_5_	–	96	128	2.1	–	–	–	[229]
**Zn–2Cu-0.05Ti**		α-Zn, CuZn_5_, TiZn_16_		132	177	2.5				
**Zn–2Cu-0.1Ti**				113	146	1.8				
**Zn–1Cu-0.1Ti**	Cast	α-Zn, ε-CuZn_5_, TiZn_16_	54 ± 8	86 ± 3	92 ± 4	1.4 ± 0.8	–	73 ± 0.6	–	[152]
	Cast + HR		84 ± 13	175 ± 4	206 ± 6	39 ± 1.4	–	71 ± 1.8	–	
	HR + Cold rolling		–	204 ± 4	250 ± 4	75 ± 2	–	56 ± 0.8	–	
**Zn-0.1Fe**	HE	α-Zn, FeZn_11_	–	109	149	41.9	91	41	–	[213]
**Zn-0.4Fe**			–	123	159	47.4	108	42	–	
**Zn-0.8Fe**			–	127	163	28.1	111	53	–	
**Zn-0.3Fe**	Cast	α-Zn, FeZn_13_	24.4	–	47.2 ± 2.6	0.14 ± 0.02	112 ± 7	–	–	[192]
	BCWC		7.5	70.5 ± 0.7	76.4 ± 2.0	1.18 ± 0.04	117 ± 4	–	–	
**Zn-1.3Fe**	Cast	α-Zn, Zn_11_Fe	–	80 ± 4.6	134 ± 1.3	1.8 ± 0.4	–	56 ± 2	–	[230]
**Zn-0.1Mn**	HE	α-Zn, MnZn_13_	–	131	177	39.8	125	54	–	[213]
**Zn-0.4Mn**			–	160	214	43.6	136	57	–	
**Zn-0.8Mn**			–	156	190	83.8	145	50	–	
**Zn-0.1Mn**			–	132 ± 3	178 ± 8	40 ± 3.7	129 ± 11	55 ± 0.7	–	[231]
**Zn-0.4Mn**			–	162 ± 9	215 ± 5	44 ± 1.6	136 ± 7	58 ± 3.2	–	
**Zn-0.8Mn**			–	158 ± 7	191 ± 11	84 ± 1.9	142 ± 6	51 ± 1.2	–	
**Zn-0.2Mn**			4	132	220	48	–	–	–	[188]
**Zn-0.4Mn**			3	123	198	54	–	–	–	
**Zn-0.6Mn**			2	118	182	71	–	–	–	
**Zn-0.42Mn**			7.0 ± 3.2	187 ± 1	251 ± 1	24 ± 2	–	–	–	[170]
**Zn-0.42Mn**	ECAP		1.1 ± 0.6	148 ± 8	188 ± 6	93 ± 3	–	–	–	
**Zn-4.0Mn**	PM	α-Zn, MnZn_13_, MnZn_3_	<100 nm	–	–	14.9	291	102	–	[139]
**Zn-24.0Mn**		α-Zn, MnZn_3_, Mn_0__·__27_Zn_0.73_	<100 nm	–	–	6.7	132	71	–	
**Zn-0.34Mn**	Cast	α-Zn, MnZn_13_	420	98 ± 4	105 ± 4	0.4 ± 0.1	–	–	–	[189]
	HR		–	155 ± 13	226 ± 10	37 ± 10	–	–	–	
	HR + Cold rolling		2.1	118 ± 6	167 ± 4	76 ± 1.2	–	–	–	
	HR + AC + Cold rolling		–	120 ± 3	167 ± 1	89 ± 8	–	–	–	
	HR + WC + Cold rolling		–	184 ± 2	234 ± 2	54 ± 4	–	–	–	
**Zn-0.76Mn**	Cast		187	130 ± 4	155 ± 8	1.8 ± 0.1	–	–	–	
	HR		–	137 ± 17	191 ± 18	46 ± 13	–	–	–	
	HR + Cold rolling		1.9	95 ± 6	141 ± 4	48 ± 10	–	–	–	
	HR + AC + Cold rolling		–	114 ± 1	153 ± 2	92 ± 16	–	–	–	
	HR + WC + Cold rolling		–	142 ± 4	194 ± 4	91 ± 18	–	–	–	
**Zn-0.5Mn**	Multi-pass extrusion		0.35	–	–	236	–	–	–	[190]
**Zn-0.8Mn**	Cast		2.0 ± 0.5	98 ± 2	105 ± 2.6	1.0 ± 0.3	–	–	–	[232]
	HE		1.6 ± 0.8	127 ± 2.4	219 ± 0.5	62 ± 4.4	–	–	–	
**Zn-0.8Mn - 0.4Ag**	Cast		2.2 ± 1.4	33 ± 3.2	57 ± 5.5	0.1 ± 0.1	–	–	–	
	HE		2.0 ± 0.9	156 ± 6	251 ± 7.3	63 ± 4.2	–	–	–	
	HR		2.0–4.0	173 ± 1.1	262 ± 1.7	46 ± 9.5	–	–	–	[233]
**Zn-0.8Mn - 0.4Cu**	Cast		3.0 ± 1.5	113 ± 0.2	117 ± 3.2	0.4 ± 0.1	–	–	–	[232]
	HE		1.1 0.8	191 ± 4.1	308 ± 0.6	39 ± 5.4	–	–	–	
**Zn-0.8Mn - 0.4Ca**	Cast	α-Zn, MnZn_13_, CaZn_13_	2.8 ± 0.9	112 ± 3.4	120 ± 6.3	0.3 ± 0.1	–	–	–	[169]
	HE		2.6 ± 1.0	253 ± 1.3	343 ± 1.6	8 ± 1.4	–	–	–	
	HR		2.5–2.8	245 ± 5.7	323 ± 11	12 ± 0.9	–	–	–	
**Zn-0.35Mn - 0.41Cu**	Cast	α-Zn, MnZn_13_, MnCuZn_18_	7.5 ± 2.6	77	84	0.3 ± 0.1	–	–	–	[145]
	HR		1.1 ± 0.4	198 ± 7	292 ± 3	30 ± 3.8	–	–	–	
**Zn-0.75Mn - 0.40Cu**	Cast		7.9 ± 4.8	113	120 ± 3	0.4 ± 0.1	–	–	–	
	HR		1.2 ± 0.3	196 ± 11	278 ± 4	15.3 ± 4	–	–	–	
**Zn–1Mn - 0.1Fe**	Cast	α-Zn, MnZn_13_, FeZn_13_	384	92 ± 8	99 ± 3	0.2 ± 0.1	–	–	–	[234]
**Zn–1Mn - 0.5Fe**			384	96 ± 2	98 ± 2	0.2 ± 0.1	–	–	–	
**Zn–1Mn - 0.1Fe**	HR		–	122 ± 38	162 ± 15	37 ± 3	–	–	–	
**Zn–1Mn - 0.5Fe**			–	114 ± 7	157 ± 4	7.7 ± 5.9	–	–	–	
**Zn–1Mn - 0.1Ti**	Cast	–	–	180	198	7	–	–	–	[86]
**Zn Alloyed with other elements**										
**Zn-0.4Ag**	HE	α-Zn, ε-AgZn_3_	–	127	167	38.1	88	50	–	[213]
**Zn-0.8Ag**			–	134	184	58.3	82	58	–	
**Zn-2.0Ag**			–	186	231	36.7	145	55	–	
**Zn-2.5Ag**			16	147 ± 7	203 ± 5	35 ± 4	–	–	–	[102]
**Zn-5.0Ag**			4.2	210 ± 10	252 ± 7	37 ± 3	–	–	–	
**Zn-7.0Ag**			1.5	236 ± 12	287 ± 13	32 ± 2	–	–	–	
**Zn-4.0Ag**	Cast + TT		–	157	261	37	–	73	–	[167]
	Cast + TT + PH		–	149	215	24	–	82	–	
**Zn-0.8Ag**	ECAP (X-direction)		2.7 ± 1.2	37 ± 0.8	83 ± 0.5	390 ± 22	75 ± 2.6	–	–	[171]
	ECAP (Y-direction)		2.6 ± 1.3	38.5 ± 2.7	83.4 ± 2.7	448 ± 68	38.5 ± 2.7	–	–	
	ECAP (Z-direction)		2.2 ± 1.0	37.9 ± 2.3	92.7 ± 1.6	428 ± 23	37.9 ± 2.3	–	–	
**Zn-0.82Ag**	Ext		50 ± 25	114 ± 1	160 ± 1	18 ± 1	–	–	–	[170]
	ECAP		3.2 ± 2.6	76 ± 2	96 ± 1	143 ± 7	–	–	–	
**Zn–2Ag**	SLM		83	–	–	–	199 ± 28	55 ± 5	–	[141]
**Zn–4Ag**			55	–	–	–	216 ± 19	80 ± 4	–	
**Zn–6Ag**			25	–	–	–	293 ± 55	80 ± 3	–	
**Zn–8Ag**			45	–	–	–	267 ± 29	78 ± 4	–	
**Zn–4Ag**	HE		18.0	–	228 ± 5	27 ± 3	–	–	–	[235]
**Zn–4Ag-0.2Mn**			–	–	267 ± 9	25 ± 6	–	–	–	
**Zn–4Ag-0.4Mn**			2.4	–	281 ± 5	29 ± 3	–	–	–	
**Zn–4Ag-0.6Mn**			1.8	–	302 ± 7	35 ± 4	–	–	–	
**Zn-0.5Al**		α-Zn	19.4 ± 2.8	119 ± 2	203 ± 10	33 ± 1.2	–	59 ± 6	–	[178]
**Zn-1.0Al**			14.4 ± 1.2	113 ± 6	223 ± 4	24 ± 4.2	–	73 ± 5	–	
**Zn-1.0Al**	HR	–	–	197	238	24.0	–	–	–	[146]
**Zn-3.0Al**				202	223	31.0	–	–	–	
**Zn-5.0Al**				240	308	16.0	–	–	–	
**Zn-2.0Al**	LPBF	α-Zn, α-Al	5.53	142 ± 4	192 ± 5	12 ± 2	–	65 ± 2	–	[143]
										
**Zn-0.5Al**	Cast	α-Zn	–	–	79 ± 2	1.5 ± 0.1	–	71 ± 2	–	[136]
**Zn-0.5Al-0.1 Mg**		α-Zn, Mg_2_(Zn, Al)_11_	–	–	87 ± 3	1.6 ± 0.1	–	79 ± 3	–	
**Zn-0.5Al-0.3 Mg**			–	–	93 ± 3	1.7 ± 0.1	–	87 ± 3	–	
**Zn-0.5Al-0.5 Mg**			–	–	102 ± 4	2.1 ± 0.1	–	94 ± 4	–	
**Zn-4.0Al-1.0Cu**	HE		–	171	210	1	–	80	–	[94]
**Zn-0.5Al-0.5 Mg**	Cast	α-Zn, Mg_2_(Zn, Al)_11_, α-Mg_3_Bi_2_	–	–	92 ± 2	1.7 ± 1.6	–	94 ± 4	–	[205]
**Zn-0.5Al-0.5Mg-0.1Bi**			–	–	102 ± 4	2.4 ± 0.3	–	102 ± 5	–	
**Zn-0.5Al-0.5Mg-0.3Bi**			–	–	108 ± 4	2.7 ± 0.3	–	109 ± 5	–	
**Zn-0.5Al-0.5Mg-0.5Bi**			–	–	98 ± 3	2.0 ± 0.2	–	99 ± 4	–	
**ZA4-1 (3.5**–**4.5Al, 0.75**–**1.25Cu, 0.03**–**0.08 Mg)**	HE	α-Zn, α-Al	–	80 ± 6	187 ± 12	170 ± 11	161 ± 9	52 ± 3	–	[236]
**ZA4-3 (3.5**–**4.3Al, 2.5**–**3.2Cu, 0.03**–**0.06 Mg)**			–	110 ± 12	201 ± 14	126 ± 10	167 ± 6	56 ± 2	–	
**ZA6-1 (5.6**–**6.0Al, 1.2**–**1.6Cu)**			–	130 ± 9	228 ± 14	111 ± 13	233 ± 8	67 ± 7	–	
**Zn-5Ge**	Cast	α-Zn, eutectic Ge	32.2	48 ± 2	54 ± 0.7	1.1 ± 0.2	–	68 ± 2	–	[168]
	HR		–	175 ± 2	237 ± 3	22 ± 2.8	–	60 ± 1.7	–	
**Zn-0.1Li**	HE	α-Zn, LiZn_4_	21.7 ± 11	189 ± 5	230 ± 1	4 ± 0.6	–	–	–	[211]
**Zn-0.3Li**			6.4 ± 1.7	292 ± 4	367 ± 7	19 ± 1	–	–	–	
**Zn-0.4Li**			5.9 ± 1.4	364 ± 9	405 ± 7	27 ± 11	–	–	–	
**Zn-0.1Li**			–	341	431	28.1	306	108	–	[213]
**Zn-0.4Li**				387	520	5.0	434	164		
**Zn-0.8Li**			–	–	–	–	454	216	–	
**Zn-0.1Li**	HE + DW		3 ± 0.5	238 ± 60	274 ± 61	17 ± 7	–	97 ± 2	–	[101]
**Zn-0.2Li**	HR		50	240 ± 10	360 ± 15	14 ± 2	–	98 ± 6	–	[103]
**Zn-0.4Li**			10	425 ± 15	440 ± 5	14 ± 3	–	115 ± 7	–	
**Zn-0.7Li**			10	475 ± 50	565 ± 2	2.4 ± 0.4	–	137 ± 8	–	
**Zn-0.5Li**	HE		10	–	365	22	–	–	–	[237]
**Zn-0.5Li (mini tube)**			10	–	296	33	–	–	–	
**Zn-0.1Li**			26	189 ± 5	230 ± 0.4	4.0 ± 0.6	–	–	–	[238]
**Zn-0.8Li**	HR		4.1	186 ± 5	238 ± 5	75 ± 6	–	–	–	[239]
**Zn-0.8Li**	Cast	α-Zn, LiZn_4_	30–100	195 ± 21	214 ± 34	0.2 ± 0.1	–	–	–	[240]
	HWR		0.3–0.7	262 ± 41	401 ± 52	81 ± 10	–	–	–	
**Zn-0.8Li-0.2Ag**	HR	α-Zn, LiZn_4_, AgZn	2.3	196 ± 5	255 ± 4	98 ± 9	–	–	–	[239]
**Zn-0.8Li-0.2 Mg**	HR	α-Zn, LiZn_4_, Mg_2_Zn_11_	–	254 ± 5	341 ± 5	31 ± 6	–	–	–	
**Zn-0.1Li**	HE	α-Zn, ε-LiZn_4_	–	346	431	27.8	–	–	–	[213]
**Zn-0.1Li-0.4 Mg**		α-Zn, ε-LiZn_4_, Mg_2_Zn_11_	–	334	389	1.74	–	–	–	
**Zn-0.1Li-0.8 Mg**			–	356	412	2.41	–	–	–	
**Zn-0.4Li**		α-Zn, ε-LiZn_4_	–	389	519	5.62	–	–	–	
**Zn-0.4Li-0.4 Mg**		α-Zn, ε-LiZn_4_, Mg_2_Zn_11_	–	376	502	4.49	–	–	–	
**Zn-0.4Li-0.8 Mg**			–	401	520	1.56	–	–	–	
**Zn-0.8Li-0.4 Mg**			–	438	646	3.68	–	–	–	
**Zn-0.8Li-0.8 Mg**			–	429	462	1.83	–	–	–	
**Zn-0.1Li**	HE	α-Zn, ε-LiZn_4_	–	346	431	27.8	–	–	–	[213]
**Zn-0.1Li-0.1Mn**		α-Zn, ε-LiZn_4_, MnZn_13_	–	280	412	59.2	–	–	–	
**Zn-0.1Li-0.4Mn**			–	299	427	52.8	–	–	–	
**Zn-0.1Li-0.8Mn**			–	241	361	69.4	–	–	–	
**Zn-0.4Li**		α-Zn, ε-LiZn_4_	–	389	519	5.62	–	–	–	
**Zn-0.4Li-0.1Mn**		α-Zn, ε-LiZn_4_, MnZn_13_	–	276	449	63.3	–	–	–	
**Zn-0.4Li-0.4Mn**			–	322	458	75.3	–	–	–	
**Zn-0.4Li-0.8Mn**			–	270	443	70.3	–	–	–	
**Zn-0.8Li-0.1Mn**			–	367	551	63.8	–	–	–	
**Zn-0.8Li-0.4Mn**			–	336	493	73.2	–	–	–	
**Zn-0.8Li-0.8Mn**			–	357	513	103.5	–	–	–	
**Zn-0.01Ti**	Cast	α-Zn	600	64 ± 1	101 ± 4	8.5 ± 3.2	–	38 ± 2	–	[241]
**Zn-0.1Ti**		α-Zn, Zn_16_Ti	87	68 ± 2	115 ± 3	12.5 ± 4.4	–	44 ± 3	–	
**Zn-0.3Ti**			23	87 ± 6	141 ± 5	3.2 ± 0.3	–	53 ± 5	–	
**Zn-0.5Ti**			–	81 ± 1	150 ± 5	4.3 ± 1.0	–	51 ± 3	–	
**Zn-1.0Ti**			–	122 ± 1	177 ± 7	2.3 ± 0.3	–	65 ± 7	–	
**Zn-0.01Ti**	HE	α-Zn	14	177 ± 24	269 ± 5	10.9 ± 0.4	–	72 ± 2	–	
**Zn-0.1Ti**		α-Zn, Zn_16_Ti	–	163 ± 13	207 ± 3	43.8 ± 1.9	–	60 ± 4	–	
**Zn-0.3Ti**			–	143 ± 6	199 ± 2	29.8 ± 1.4	–	54 ± 2	–	
**Zn-0.01Zr**		α-Zn, Zn_22_Zr	93 ± 42	72	123	13	–	33	–	[194]
**Zn-0.02Zr**			97 ± 52	78	131	12	–	36	–	
**Zn-0.05Zr**			42 ± 26	100	157	22	–	38	–	
**Zn-0.1Zr**			45 ± 27	100	157	24	–	32	–	

HE: Hot extrusion; HR: hot rolling; Hom: homogenization; DW: Drawing; PM: powder metallurgy; RS: rapid solidification; SLM: selective laser melting; AC: Air cooling; WC: Water cooling; TT: thermal treatment; PH: precipitation hardening; ECAP: equal channel angular pressing; HWR: hot warm rolling; GS: grain size; σ_TYS:_ tensile yield strength; σ_UTS:_ ultimate tensile strength; ε: strain; σ_CYS:_ compressive yield strength; E: Young's modulus; H: Vickers hardness. BCWC: Bottom circulating water-cooled casting.

#### 2.1.4 Spark plasma sintering technique

Spark plasma sintering (SPS), also called pulsed electric current (PEC) sintering, is typically used to fabricate metal matrix micro/nanocomposites [118]. This technique is a modified form of the HP process and involves the usage of joule heating generated by the passage of a high DC pulsed current through a graphite die and specimen [119]. The SPS process contains a single operation to perform compaction and sintering of the powder sample and is efficient in simultaneously applying high pressure and rapid heating and cooling owing to the effects of PEC and spark plasma. Hence, full densification of powder samples can be obtained within a short duration and at a lower temperature as compared to conventional PM [120]. However, only simple symmetrical shapes can be fabricated using SPS technique. Čapek et al. [100] fabricated porous Zn using the SPS technique for the manufacture of biodegradable scaffold materials. They reported that the grain size of the starting powder did not affect the porosity of the fabricated porous Zn; however, it influenced the pore size and shape significantly, as well as the mechanical and corrosion properties (Table 3).

#### 2.1.5 Additive manufacturing

Additive manufacturing (AM), or three-dimensional (3D) printing, is currently attracting a great deal of attention in manufacturing metallic, polymeric, and ceramic biomaterials [121]. AM is particularly advantageous for creating complex 3D parts in a layer-by-layer manner with high precision and is also beneficial in the production of net-shaped or near-net-shaped components and for rapid prototyping [122,123]. AM technology can also produce patient-specific implants to fulfil a patient's specific requirements [124]. However, AM is still not an efficient way of producing a high volume of metallic parts because of its high production cost. There are two frequently employed AM techniques for manufacturing metallic biomaterials and medical devices; one is laser powder bed fusion (LPBF), which comprises selective laser melting (SLM), selective laser sintering (SLS), and electron beam melting (EBM); and the other is direct energy deposition [125,126]. Moreover, other AM techniques have also been developed such as direct metal writing, binder jetting, friction stir welding, diode-based processes, and cold spraying [[127], [128], [129]]. The fabrication of biodegradable Zn and Zn alloys via AM is showing promise in the biomaterials field [[130], [131], [132], [133]]. Some AM Zn alloys are summarized in Table 3.

### 2.2 Post-thermomechanical processing

The microstructures and resultant mechanical properties of Zn-based BMs can be tailored by the application of various processing techniques. The following section summarizes some fabrication methods that have been employed to develop Zn-based BMs.

#### 2.2.1 Conventional metal-forming processing (extrusion, drawing, rolling, and forging)

Conventional metal-forming processing (CMFP) uses applied mechanical force to deform a metal plastically to create the required product shape, generally with enhanced mechanical properties [144]. Various types of raw materials can be processed by CMFP and good surface finish can be achieved. However, limited products can be produced since only one type of cross section can be processed at a time. The CMFP techniques consist of extrusion, drawing, rolling, and forging. CMFP breaks down the as-cast microstructures and improves the mechanical properties via the activation of plastic-deformation mechanisms, namely, dislocation slip and twinning. CMFP can be subdivided into hot working and cold working. In hot working, the metal is plastically shaped at a temperature higher than its recrystallization temperature, while in cold working the metal is shaped below its recrystallization temperature. Most biodegradable Zn-based alloys can be shaped into flat plates through hot rolling (HR), which involves the passing of a heated metal sheet between one or more pairs of rollers, rotating at an identical speed but in reverse directions, to shrink the thickness [103,145,146]. In general, as-cast Zn alloys are homogenized in temperatures ranging from 250 °C to 350 °C for 30–180 min to acquire compositional uniformity and the thickness of the HR product varies from approximately 300 μm to several millimeters [147]. The extrusion and drawing processes can be used to process biodegradable Zn alloys with a cylindrical profile, such as a tube [[148], [149], [150]]. The processing of Zn alloys using hot extrusion (HE) includes pushing a metal billet via a die through an orifice of the desired shape. Metal processing via drawing is analogous to extrusion except that the metal is pulled via the die, rather than pushed. The metal billets are mostly pre-heated in both processes at 150–300 °C for 30–180 min prior to shaping [151]. In general, CMFP (e.g., extrusion, rolling, and drawing) leads to a better combination of mechanical properties (i.e., higher σ_UTS_ and ε) as compared to the mechanical properties of their as-cast counterparts. For example, the σ_UTS_ and ε of as-cast Zn–1Ca were concurrently enhanced from 165 MPa to 2.1% to 242 MPa and 7.7%, by HE, and to 252 MPa and 12.7% by HR, respectively [147]. Lin et al. [152] also reported an improvement in the mechanical properties of as-cast Zn–1Cu-0.1Ti alloy by HR, in which the σ_YTS_, σ_UTS_ and ε increased from 86 MPa, 92 MPa, and 1.4% to 175 MPa, 206 MPa, and 39.0%, respectively. It can be seen from the data listed in Table 5 that most of the Zn-based alloys fulfilling the mechanical benchmark criteria of biodegradable implant materials were processed via thermo-mechanical processing techniques including HE [153], HE + DW [151], and hydrostatic extrusion [154]. Consequently, extrusion and extrusion-based processing techniques are identified as the most effective approaches in producing biodegradable Zn-based alloys with the desired set of mechanical properties.

#### 2.2.2 Severe plastic deformation technique

The processing of materials via the severe plastic deformation technique (SPDT) involves metal-forming processes where an extremely high plastic strain can be put onto a bulk material using large-scale hydrostatic pressure, without any great variation in the overall dimensions of the solid. This method can produce final products from metals and alloys with very good grain refinement and ductility [155,156]. Also, complex shapes can be fabricated from a single piece with fine details and close tolerances which eliminates requirement of secondary machining operations. Weight and material savings can be realized because of formability of the material. However, SPD methods would be effective not only for investigations in laboratory scales but also for making the possibility of producing ultrafine-grained or nanostructured samples in industrial applications. Several SPDTs have been developed for processing different materials such as equal-channel angular pressing (ECAP), twist extrusion, high-pressure torsion (HPT), friction-stir processing, multi-directional forging, and cylinder-covered compression [157,158]. In fact, the exceptional grain refinement achieved in SPD-processed Zn-based materials simultaneously improves their mechanical properties and corrosion resistance [49]. However, the effect of severe plastic deformation techniques (SPDTs) on the tensile properties of Zn alloys has not been extensively investigated, to date. This could be due to the small sizes of the SPD-processed samples or the softening of pure Zn at high strains due to dynamic recrystallization [159]. Nevertheless, two-pass ECAP processing on Zn–3Mg alloy led to grain refinement (GR) as the grain size was decreased from 48 μm to 1.8 μm, which resulted in a significant increase in σ_UTS_ from 84 MPa to 220 MPa and ε from 1.3% to 6.3%, respectively, in addition to a decrease in CR from 0.30 to 0.24 mm/y [160]. HPT processing on these alloys also resulted in GR and texture sharpening, leading to an increase in ε by 285%. The resultant σ_TYS_ and σ_UTS_ were also increased by increasing the number of turns (*N*) during HPT. In another study, it was reported that HPT developed bulk-state reactions in hybrids Zn–3Mg alloys which simultaneously improved the hardness and ductility of these alloys [161]. The preparation of Zn-based alloys via ECAP and HPT has been reported by several group of researchers and the results obtained are summarized in Table 4, Table 5.

## 3 Microstructure, textural evolution, and mechanical properties of Zn-based alloys

### 3.1 Zn alloys containing nutrient elements

The elements that can drive biological activity and are indispensable in the human body are termed nutrient elements. The common nutrient elements are Mg, Ca and strontium (Sr). These nutrient elements are effective in boosting mechanical properties of pure Zn. Therefore, pure Zn is generally alloyed with various nutrient elements to achieve suitable mechanical and corrosion properties. The maximum solubility of Mg in Zn at 364 °C is almost 0.1 (wt.%) and at RT its solubility is almost negligible; therefore, the addition of Mg to a Zn matrix results in the formation of intermetallic compounds such as Mg_2_Zn_11_ [176].

**Fig. 2:**
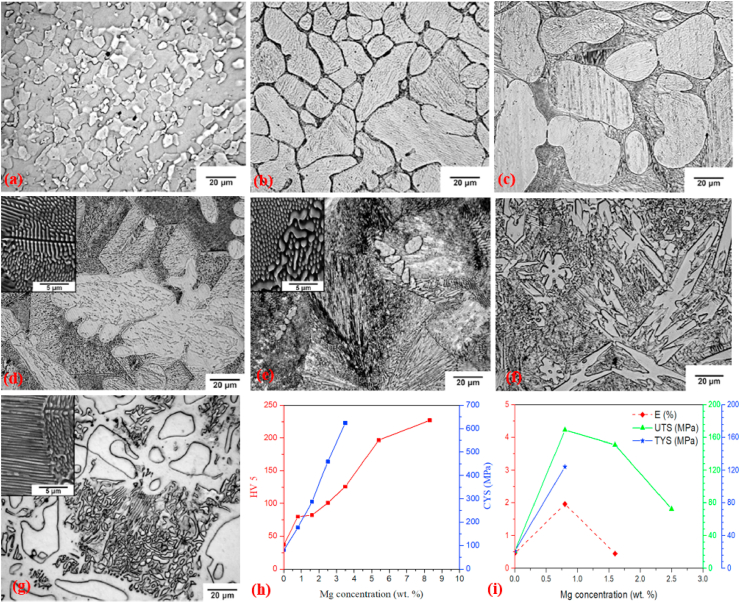
Optical micrographs (with SEM image insets) and mechanical properties of Zn alloys: (a) pure Zn, (b) Zn-0.8 Mg, (c) Zn-1.6 Mg, (d) Zn-2.5 Mg, e) Zn-3.5 Mg, (f) Zn-5.4 Mg, (g) Zn-8.3 Mg, (h) changes in hardness and compressive yield strength of Zn–Mg alloys with respect to Mg concentrations, (i) changes in tensile properties of Zn–Mg alloys with respect to Mg concentrations. (Reproduced with permission from Refs. [177]).

Kubásek et al. [177] investigated a series of biodegradable binary Zn–Mg alloys containing different Mg concentrations (0–8.3 wt%) and the microstructural and mechanical properties obtained are presented in Fig. 2. The microstructure of pure Zn containing α-Zn dendrites are shown in Fig. 2a. Addition of 0.8–2.5 wt% Mg to Zn matrices resulted in the formation of hypoeutectic microstructures, as shown in Fig. 2b–d. These microstructures comprised *α*-Zn dendrites and a eutectic mixture of *α*-Zn and Mg_2_Zn_11_ phases dominated by lamellar and rod morphologies, as shown in the inset of Fig. 2d. As the alloying composition of the Zn-3.5 Mg alloys approached the eutectic point of the Zn–Mg phase diagram, the resultant microstructures were dominated by a very fine rod-and-lamellar *α*-Zn + Mg_2_Zn_11_ eutectic mixture (Fig. 2e). In contrast, the microstructures of Zn-5.4 Mg and Zn-8.3 Mg alloys were found to be hypereutectic, with sharp-edged intermetallic phases of Mg_2_Zn_11_ and a eutectic combination of *α*-Zn + Mg_2_Zn_11_, as shown in Fig. 2f and g. The volume fraction of the brittle Mg_2_Zn_11_ intermetallic phase was increased by increasing the Mg content in the Zn matrices. The presence of hard intermetallic particles (Mg_2_Zn_11_) significantly enhanced the compressive yield strength and hardness of the Zn matrices, as shown in Fig. 2h, whereas the addition of 0.8 wt% Mg to the Zn matrix increased its ultimate tensile strength (σ_UTS_) up to 170 MPa, showing an overall increase of 465% over that of pure Zn (30 MPa). However, higher Mg concentrations (>0.8 wt%) in the Zn decreased the tensile properties of these alloys, as shown in Fig. 2i.

**Fig. 3:**
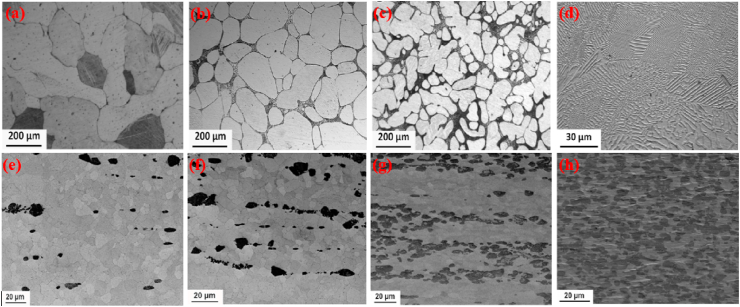
Optical micrographs of Zn-xMg alloys: (a) as-cast Zn-0.15 Mg, (b) as-cast Zn-0.5 Mg, (c) as-cast Zn-1.0 Mg, (d) as-cast Zn-3.0 Mg alloys, (e) hot-extruded Zn-0.15 Mg, (f) hot-extruded Zn-0.5 Mg, (g) hot-extruded Zn-1.0 Mg, and (h) hot-extruded Zn-3.0 Mg alloys. (Reproduced with permission from Refs. [178]).

Mostaed et al. [178] studied microstructural changes in as-cast and extruded Zn alloys containing different Mg concentrations (0.15–3.0 wt%) and the optical micrographs obtained are shown in Fig. 3. In the as-cast Zn-xMg (x = 0.15, 0.50, and 1.00 wt%) alloys, hypoeutectic microstructures were composed of elementary α-Zn dendritic grains in a eutectic matrix of Zn and Mg_2_Zn_11_ phases (Fig. 3a–c). The volume fraction of the Mg_2_Zn_11_ phase was simultaneously increased by increasing the Mg concentration from 0.15 to 1 wt% in the Zn matrices, resulting in their grain refinement, whereas in the Zn–Mg alloys containing 3 wt% Mg, a fully eutectic structure was observed along with thin lamellar phases of Zn and Mg_2_Zn_11_ (Fig. 3d). The microstructures of the hot-extruded Zn–(0.15–3 wt.%) Mg alloys are shown in Fig. 3e–h. It can be observed that with increasing wt.% of Mg, the volume fraction of the dark intermetallic Mg_2_Zn_11_ particles was successively increased and eventually these particles were uniformly dispersed in the microstructure of the Zn–3Mg alloy (Fig. 3h), which complements the results reported by Jin et al. [151] for extruded and drawn Zn–Mg alloys.

**Fig. 4:**
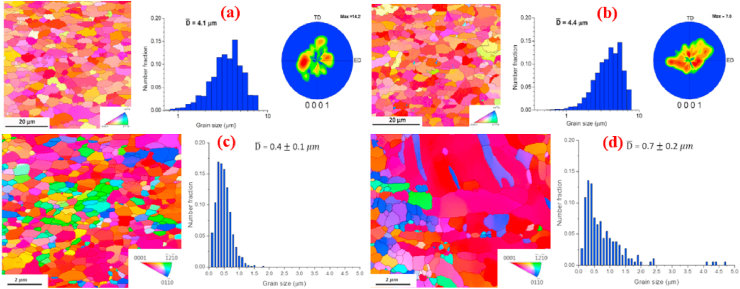
EBSD orientation maps, grain size distributions and (0001) pole figures of extruded Zn-xMg alloys: (a) Zn-0.5 Mg, (b) Zn-1.0 Mg, (c) Zn-0.05 Mg, and (d) Zn-0.05Mg-0.1Mn alloys. (Reproduced with permission from Refs. [178,179]).

Fig. 4a and b shows the crystallographic textural analysis and grain orientation maps obtained via electron backscattered diffraction (EBSD) analysis of the hot-extruded Zn–Mg alloys. It can be seen from the grain size distribution that in the case of Zn–Mg alloys containing 0.5 and 1.0 wt% Mg, HE caused an alteration in their microstructure from dendritic to equiaxed (with mean grain sizes of 4.1 ± 0.4 and 4.4 ± 0.5 mm, respectively). The textural analysis demonstrated that the HE Zn alloys developed textures with (0001) tilted a couple of degrees from the extrusion direction. Similarly, Xiao et al. [148] reported changes in the microstructures and mechanical properties of extruded Zn alloys containing tiny fractions of Mg (0.05 wt%). Compared to pure Zn, the microstructure of extruded Zn-0.05 Mg was composed of smaller grains. Moreover, even the addition of small fractions of Mg (0.05 wt%) to the Zn matrices resulted in the formation of an intermetallic Mg_2_Zn_11_ phase which was distributed uniformly in the Zn matrix. The addition of 0.05 wt% Mg to the extruded Zn significantly increased its σ_UTS_ to 225 MPa and its elongation (ε) to 26%, showing increases of more than ~2 times in σ_UTS_ and ~1.9 times in ε over extruded pure Zn (with σ_UTS_ = 112 MPa and elongation = 14%). Ardakani et al. [179] reported the effects of the addition of 0.1 wt% of Mn on the microstructure (Fig. 4c–d) and tensile properties of binary Zn-0.05 Mg alloys. They also found that the microstructure of a Zn-0.05 Mg alloy contained fully recrystallized fine equiaxed grains with a mean size of 0.40 μm (Fig. 4c), whereas a ternary Zn-0.05Mg-0.1Mn alloy had a greater grain size of 0.70 μm (Fig. 4d). Their tensile properties are tabulated in Table 5. In another study, Zn–Mg alloys containing various concentration of Mg (1.0, 1.5, 3.0 wt%) were investigated and reported to show a simultaneous increase in the hardness of the Zn matrices by increasing the Mg concentration which was due to formation of brittle Mg_2_Zn_11_ intermetallic particles in these alloys [94].

**Fig. 5:**
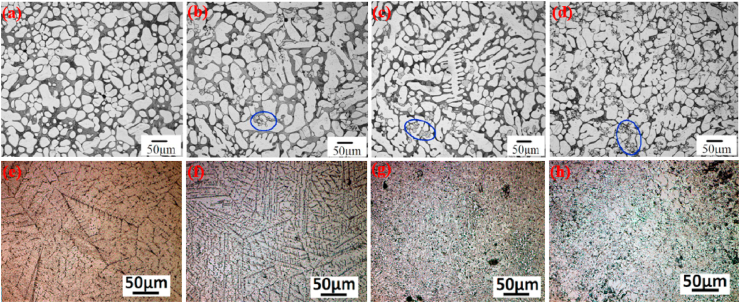
Optical micrographs of Zn–1Mg-xZr and Zn-xCa alloys: (a) Zn–1Mg, (b) Zn–1Mg-0.1Zr, (c) Zn–1Mg-0.25Zr, (d) Zn–1Mg-0.4Zr, (e) Zn-0.5Ca, (f) Zn–1Ca, (g) Zn–2Ca, and (h) Zn–3Ca. (Reproduced with permission from Ref. [166,181]).

Yang et al. [180] studied the microstructure and mechanical properties of as-extruded Zn-xMg-0.1Ca (x = 0.5, 1.0, 1.5) alloys and the mechanical properties obtained are summarized in Table 5. In another study, the effects of Zr (0–0.4 wt%) addition on the microstructure and mechanical properties of as-cast and extruded Zn–1Mg alloys were evaluated by Li et al. [166] and the as-cast microstructures obtained are shown in Fig. 5a–d. They reported that the microstructure of the Zn–Mg alloys consisted of Zn-rich dendrites and a lamellar eutectic Zn + Mg_2_Zn_11_ mixture (Fig. 5a), while after the addition of Zr, a few bar-like intermetallic phases of Zn_22_Zr were observed in the ternary alloys (Fig. 5b–d). Zou et al. [181] investigated binary Zn-xCa (x = 0.5, 1, 2, 3) alloys and their as-cast microstructures are presented in Fig. 5e–h. They reported that the addition of Ca notably increased the volume fraction of the second phase (CaZn_13_), and the morphology clearly changed to coarser ellipses from thin dendrites, as shown in Fig. 5h.

The effects of other nutrient elements (Ca and Sr) on the microstructure and mechanical properties of hypoeutectic Zn–Mg alloys have also been reported in recent studies [55,135,180]. For example, Liu et al. [135] observed the inhomogeneous precipitation of Mg_2_Zn_11_ and CaZn_13_ phases in as-cast Zn-1.5Mg-0.1Ca alloys, whereas the formation and uniform distribution of Mg_2_Zn_11_ and SrZn_13_ phases in as-cast Zn-1.5Mg-0.1Sr alloys led to grain refinement in these alloys. Their study revealed that lower concentrations of Ca and Sr (0.1 wt%) significantly increased the σ_TYS_ and σ_UTS_ of the Zn-1.5 Mg alloy; however, the ε was measured at only 2% for these alloys. Yang et al. [180] investigated the mechanical properties of an extruded Zn-xMg-0.1Ca alloy containing various Mg concentrations (0.5, 1.0 and 1.5 wt%). An increase in Mg concentration in this alloy caused a gradual increase in the content of a hard Mg_2_Zn_11_ intermetallic phase, which resulted in increases in hardness, σ_TYS_, and σ_UTS_. According to the phase diagrams for Zn–Ca and Zn–Sr, Ca and Sr are insoluble in Zn; consequently, even minor additions of Sr and Ca to Zn create intermetallic compounds such as SrZn_13_ and CaZn_13_. The effects of the addition of various alloying elements and manufacturing techniques on the mechanical properties of Zn alloys are summarized in Table 5. Based on data presented in Table 5, it can be concluded that alloying with Mg has the highest effect on enhancing the σ_UTS_, while Ca impacted mostly on ε. Moreover, among all the alloys, only four of the binary alloys, e.g., Zn-0.08 Mg, Zn-0.4 Mg, Zn–1Mg and Zn-1.2 Mg and one ternary alloy (Zn-0.05Mg-0.5Cu) have exhibited the mechanical benchmark values for vascular implant materials. Hence, from viewpoint of mechanical properties, till now the Zn-xMg (0.1 ≤ x ≤ 1.2) binary alloys are the best candidates for biodegradable implant applications. However, some ternary alloys, namely Zn–Mg–Ca/Sr have also shown a promising combination of σ_UTS_ and ε.

### 3.2 Zn alloys containing crucial elements

**Fig. 6:**
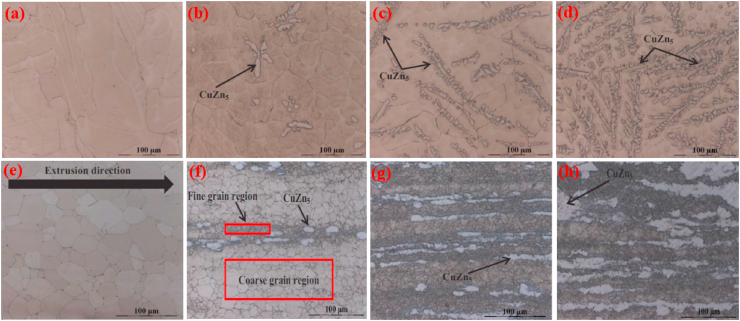
Optical micrographs of Zn-xMg alloys: (a) as-cast Zn–1Cu, (b) as-cast Zn–2Cu; (c) as-cast Zn–3Cu, (d) as-cast Zn–4Cu alloys, (e) hot-extruded Zn–1Cu, (f) hot-extruded Zn–2Cu, (g) hot-extruded Zn–3Cu, and (h) hot-extruded Zn–4Cu alloys. (Reproduced with permission from Ref. [162]).

Bone health is positively influenced by certain elements (Cu, Mn and Fe) known as crucial elements in the human body. The scarcity of these elements abates the increase of bone mass in childhood and/or in adolescence and speeds up bone loss after menopause or in old age. The deterioration of bone quality increases the risk of fractures [182,183]. However, this class of Zn alloys contains additional elements that are crucially required for metabolism, e.g., Cu, Fe, and Mn. The addition of these alloying element, particularly, Cu and Mn can improve the mechanical properties, not only strength, but also elongation. Cu is an essential trace element required for bone growth and connectivity, and enhances the proliferation of vascular endothelial cells, and revascularization [184,185]. In addition, Cu deficiency leads to normocytic cholesterol metabolism and neutropenia [186]. Based on the Zn–Cu phase diagram, the highest solubility of Cu in Zn is almost 2.75 wt% at 425 °C [89]. Tang et al. [162] studied a series of binary Zn-xCu alloys (x = 1, 2, 3 and 4 wt%) via casting and HE methods for cardiovascular implant applications and the microstructures obtained are illustrated in Fig. 6. Microstructural analysis revealed that the as-cast alloys were composed of a dendritic second phase of CuZn_5_ within the primary Zn matrix, and an increase in the volume fraction of the dendritic CuZn_5_ phase was observed at higher concentrations of Cu in Zn, as shown in Fig. 6a–d. HE of the alloys resulted in grain refinement (Fig. 6e–h), leading to improved mechanical properties (Table 5). In another study, the same research group reported that a small amount of Mg added to a binary Zn–Cu alloy resulted in enhanced mechanical properties due to the emergence of an Mg_2_Zn_11_ intermetallic phase [163]. Furthermore, the σ_TYS_ and σ_UTS_ increased, respectively, from 214 to 250 MPa and from 427 to 440 MPa by adding just 1.0 wt% Mg to the Zn-3.0Cu alloy; nonetheless, the ε decreased from 47% to 1% due to the presence of a hard-intermetallic phase.

**Fig. 7:**
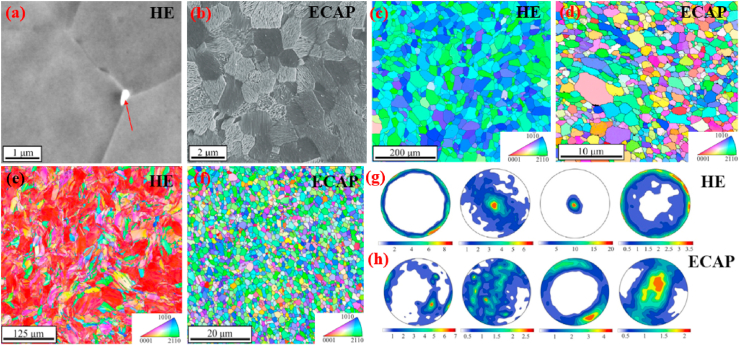
Microstructures of (a) HE and (b) ECAP Zn-0.49Cu alloy, EBSD-IPF maps of (a) HE and (b) ECAP Zn-0.49Cu alloy, EBSD microstructures after tensile deformation for (e) HE and (f) ECAP Zn-0.49Cu alloy, and pole figures of (g) HE and (h) ECAP Zn-0.49Cu alloy. (Reproduced with permission from Ref. [170]).

Another study was carried out by Yue et al. [105] to evaluate the influence of Fe additions (0.5 and 1.0 wt%) to as-cast and extruded binary Zn–Cu alloys. The microstructure of the as-cast Zn–3Cu alloy contained a CuZn_5_ secondary phase embedded in the Zn matrix, whereas the Zn–3Cu-0.5Fe and Zn–3Cu-1.0Fe alloys contained an additional coarse secondary phase of FeZn_13_ due to the Fe inclusion. The effects of Fe addition on the mechanical properties of these alloys are listed in Table 5. More recently, Bednarczyk et al. [170] fabricated a Zn-0.49Cu alloy using indirect HE (at 300 °C) and ECAP techniques, and the corresponding microstructures and EBSD-IPF (inverse pole figure) maps are shown in Fig. 7. The microstructures of both the HE and ECAP alloys consisted of equiaxed recrystallized grains and large amounts of a CuZn_5_ second phase were detected on grain boundaries, indicated by red arrow (Fig. 7a and b). The addition of low concentrations of Cu to Zn produced higher grain refinement in the ECAP alloy, with a grain size of ~2.2 μm, than in the HE alloy, with a grain size of ⁓37.8 μm (Fig. 7c and d). However, this grain refinement did not significantly enhance the mechanical strength but resulted in a notable increase in ε from 27% to 345%.

EBSD microstructural analysis after tensile testing for both processing techniques is shown in Fig. 7e and f, which indicate that all of the primary grains were deformed by twinning in HE, while twinning was not observed after ECAP; consequently, deformation happened by slip and non-slip deformation modes. Further, textural analysis revealed a typical Zn fiber texture for HE in which the grains were non-preferably oriented toward basal slip (Fig. 7g). In contrast, a distinct texture was observed after ECAP, where the orientation of crystallites preferred low-stress basal slip (Fig. 7h).

**Fig. 8:**
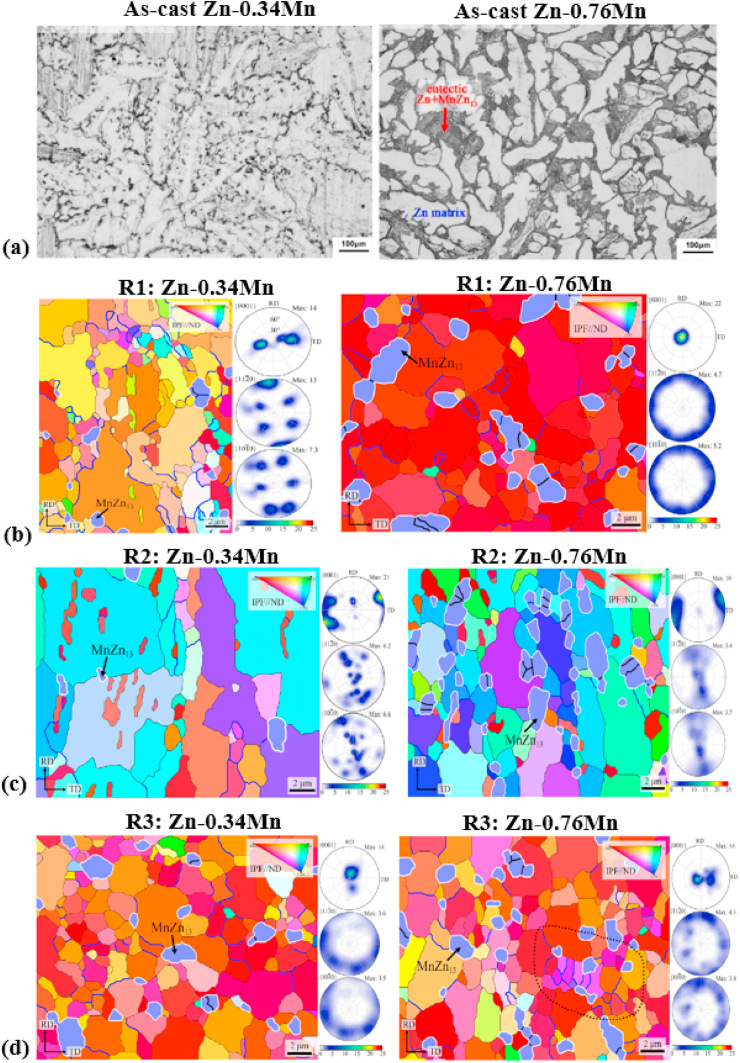
(a) Optical micrographs of as cast Zn-0.34Mn and Zn-0.76Mn alloys, and EBSD microstructures of Zn-0.34Mn and Zn-0.76Mn alloys: (b) R1, 83.3% hot-rolling; (c) R2, 83.3% hot-rolling + 84.0% cold-rolling; (d) R3, 83.3% hot-rolling, annealing (200 °C × 2 h, air cooling), and 84.0% cold-rolling. (Reproduced with permission from Refs. [189]).

Another essential trace element for various enzymes and the human immune system is Mn [187], which has low solubility in Zn, approximately 0.8 wt% at a temperature of 416 °C [176]. At this temperature, a solid solution of Zn and an intermetallic phase MnZn_13_ coexist owing to the eutectic reaction. Sun et al. [188] studied the microstructure and mechanical properties of as-extruded Zn–Mn alloys containing different Mn concentrations (0.2, 0.4, and 0.6 wt%) and the results revealed that Zn-0.2Mn alloy was comprised of only a Zn-rich phase, while a secondary phase of MnZn_13_ was observed in the other two Zn–Mn alloys. The addition of Mn in Zn alloys significantly enhanced the ε from 48% to 71%. However, the σ_UTS_ of the alloys was slightly decreased, with increasing content of Mn. In another study, Shi et al. [189] evaluated as-cast Zn-0.34Mn and Zn-0.76Mn alloys using three processing routes: (R1) 83.3% HR (R2) 83.3% HR + 84.0% cold-rolling; and (R3) 83.3% HR + annealing (200 °C × 2 h, air cooling) + 84.0% cold-rolling. Optical micrographs and EBSD maps of these alloys are presented in Fig. 8. The microstructure of the as-cast Zn-0.34Mn alloy included coarse dendrites, but the addition of 0.76 wt% Mn caused grain refinement along with the formation of a second phase, MnZn_13_ (Fig. 8a). The EBSD-measured microstructures shown in Fig. 8b reveal that R1-processed Zn-0.76Mn alloy contained more equiaxed grains (91.1%) than the Zn-0.34Mn alloy (61.2%), signifying a higher degree of recrystallization. Also, the Zn-0.34Mn alloy showed a typical texture featuring a pair of poles almost 45° from the normal direction with a highest pole density of 14. Conversely, the Zn-0.76Mn alloy showed a texture with one pole centered at normal direction with a highest pole density of 22. The R2-processed Zn-0.76Mn alloy had smaller grains (2.1 ± 1.3 μm) than the Zn-0.34Mn alloy (3.0 ± 2.6 μm), revealing that grain growth in the former alloy was restrained by more uniformly distributed MnZn_13_ particles (Fig. 8c). The R3-processed alloys showed similar equiaxed grains, e.g., 88.7% and 84.0% for the Zn-0.34Mn and Zn-0.76Mn alloys, respectively, as depicted in Fig. 8d. However, the mechanical properties of both as-cast alloys exhibited a very brittle nature with very low elongation (<1%), as summarized in Table 5. The σ_TYS_, σ_UTS_, and E were notably improved by applying HR (R1) to the Zn-0.34Mn alloy; however, in R2, RT work-softening occurred instead of work-hardening, so that σ_TYS_ and σ_UTS_ declined, but elongation doubled (to 75.7%), as shown in Table 5. Analogous RT work-softening was observed with R2 for the Zn-0.76Mn alloy. Interestingly, the usage of R3 on the Zn-0.34Mn and Zn-0.76Mn alloys gave them extremely high elongation, e.g., 88.8% and 94.0% at RT, respectively (Table 5). The microstructures and mechanical properties of PM-processed Zn–4Mn and Zn–24Mn alloys were investigated by Bagha et al. [139]. The microstructures of these alloys contained nano-sized crystallites (>40 nm) and the secondary phases of MnZn_3_ and MnZn_13_, which enhanced the compression strength, elastic modulus and micro-hardness of these alloys. Recently, Guo et al. [190] reported that a multi-pass HE-processed Zn-0.5Mn alloy contained ultra-fine Zn grains (with 0.35 μm grain size) and a second phase of MnZn_13_ (with 0.07 μm grain size), which caused grain refinement and hence superplasticity, i.e., ε = 236.2% at RT. In a very recent report, Shi et al. [191] studied the influence of Ag, Cu, and Ca on as-cast and extruded Zn-0.8Mn alloys. Low additions (0.4 wt%) of Ag, Cu, and Ca to Zn-0.8Mn in an as-cast state made the ternary alloys even more brittle, while application of HE not only improved elongation but also increased the strength of the ternary alloys significantly (Table 5). In another study, Shi et al. [192] investigated the mechanical properties of biodegradable Zn-0.3Fe alloy fabricated via a newly developed bottom circulating water-cooled casting (BCWC) method. The BCWC method significantly refined the secondary phase of FeZn_13_ particles in Zn-0.3Fe. As a result, the ultimate tensile strength (σ_UTS_) of Zn-0.3Fe alloy increased by 62% than that of the same alloy fabricated via conventional casting. The microstructural evolution and mechanical properties of the Zn-0.3Fe alloy are summarized in Table 5.

It is observed from Table 5 that among all alloys alloyed with crucial elements, only two alloys (Zn–2Cu and Zn–4Cu) exceeded the mechanical benchmark values for vascular implant materials. However, the Mn and Cu has the greatest impact on improving ε values for the biodegradable Zn-based alloys.

### 3.3 Zn alloys containing other elements

In this group, Zn based alloys cover a wide range of alloying elements, such as, zirconium (Zr), lithium (Li), Ti, germanium (Ge), aluminum (Al), and silver (Ag). Among these elements, the addition of Li into pure Zn not only enhances the strength, but also elongation of the Zn-alloys.

**Fig. 9:**
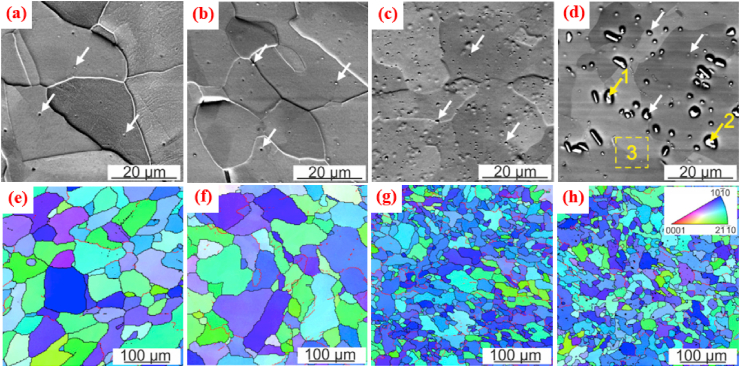
Optical micrographs of as-extruded (a) Zn-0.01Zr, (b) Zn-0.02Zr, (c) Zn-0.05Zr, and (d) Zn-0.1Zr alloys. EBSD maps of as-extruded (e) Zn-0.01Zr, (f) Zn-0.02Zr, (g) Zn-0.05Zr, and (h) Zn-0.1Zr alloys. (Reproduced with permission from Ref. [194]).

It is known from the Zn–Zr phase diagram [193] that Zr is virtually insoluble in Zn, i.e., the solid solubility of Zr in Zn is below 0.014 wt% at 400 °C, so a tiny addition of Zr in Zn will generate a Zr-rich intermetallic phase. Wątroba et al. [194] studied Zn–Zr alloys with Zr content of 0.01, 0.02, 0.05, and 0.1 wt% via die casting and HE, and their optical and EBSD microstructures are presented in Fig. 9. The microstructural analysis (via BSE imaging) indicated that with increasing wt.% of Zr, the particle size of Zr and volume fraction of the intermetallic phase (Zn_22_Zr) increased, as shown by white arrows in Fig. 9a–d. Also, the IPF maps displayed in Fig. 9e–h and grain size distribution of all alloys confirmed grain refinement from 210 to 42 μm due to the increased Zr content. The mechanical property analysis concluded that low additions of Zr gave the alloy similar properties to brittle Zn. At the same time, higher content (0.05 and 0.1 wt%) of Zr in Zn notably enhanced both the strength and ductility of the alloys. However, the most promising Zn–Zr alloy is the HE Zn-0.05Zr alloy, with σ_TYS_, σ_UTS_, and E of 104 MPa, 157 MPa, and 22%, respectively. Recently, another element, Ge, was incorporated into Zn to produce a Zn-5Ge alloy, by Tong et al. [168], via casting and HR techniques, and the as-cast microstructure exhibited an α-Zn phase with a eutectic Ge phase. In the case of the HR Zn-5Ge alloy, the grains were found to be elongated in the deformation direction; the Ge phase was also notably refined. HR significantly increased the σ_TYS_, σ_UTS_, E, and hardness values of the Zn-5Ge alloys, as shown in Table 5.

**Fig. 10:**
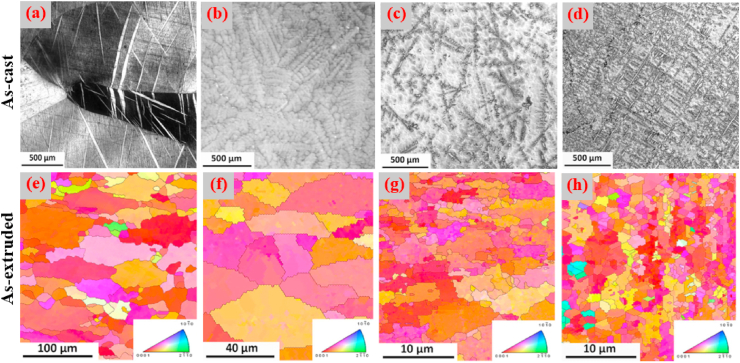
Optical micrographs of as-cast (a) pure Zn, (b) Zn-2.5Ag, (c) Zn-5.0Ag, and (d) Zn-7.0Ag alloys. EBSD maps of the extruded (e) pure Zn, (f) Zn-2.5Ag, (g) Zn-5.0Ag, and (h) Zn-7.0Ag alloys. (Reproduced with permission from Ref. [102]).

Another element alloyed with Zn is Ag, which has been used for decades to treat burns and in wound healing and is now used in several clinical applications [195,196]. Materials containing Ag were effectively utilized as dental implants and in some cases as biomaterial coatings [197,198]. Many studies have reported that Ag ions or nanoparticles can kill some bacteria which cling to the implant surface or prevent them from attaching to it [199]. Therefore, the addition of Ag to Zn or Zn-based alloys would benefit cardiovascular stent applications. However, as per the Zn–Ag phase diagram [176], Ag shows a maximal solubility of roughly 6 wt% in Zn at 431 °C, where solidification occurs by a peritectic reaction in which β-AgZn_3_ and the liquid change into an η-Zn solid solution. Binary Zn-xAg alloys with x = 2.5, 5.0, 7.0 wt% were studied by Sikora-Jasinska et al. [102] and their optical and EBSD microstructures are shown in Fig. 10. The optical micrographs in Fig. 10a reveal that as-cast pure Zn is composed of very coarse grains (<1 mm), but the addition of 2.5 wt% Ag to Zn causes a notably refined dendritic structure (Fig. 10b). Further, alloys with larger wt.% of Ag exhibit an elementary η-Zn phase and correspondingly higher volume fraction of ε-AgZn_3_ dendrites (Fig. 10c and d). It was found from the EBSD maps and IPFs (shown in Fig. 10e–h) that observable grain refinement occurred because of the Ag content and HE, and consequently σ_TYS_ and σ_UTS_ successively increased, respectively, from, 147–236 MPa and from 203 to 287 MPa for Zn-2.5Ag and Zn-7.0Ag. Nonetheless, the inclusion of Ag with Zn reduced the ε of all Zn–Ag alloys, but interestingly the values of ε remained almost the same at higher wt.% of Ag and the remaining values of E were still fairly adequate (32–36%) for various implant applications. In another study, Li et al. [167] reported that a thermal treatment of Zn-4.0Ag refined the microstructure and so enhanced the mechanical properties. Porous Zn-1.0Ag and Zn-3.5Ag alloy scaffolds with porosity of almost 59% were fabricated by Xie et al. [200] via the air-pressure infiltration technique and they reported that with increasing Ag content, the grain size of the alloys reduced gradually to 40 μm, which resulted from the increase in the mechanical properties of the Zn–Ag scaffolds. Recently, Bednarczyk et al. [171] studied an ECAP-processed Zn-0.8Ag alloy with surprising elongation (<650%).

**Fig. 11:**
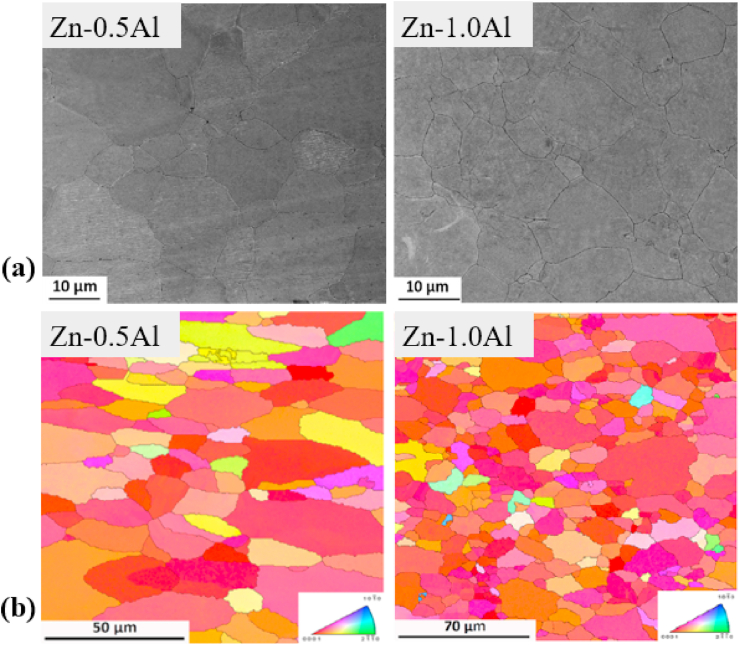
(a) Optical micrographs and (b) EBSD maps of the Zn-0.5Al and Zn-1.0Al alloys. (Reproduced with permission from Ref. [178]).

Aluminum is another element for alloying with Zn and Zn-based alloys and has the highest solubility (1 wt%) in Zn at 382 °C. However, the neurotoxicity of Al and its susceptibility for causing Alzheimer's disease have been reported in the literature [201,202]. Therefore, the content of Al in biodegradable Zn alloys should be limited. Nevertheless, Al in its low concentrations has been extensively used as an alloying element in several biomedical implant materials which have served the biomedical device industry for the last few decades [136,203,204]. A eutectic reaction at 382 °C produces a mixture of β-Zn (HCP crystals) and α′-Al (CFC crystals), and below 275 °C a monotectoid reaction occurs to transform α′-Al into α-Al [176]. Low Al content (0.5 and 1.0 wt%) Zn–Al alloys were studied by Mostaed et al. [178] and the SEM microstructures and EBSD maps obtained are shown in Fig. 11. SEM images of both alloys (Fig. 11a) confirm the absence of secondary phases, due to the higher solubility of Al in Zn. An equiaxial grain was also observed along longitudinal cross-sections, as presented in Fig. 11b, ensuring notable grain refinement. Also, the second phase was absent, indicating good solubility of Al in α-Zn. The addition of Al notably enhanced the strength and elongation; however, σ_TYS_ and σ_UTS_ were still below the benchmark values for implant applications. In another study, Demirtas et al. [172] reported that a fine-grained matrix and ultrafine-grained Al precipitates generate extremely high elongation, such as 1000% in an ECAP-processed Zn-0.3Al alloy at RT. Recently, Bowen et al. [146] explored a series of Zn-xAl alloys (x = 1, 3, 5 wt%) using HR for implant applications and obtained values for σ_UTS_ and elongation higher than 308 MPa and 31%, respectively, for Zn–3Al and Zn–5Al alloys, which are very close to the benchmarks; however, the σ_UTS_ value for Zn–1Al was still below the benchmark. The effects of several wt.% (0.1, 0.3, 0.5) of Mg addition to Zn-0.5Al were investigated by Bakhsheshi-Rad et al. [136] and they reported that Zn-0.5Al-xMg alloys were composed of α-Zn and lamellae of Mg_2_(Zn, Al)_11_. Also, the mechanical properties, i.e., σ_UTS_, E and HV, of the Zn-0.5Al alloys were consistently enriched by using Mg content, yet they obtained values were far below the benchmark values for stent materials. The same research group also reported that adding Bi to Zn-0.5Al-0.5 Mg alloys aided the formation of an α-Mg_3_Bi_2_ phase, which enhanced mechanical properties [205].

**Fig. 12:**
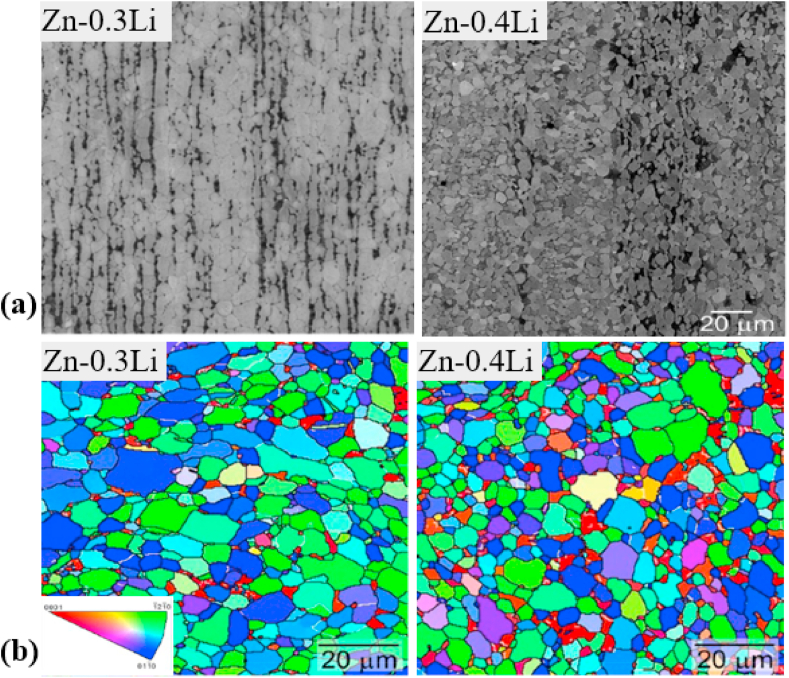
(a) SEM micrographs and (b) EBSD maps of the as-extruded Zn-0.3Li and Zn-0.4Li alloys. (Reproduced with permission from Ref. [211]).

An overdose of Li poses various potential health risks, including congenital disabilities, bipolar disorder, etc. [206,207], but it was reported that low content of Li is useful in the treatment of brain injury, stroke, spinal cord injury, and Parkinson's disease [208]. Moreover, the addition of Li to Mg markedly enhanced the ductility of Mg, and thus Mg–Li alloys have more significant potential compared to Mg alloys to satisfy the demand for cardiovascular stents [209]. However, according to the phase diagram for Zn–Li [210], the highest solubility of Li in Zn is ~0.12 wt% at 403 °C. A eutectic reaction at 403 °C transforms the liquid phase into Zn and β-LiZn_4_, and then below 65 °C its further changes into α-LiZn_4_. Zhu et al. [211] studied as-extruded Zn-xLi (x = 0.3, 0.4) alloys, and the SEM microstructures and EBSD maps obtained are shown in Fig. 12. They reported that both alloys contained equiaxed grains and strings of an intermetallic phase of LiZn_4_, as shown in Fig. 12a, and the average grain sizes determined from EBSD maps (Fig. 12b) were approximately 11 μm and 6 μm, respectively. Li et al. [212] fabricated the Zn-(0.1–1.4)Li alloys to systematically investigate the impact of Li on the microstructure and mechanical properties of the alloys. The Zn–Li alloys contained mainly α-Zn and β-LiZn_4_ phases. The strength of the Zn–Li alloys increased at least 3 times due to the formation of a dense β-LiZn_4_/α-Zn lamellar structure as summarized in Table 5. A series of Zn–Li alloys with varying Li content (0.2, 0.4, 0.7 at.%) were also investigated by Zhao et al. [103]. The as-cast Zn-0.2Li alloy contained a small amount of Zn + LiZn_4_ phase in the α-Zn matrix which was homogenously dispersed within the matrix. However, Zn alloys containing higher Li content exhibited randomly oriented lamellar Zn + LiZn_4_ phase along the grain boundaries. On the contrary, acute rolling texture with finer dendrites and grains were observed in the hot rolled Zn-0.4Li and Zn-0.7Li alloys, whilst equiaxed grains were observed in Zn-0.2Li alloy owing to dynamic recrystallization (DRC). The increase in Li content from 0.2 to 0.7 at.% simultaneously improved the σ_YS_ and σ_UTS_ from 240 to 480 MPa and 360–560 MPa, respectively, yet their ductility was found relatively lower than the benchmark value of elongation for stent materials (20%). In a recent study, excellent mechanical strength and ductility of hot extruded Zn-xLi (x = 0.1, 0.4, 0.8) alloys with addition of Mn from 0.1 to 0.8 wt% were reported by Yang et al. [213]. Their reported values of σ_TYS_, σ_UTS_ and ε for all the binary and ternary alloys were well above the benchmark values for any vascular stent materials as shown in Table 5.

Table 5 also systematically summarizes the effects of these alloying elements and fabrication techniques on the formation of different phases and the subsequent mechanical properties of Zn-based alloys.

Form Table 5, it can be summarized that the only Zn-xLi-yMn (x, y = 0.1–0.8 wt%) are the best candidates for next generation biodegradable implant applications. However, other binary systems like Zn–Ag and Zn–Ti, as well as systems like Zn–Ag–Mn have also revealed an optimistic combination of σ_UTS_ and ε.

## 4 Tribological properties of Zn-alloys

Tribology is the science of wear, friction and lubrication, and encompasses how interacting surfaces and other tribo-elements behave in relative motion in natural and artificial systems. The wear resistance is critical for Zn alloys due to the particular applications, in fashion, decorative, automotive, and biomedical industry. In addition, Zn alloys have been used for bearing production to replace existing Cu-based bearings due to the good castability and unique combination of properties. Consequently, sliding wear behavior of Zn–Al alloys has been extensively investigated via standard pin-on-disk [242,243] or block-on-disk (ring) wear tests [244,245] under both dry and lubricated conditions. Since wear resistance is not an intrinsic property of the material, it relies on the specific tribological system and the testing conditions. As a consequence, a comparison of the wear behavior of alloys tested under different conditions (i.e., different applied load, sliding distance, with or without lubrication, etc.) is not reputable. However, microstructural features govern the resultant tribological performance of Zn–Al alloys [[246], [247], [248], [249], [250]]. For example, tribological performance of lower Al content hypo-eutectic Zamak 2 (Zn–4Al–3Cu) and Zamak 3 (Zn–4Al-0.1Cu) alloys were investigated in comparison with Zn alloys containing higher levels of Cu and/or Al [246,247]. Generally, the low hardness of Zamak 3 alloy (due to the primary Zn-rich α-phase) resulted in higher friction coefficient (COF) and wear rate than the other alloys when tested against steel counterpart in dry conditions [247]. Conversely, Zamak 2 alloy exhibited higher hardness due to the higher Cu content, but it was reported displaying worse wear resistance compared to alloys with higher Al content alloys like Zn–15Al–1Cu (ZEP) and Zn–27Al (ZA27). This is possibly owing to a limited oxide formation on the wear track, whereas the oxide formed on the wear track of ZEP and ZA27 alloys protected their surfaces from further wear. However, Zamak 2 also underwent a noticeable oxidation on the wear track by increasing the sliding distance [246]. In all the alloys, scratches aligned with the sliding direction were observed which were formed due to abrasive wear damage. Ares et al. [248] evaluated the wear resistance of hypoeutectic Zn-xAl (x = 1–4 wt%) alloys fabricated by transient directional solidification (TDS) and reported that under the same wear conditions, the wear rate of the equiaxed region was lower than that of the columnar and transition regions, and for each alloy concentration, the wear resistance increased from the columnar to the equiaxed structure. The improvement in wear resistance with increasing Al content in pure Zn at high loads (40–100 N) was attributed to the specific microstructural evolution [249]. The role of Ag was studied for gravity casting Zn–12Al alloy by Şevik [250] and the modified alloys (Zn–Al–Ag) exhibited higher wear resistance compared to the base alloy of Zn–12Al. Both wear rate and COF for all applied loads were reduced with increasing Ag content. In another study, Turk et al. [242] investigated the wear behavior of Zn–8Al alloys containing various concentrations of lead (Pb), tin (Sn), and cadmium (Cd) at different sliding speeds and applied loads. Their results indicated that Pb and Cd elements enhanced the wear resistance, particularly at high loads (30–45 N) while alloys with Sn exhibited poor wear behavior in comparison with the base Zn–8Al alloy. Similar tribological behavior was reported by Savaskan et al. [251] in which addition of high content of Cu (up to 2 wt%) in Zn–27Al increased its wear resistance, while for a higher content no significant improvement in material performance can be observed.

**Fig. 13:**
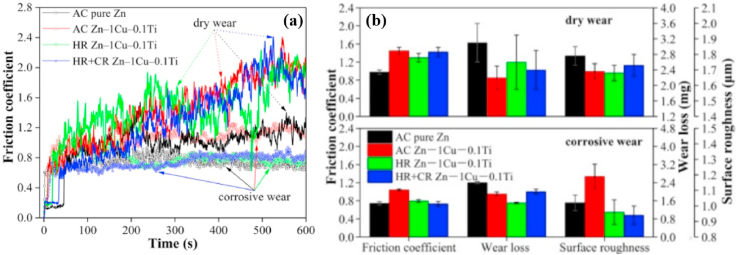
Friction behaviors of AC, HR, and HR + Cold rolled Zn–1Cu–0.1Ti and as-cast pure Zn: (a) friction coefficient curves in dry-sliding wear and corrosive wear testing in Hanks' solution and (b) friction coefficient, wear loss and surface roughness after wear testing. (Reproduced with permission from Ref. [152]).

The tribological behavior of other Zn-based alloys for biomedical applications are rarely reported in the literature. Recently, Lin et al. [152] reported the wear and friction behavior of as-cast, HR and cold rolled biodegradable Zn–1Cu-0.1Ti alloys with pure Zn as control and the results are shown in Fig. 13. The COF values as a function of wear time for all the samples are shown in Fig. 13a. It can be seen that the COF was relatively stable at the initial stage of wear under dry sliding, and then increased rapidly with sharp fluctuations with increasing sliding time. The COF, wear loss (WL), and surface roughness (SR) of all the samples after dry-sliding wear and corrosive wear testing in Hanks' solution is shown in Fig. 13b. The COF, WL and SR values are 0.741, 2.41 mg, and 1.02 μm for as-cast Zn and 1.039, 1.87 mg and 1.19 μm for as-cast Zn–Cu–Ti, respectively. The HR + cold rolling process significantly reduced the COF and SR from 1.039 to 0.731 and 1.19 to 0.94 μm, respectively, while increased the WL value from 1.87 to 2.02 mg. The overall results indicate that the ac-cast, HR, and HR + cold rolling processed Zn–1Cu–0.1Ti and as-cast pure Zn exhibited better wear performances in the lubricated environment of Hanks’ solution than in the dry-sliding wear conditions. The same research group in another study reported the friction and wear behaviors of the Zn–3Cu and Zn–3Cu–0.2Ti alloys [252]. With the addition of 0.2% Ti, the COF and WL of Zn–3Cu–0.2Ti revealed downward trends compared to their Zn–3Cu counterparts, indicating higher wear resistance of the Zn–3Cu–0.2Ti alloys than the Zn–3Cu under the same conditions.

## 5 Corrosion mechanisms and degradation behaviors of pure Zn and Zn-based alloys

Zn is commonly used as a corrosion-protection material in marine and industrial applications [253]. Metallic materials used in these industries are generally coated with Zn-based materials, which act as a sacrificial layer to protect against further corrosion of structural components. However, for biomedical applications, degradation of Zn and its alloys is mainly assessed via *in vitro* and *in vivo* studies. The *in vitro* corrosion behavior of Zn-based materials is commonly assessed by electrochemical and weight-loss tests. The electrochemical testing includes standard potentiodynamic polarization (PP) and electrochemical impedance spectroscopy (EIS) studies of these materials. *In vitro* degradation of Zn-based materials in various corrosion mediums has been investigated in recent years, including Hanks' Balanced Salt Solution (HBSS), phosphate-buffered saline (PBS) solution, simulated body fluid (SBF), and Ringer's Saline Solution (RSS) [148,254,255]. Genuine human plasma and blood were also used in a few studies [256,257]. The corrosion rate (C.R) of Zn-based materials can be evaluated using ASTM G59-97 [258]:

(1) $$C.R_{ele}=3.27\;\times \;10^{3}\frac{i_{corr}EW}{\rho }$$

where *i*_corr_ is the electrochemical corrosion density, EW is the equivalent weight (g/eq), and *ρ* is the density (g/cm^3^) of the Zn-based materials.

EIS generally employs AC and DC current polarization in the usual potentiodynamic test, which evaluates the resistance, impedance, and capacitance of materials immersed in various corrosion mediums. The corrosion current density is then evaluated using the Stern–Geary relationship [258]:

(2) $$i_{corr}=\;\frac{\beta_{a}\beta_{c}}{2.303R_{p}\left(\beta_{a}+\;\beta_{c}\right)}$$

where *R*_p_, *β*_a_, and *β*_c_ are the polarization resistance, anodic, and cathodic Tafel slopes, respectively.

The degradation behavior of Zn-based materials is also assessed by weight-loss measurement. These materials are generally immersed for a defined duration in various corrosion mediums under static conditions. The corrosion rates from immersion tests can be calculated using [259]:

(3) $$C.R_{imm}=\;\frac{KW}{At\rho }$$

where *W*, *A*, *t*, and *K* are the mass loss in grams, the surface area of the specimen in cm^2^, the immersion period in hours, and a constant, respectively.

However, irrespective of the corrosion mediums used in these studies, the corrosion mechanism of Zn is regulated by the following reactions [42,260]:

(4) Anodic reaction: Zn → Zn^2+^ + 2e^−^

(5) Cathodic reaction: 2H_2_O + O_2_ + 4e^−^ → 4OH^−^

(6) Overall reaction: 2Zn + 2H_2_O + O_2_ → 2Zn(OH)_2_ ↓

(7) Other reactions: Zn(OH)_2_ → ZnO + H_2_O

6Zn(OH)_2_ + Zn^2+^ + 2Cl^-^ →6Zn(OH)_2_·ZnCl_2_ (8)

4ZnO + 4H_2_O + Zn^2+^ + 2Cl^-^ → 4Zn(OH)_2_. ZnCl_2_ (9)

(10) Zn^2+^ + 2HPO_4_^−2^ + 2OH^−^ + 2H_2_O → Zn_3_(PO_4_)_2_·4(H_2_O)

**Fig. 14:**
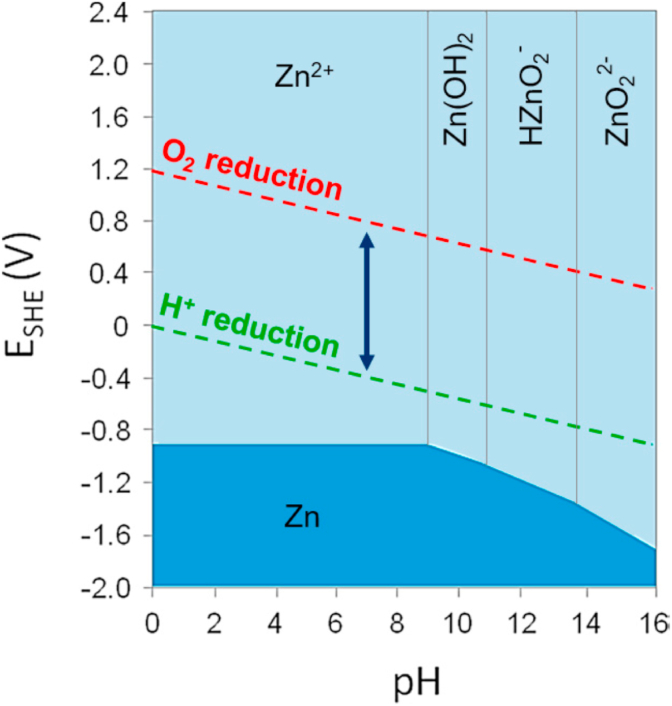
Pourbaix diagram of Zn. The blue arrow shows the range of biological standard reduction potentials at pH 7.4. (Reproduced with permission from Ref. [265]).

It is evident from these series of reactions that Zn doesn't release hydrogen gas during biodegradation like Mg, indicating one of the major benefits of Zn [88,178,261]. The corrosion by-products from the degradation of Zn contain its oxides and some other elements and compounds including phosphorus (P), Ca, chlorine (Cl), phosphates (PO_4_), and bicarbonates (HCO_3_) [78,226,236,[262], [263], [264]]. Apart from the chemical compositions of Zn and its alloys, the pH of the corrosion medium plays a critical role during their corrosion [265,266]. However, the overall corrosion rates of Zn-based materials are characterized by their lower cathodic reaction rates in pH values between 7 and 10, as shown in Fig. 14 [265].

The addition of alloying elements can regulate the corrosion mechanism and the formation of corrosion products in Zn-based materials. The corrosion behavior of Zn and its alloys is typically associated with the size, distribution, and volume fraction of the secondary phases, which become cathodic sites during their biodegradation in corrosive mediums.

### 5.1 In vitro degradation of Zn-based materials

The *in vitro* corrosion behavior of pure Zn in different corrosive mediums has been extensively investigated by researchers [85,256,[267], [268], [269]]. For example, the corrosion behavior of pure Zn in two corrosive solutions, PBS and RSS, and two natural body fluids, human plasma and whole blood, was first reported by Torne et al. [256] and their results indicated that corrosion rates decreased with immersion time for plasma and whole blood, while they increased during immersion in PBS and RSS. Liu et al. [71] evaluated and compared the *in vitro* corrosion behaviors of ultra-pure (UP) Zn and pure Mg plates and mini-tubes and reported that UP-Zn plates had lower corrosion rates than pure Mg, while Zn mini-tubes showed a higher corrosion rate than Zn plates. In another study, Chen et al. [262] compared the degradation behaviors of Zn with Mg and Fe in PBS solution, and reported that the open circuit potential and corrosion rates of Zn were in between those of Mg and Fe. The influence of diameter on the *in vitro* corrosion performance of as-extruded pure Zn wires in HBSS was also investigated by Guo et al. [268] and their results showed that during 30 d immersion, a 3 mm Zn wire exhibited much better corrosion resistance than a 0.3 mm Zn wire. Recently, *in vitro* degradation of pure Zn was investigated by Liu et al. [267] by immersing it for 4 weeks in bovine serum albumin (BSA). Their results indicated that the adsorption of BSA protected the substrate from dissolution on the first day, but chelation of BSA and Zn^2+^ increased the corrosion rates over 3–7 d immersion. After this period, the corrosion resistance of pure Zn was increased by the formation of a complex accumulation on the surface of the sample. Similarly, many studies have reported on the *in vitro* degradation behavior of Zn and its alloys in various corrosive mediums such as HBSD, PBS, SBF, artificial urine (AU), and artificial plasma (AP) at different immersion durations. These studies revealed that composition and fabrication techniques alter the degradation behavior of Zn-based materials. Results from these studies are summarized in Table 6.

**Table 6:** Effect of composition and fabrication processes on the degradation behavior of Zn-based alloys (average values are reported).

Composition (wt.%) and manufacturing process	Corrosion medium	Immersion time (day)	**E**_**corr**_**(V)**	**I**_**corr**_**(μA/cm**^**2**^**)**	C.R (mm/y)		Ref.
					C.R _imm_	C.R_ele_	
**Pure Zn**							
**Pure-Zn**	Saline	14	−1.25	16.73	–	0.063	[269]
	RSS	3	−1.049	–	0.10	0.094	[256]
	PBS	3	−0.994	–	0.03	0.021	
	WB	3	−1.130	–	0.11	0.104	
	HP	3	−1.094	–	0.19	0.179	
	SBF	14	−1.08	11.90	0.03	0.18	[264]
**Zn (foil)**	AP	28	−0.97	0.76	0.016	0.011	[267]
**Zn**^**Cast**^	HBSS	30	−1.170	37		0.56	[274]
**Zn**^**HE**^		30	−1.016	30.361	0.027	0.909	[268]
**Zn**^**HE + DW**^		30	−1.009	14.045	0.035	0.421	
**Zn (plate)**^**HE + DW**^		30	–	–	0.013	–	[71]
**Zn (tube)**^**HE + DW**^		30	–	–	0.037	–	
**Zn Alloyed with nutrient elements (Ca, Mg and Sr)**							
**Zn**^**HE**^	SBF	14	−0.914	44.0	0.15	0.653	[148]
**Zn-0.005Mg**^**HE**^		14	−0.938	49.1	0.15	0.728	
**Zn**^**HE**^	HBSS	15	−1.117	2.81	0.09	0.042	[255]
**Zn-0.02Mg**^**HE**^		15	−1.113	6.19	0.21	0.093	
**Zn**^**HE**^	AU	–	−1.11	58	–	0.87	[273]
**Zn-0.5Mg**^**HE**^		–	−1.18	92	–	1.39	
**Zn-1.0Mg**^**HE**^		–	−1.17	99	–	1.50	
**Zn**^**SLM**^	SBF	28	−0.87	9.24	0.18	–	[142]
**Zn-1Mg**^**SLM**^		28	−0.91	5.86	0.14	–	
**Zn-2Mg**^**SLM**^		28	−0.88	4.63	0.13	–	
**Zn-3Mg**^**SLM**^		28	−0.82	3.62	0.10	–	
**Zn-4Mg**^**SLM**^		28	−0.84	3.71	0.11	–	
**Zn-1Mg**^**Cast**^		30 min.	−1.14	28.47	0.43	–	[275]
**Zn-1Mg**^**Cast**^		7	–	–	0.28	–	[104]
**Zn-1Mg**^**Ext**^		7	–	–	0.12	–	
**Zn-1Mg**^**PM**^	Ringer's	2	−1.224	7.244	0.208	–	[138]
**Zn-25Mg**^**PM**^		2	−1.323	12.99	0.374	–	
**Zn**^**Cast + Hom**^	SBF	14	−0.89	9.7	0.073	–	[94]
**Zn-1Mg**^**Cast + Hom**^		14	−0.98	1.2	0.083	–	
**Zn-1.5Mg**^**Cast + Hom**^		14	−0.93	8.8	0.075	–	
**Zn-3.0 Mg**^**Cast + Hom**^		14	−0.93	7.4	0.081	–	
**Zn**^**Cast**^	SBF	14	–	–	–	0.064	[214]
**Zn-1Mg**^**Cast**^		14	–	–	–	0.053	
**Zn-1.5Mg**^**Cast**^		14	–	–	–	0.058	
**Zn-3.0 Mg**^**Cast**^		14	–	–	–	0.052	
**Zn**^**Cast**^	HBSS	14	−0.99	9.20	–	0.137	[178]
**Zn-0.15Mg**^**Cast**^		14	−1.03	11.52	–	0.172	
**Zn-0.5Mg**^**Cast**^		14	−1.05	11.73	–	0.175	
**Zn-1.0Mg**^**Cast**^		14	−1.07	11.88	–	0.177	
**Zn-3.0Mg**^**Cast**^		14	−0.98	9.01	–	0.135	
**Zn**^**HE**^		14	−0.98	8.98	0.074	0.134	
**Zn-0.15Mg**^**HE**^		14	−1.01	10.98	0.079	0.164	
**Zn-0.5Mg**^**HE**^		14	−1.02	11.01	0.081	0.164	
**Zn-1.0Mg**^**HE**^		14	−1.05	11.32	0.083	0.169	
**Zn-3.0Mg**^**HE**^		14	−0.92	8.60	0.076	0.128	
**Zn-1.2Mg**^**Cast**^	HBSS	90	−1.18	7.68	0.07	0.12	[220]
**Zn-1.2Mg**^**HE**^		90	−1.20	12.38	0.09	0.19	
**Zn-0.8Mg**^**HE**^	MEM	1	–	–	0.071	–	[164]
**Zn**^**Cast**^	SBF	6	−1.050	2.5	0.038	–	[276]
**Zn-1.0Mg**^**Cast**^		6	−1.076	3.6	0.052	–	
**Zn-2.0Mg**^**Cast**^		6	−1.056	1.4	0.020	–	
**Zn-1.5Mg**^**HE**^		3	–	–	0.101	–	[263]
**Zn-3.0Mg**^**Cast**^		21	–	–	0.21	–	[222]
**Zn-3.0Mg**^**Hom**^		21	–	–	0.13	–	
		21	−0.902	3.4	0.25	0.30	
**Zn-3.0Mg**^**1-ECAP**^		21	−0.865	2.7	0.18	0.24	[160]
**Zn-3.0Mg**^**2-ECAP**^		21	−0.893	3.2	0.19	0.28	
**Zn-2.2Mg**^**DSC**^	NaCl	–	−0.938	0.17	–	5.88 cm^2^/μA	[137]
**Zn-3.15Mg**^**DSC**^		–	−0.918	0.18	–	5.55 cm^2^/μA	
**Zn-0.5Mg-0.1Ca**^**HE**^	PBS	–	−1.25	2.42	–	0.028	[180]
**Zn-1.0Mg-0.1Ca**^**HE**^		–	−1.20	1.82	–	0.021	
**Zn-1.5Mg-0.1Ca**^**HE**^		–	−1.18	2.08	–	0.024	
**Zn**^**HR**^	HBSS	56	−0.988	9.07	0.078	0.135	[147]
**Zn-1.0Mg**^**HR**^		56	−0.999	9.94	0.086	0.149	
**Zn-1.0Mg**^**Cast**^	PBS	30	−1.09	0.74	–	0.012	[107]
**Zn-1.0Mg-0.5Ca**^**Cast**^		30	−1.07	4.3	–	0.066	
**Zn-1.5Mg**^**Cast**^	HBSS	30	–	–	0.065	0.104	[135]
**Zn-1.5Mg-0.1Ca**^**Cast**^		30	–	–	0.110	0.238	
**Zn-1.5Mg-0.1Sr**^**Cast**^		30	–	–	0.105	0.105	
**Zn**^**HE**^		56	–	–	0.078	0.135	[55]
**Zn-1.0Mg-1.0Ca**^**HE**^		56	–	–	0.092	0.170	
**Zn-1.0Mg-1.0Sr**^**HE**^		56	–	–	0.095	0.178	
**Zn-0.02Mg-0.02Cu**^**HE**^		15	–	–	0.047	0.079	[224]
**Zn**^**Cast**^		30	−1.12	3.52	0.086	0.05	[226]
**Zn-1.0Mg-0.1Mn**^**Cast**^		30	−1.23	17.21	0.12	0.26	
**Zn-1.5Mg-0.1Mn**^**Cast**^		30	−1.23	9.34	0.09	0.14	
**Zn-1.0Mg-0.1Mn**^**HR**^		30	−1.21	16.76	0.11	0.25	
**Zn**^**Cast**^		–	−1.11	3.02	–	0.05	[225]
**Zn–1Mg-0.1Sr**^**Cast**^		–	−1.23	7.85	–	0.12	
**Zn–1Mg-0.5Sr**^**Cast**^		–	−1.23	7.13	–	0.11	
**Zn–1Mg-0.1Sr**^**HR**^		–	−1.19	10.24	–	0.15	
**Zn**^**HE**^	SBF	90	−1.05	7.89	0.13	0.23	[227]
**Zn-0.5Mg-0.5Zr**^**HE**^		90	−1.23	5.44	0.08	0.16	
**Zn-1.0Mg-0.5Zr**^**HE**^		90	−1.25	27.15	0.12	0.79	
**Zn-1.5Mg-0.5Zr**^**HE**^		90	−1.26	68.41	0.09	2.00	
**Zn-0.1Ca**^**HE**^		30	−1.19	17.83	0.018	–	[213]
**Zn-0.4Ca**^**HE**^		30	−0.98	7.32	0.020	–	
**Zn-0.8Ca**^**HE**^		30	−1.20	10.51	0.021	–	
**Zn**^**Cast**^	HBSS	14	−1.192	2.41	0.023	0.036	[181]
**Zn-0.5Ca**^**Cast**^		14	−1.225	2.84	0.035	0.042	
**Zn-1.0Ca**^**Cast**^		14	−1.239	3.83	0.040	0.057	
**Zn-2.0Ca**^**Cast**^		14	−1.242	5.61	0.074	0.084	
**Zn-3.0Ca**^**Cast**^		14	−1.236	4.08	0.066	0.062	
**Zn-1.0Ca**^**Cast**^		56	−1.019	10.75	0.090	0.160	[147]
**Zn-1.0Ca-1Sr**^**HE**^		56	–	–	0.11	0.19	[55]
**Zn-4.0Ca-2Cu**^**PM**^		–	−1.503	667	–	2.60	[277]
**Zn-0.1Sr**^**HE**^	SBF	30	−1.02	7.13	0.014	–	[213]
**Zn-0.4Sr**^**HE**^		30	−1.13	20.46	0.016	–	
**Zn-0.8Sr**^**HE**^		30	−1.14	27.59	0.021	–	
**Zn-1.0Sr**^**Cast**^	HBSS	56	−1.031	11.76	0.095	0.175	[147]
**Zn-1.1Sr**^**HR**^	SBF	30	–	–	0.4	–	[216]
**Zn Alloyed with crucial elements (Cu, Fe and Mn)**							
**Zn-0.4Cu**^**HE**^	SBF	30	−0.98	7.69	0.015	–	[213]
**Zn-0.8Cu**^**HE**^		30	−1.10	37.16	0.026	–	
**Zn-2Cu**^**HE**^		30	−1.11	31.53	0.029	–	
**Zn**^**HE**^		20	–	–	0.022	–	[162]
**Zn-1Cu**^**HE**^		20	–	–	0.033	–	
**Zn-2Cu**^**HE**^		20	–	–	0.027	–	
**Zn-3Cu**^**HE**^		20	–	–	0.030	–	
**Zn-4Cu**^**HE**^		20	–	–	0.025	–	
**Zn-1Cu**^**LPBF**^		28	−1.167	9.12	0.16	0.137	[278]
**Zn-2Cu**^**LPBF**^		28	−1.186	10.96	0.18	0.165	
**Zn-3Cu**^**LPBF**^		28	−1.198	11.75	0.20	0.176	
**Zn-4Cu**^**LPBF**^		28	−1.238	12.88	0.22	0.194	
**Zn-4Cu**^**HE**^	HBSS	20	–	4.1	–	0.009	[149]
**Zn-1Cu**^**Cast**^		40	–	–	0.16	–	[228]
**Zn-2Cu**^**Cast**^		40	–	–	0.13	–	
**Zn-4Cu**^**Cast**^		40	–	–	0.14	–	
**Zn-1Cu**^**HE**^		40	–	–	0.06	–	
**Zn-2Cu**^**HE**^		40	–	–	0.13	–	
**Zn-4Cu**^**HE**^		40	–	–	0.13	–	
**Zn-3Cu**^**HE**^		20	−1.102	0.372	0.012	0.005	[163]
**Zn–3Cu-0.1Mg**^**HE**^		20	−1.000	1.177	0.023	0.018	
**Zn–3Cu-0.5Mg**^**HE**^		20	−0.957	1.563	0.030	0.024	
**Zn–3Cu-1.0Mg**^**HE**^		20	−0.945	12.413	0.043	0.180	
**Zn-3Cu**^**HE**^	SBF	20	−1.110	5.8	0.045	0.085	[105]
**Zn–3Cu-0.5Fe**^**HE**^		20	−1.095	7.1	0.064	0.105	
**Zn–3Cu-1.0Fe**^**HE**^		20	−1.087	8.8	0.069	0.130	
**Zn-3Cu**^**HE**^		20	–	–	0.043	–	[83]
**Zn–3Cu-0.2Fe**^**HE**^		20	–	–	0.059	–	
**Zn–3Cu-0.5Fe**^**HE**^		20	–	–	0.064	–	
**Zn-2Cu**^**Cast**^	HBSS	30	−1.137	1.63	0.011	–	[229]
**Zn–2Cu-0.05Ti**^**Cast**^		30	−1.164	2.56	0.022	–	
**Zn–2Cu-0.1Ti**^**Cast**^		30	−1.211	3.27	0.028	–	
**Zn–1Cu-0.1Ti**^**Cast**^		30	−1.025	21.5	0.029	0.315	[152]
**Zn–1Cu-0.1Ti**^**HR**^		30	−1.123	111.2	0.034	1.628	
**Zn–1Cu-0.1Ti**^**HR + Cold rolling**^		30	−1.100	67.7	0.032	0.991	
**Zn-3Cu**^**Cast**^	HBSS	90	−0.932	14.3	0.019	0.190	[252]
**Zn-3Cu**^**HR**^		90	−0.946	19.2	0.021	0.255	
**Zn-3Cu**^**HR + Cold rolling**^		90	−0.979	23.4	0.024	0.311	
**Zn–3Cu-0.2Ti**^**Cast**^		90	−0.961	10.9	0.018	0.145	
**Zn–3Cu-0.2Ti**^**HR**^		90	−0.982	19.0	0.021	0.252	
**Zn–3Cu-0.2Ti**^**HR + Cold rolling**^		90	−0.993	22.5	0.022	0.299	
**Zn-0.1Fe**^**HE**^	SBF	30	−1.14	49.36	0.020	–	[213]
**Zn-0.4Fe**^**HE**^		30	−1.04	9.20	0.016	–	
**Zn-0.8Fe**^**HE**^		30	−1.13	55.55	0.022	–	
**Zn-0.3Fe**^**Cast**^		30	−1.00	8.99	0.046	0.137	[192]
**Zn-0.3Fe**^**BCWC**^		30	−1.01	7.31	0.044	0.111	
**Zn**^**Cast**^	PBS	20	−1.02	0.67	0.295	0.010	[230]
**Zn-1.3Fe**^**Cast**^		20	−1.04	0.89	0.509	0.013	
**Zn**^**Cast**^		30	–	–	0.320	–	[272]
**Zn-4Fe**^**Cast**^		20	–	–	0.148	–	
**Zn-0.1Mn**^**HE**^	SBF	30	−0.95	5.07	0.028	–	[213]
**Zn-0.4Mn**^**HE**^		30	−0.96	7.13	0.014	–	
**Zn-0.8Mn**^**HE**^		30	−0.98	7.51	0.019	–	
**Zn-0.1Mn**^**HE**^		30	−0.964	9.031	0.027	0.161	[231]
**Zn-0.4Mn**^**HE**^		30	−0.942	10.671	0.016	0.318	
**Zn-0.8Mn**^**HE**^		30	−0.976	7.436	0.019	0.111	
**Zn-0.82Mn**^**HR**^	HBSS	15	−1.080	9.530	0.036	0.145	[279]
**Zn-0.82Mn**^**HR+SHT**^		15	−1.080	6.25	0.019	0.095	
**Zn**^**PM**^		3	−0.85	138	–	2.71	[139]
**Zn-4Mn**^**PM**^		3	−1.02	48	–	0.72	
**Zn-24Mn**^**PM**^		3	−1.35	2.08	–	0.02	
**Zn-0.8Mn**^**HE**^	SBF	–	−1.07	6.76	–	0.101	[232]
**Zn-0.8Mn - 0.4Ag**^**HE**^		–	−1.19	11.22	–	0.168	
**Zn-0.8Mn - 0.4Cu**^**HE**^		–	−1.18	8.91	–	0.133	
**Zn-0.8Mn - 0.4Ca**^**HE**^		–	−1.16	10.72	–	0.160	
**Zn-0.35Mn - 0.41Cu**^**HR**^		14	−1.06	4.1	0.050	0.062	[145]
**Zn-0.75Mn - 0.40Cu**^**HR**^		14	−1.03	6.5	0.065	0.098	
**Zn Alloyed with other elements**							
**Zn-0.4Ag**^**HE**^	SBF	30	−1.05	35.66	0.026	–	[213]
**Zn-0.8Ag**^**HE**^		30	−1.10	45.98	0.018	–	
**Zn-2Ag**^**HE**^		30	−1.06	17.27	0.018	–	
**Zn**^**HE**^	HBSS	14	−0.98	8.9	0.077	0.133	[102]
**Zn-2.5Ag**^**HE**^		14	−1.12	9.2	0.079	0.137	
**Zn-5.0Ag**^**HE**^		14	−1.12	9.7	0.081	0.144	
**Zn-7.0Ag**^**HE**^		14	−1.14	9.9	0.084	0.147	
**Zn-2Ag**^**SLM**^	SBF	21	−1.07	5.01	0.086	0.08	[141]
**Zn-4Ag**^**SLM**^		21	−0.99	1.47	0.107	0.02	
**Zn-6Ag**^**SLM**^		21	−0.94	9.56	0.114	0.15	
**Zn-8Ag**^**SLM**^		21	−0.90	13.94	0.133	0.21	
**Zn-1.0Ag**^**HE**^	HBSS	28	−1.035	12.3	–	0.184	[280]
**Zn-1.0Ag-0.05Zr**^**HE**^		28	−1.008	4.6	–	0.077	
**Zn-0.5Al**^**HE**^	AU	–	−1.15	77	–	1.14	[273]
**Zn-0.5Al**^**Cast**^	HBSS	14	−0.99	11.08	–	0.165	[178]
**Zn-0.5Al**^**HE**^		14	−0.98	9.60	0.079	0.143	
**Zn-1.0Al**^**Cast**^		14	−0.99	11.11	–	0.166	
**Zn-1.0Al**^**HE**^		14	−0.98	9.70	–	0.145	
**Zn-2.0Al**^**LPBF**^	SBF	14	−1.059	8.037	–	0.142	[143]
**Zn**^**Com**^		7	−1.032	9.55	–	0.14	[281]
**Zn–5Al-4 Mg**^**Com**^		7	−1.020	17.7	–	0.32	
**Zn-0.5Al**^**Cast**^		30	−1.074	20.4	–	0.147	[136]
**Zn-0.5Al-0.1Mg**^**Cast**^		30	−1.065	17.3	–	0.130	
**Zn-0.5Al-0.3Mg**^**Cast**^		30	−1.034	11.2	–	0.110	
**Zn-0.5Al-0.5Mg**^**Cast**^		30	−1.018	9.5	–	0.080	
**Zn-0.5Al-0.5Mg**^**Cast**^		30	−1.028	9.51	0.148	–	[205]
**Zn-0.5Al-0.5Mg-0.1Bi**^**Cast**^		30	−1.049	12.11	0.174	–	
**Zn-0.5Al-0.5Mg-0.3Bi**^**Cast**^		30	−1.065	16.45	0.210	–	
**Zn-0.5Al-0.5Mg-0.5Bi**^**Cast**^		30	−1.084	22.73	0.283	–	
**Zn**^**Com +**^^**HE**^	HBSS	–	−0.958	1.799	0.027	–	[236]
**ZA4-1**^**Com +**^^**HE**^		–	−1.145	2.986	0.047	–	
**ZA4-3**^**Com +**^^**HE**^		–	−1.196	7.209	0.374	–	
**ZA6-1**^**Com +**^^**HE**^		–	−1.142	5.331	0.086	–	
**Zn**^**Cast**^		14	−1.063	10.7	0.068	0.157	[168]
**Zn-5.0Ge**^**Cast**^		14	−1.020	8.7	0.042	0.127	
**Zn**^**HR**^		14	−1.077	20.9	0.099	0.306	
**Zn-5.0Ge**^**HR**^		14	−1.045	15.4	0.051	0.226	
**Z-0.1Li**^**HE**^	SBF	30	−1.04	15.20	0.025	–	[213]
**Z-0.4Li**^**HE**^		30	−1.03	11.26	0.019	–	
**Z-0.8Li**^**HE**^		30	−1.11	30.40	0.025	–	
**Zn**^**HR**^		14	−1.35	10.96	–	0.16	[103]
**Zn-0.2Li**^**HR**^		14	−1.18	3.98	–	0.06	
**Zn-0.4Li**^**HR**^		14	−1.21	3.80	–	0.05	
**Zn-0.8Li**^**HR**^	Ringer's	35	−1.29	8.24	–	0.12	[239]
**Zn-0.8Li-0.2Ag**^**HR**^		35	−1.21	7.67	–	0.11	
**Zn-0.8Li-0.2Mg**^**HR**^		35	−1.32	11.31	–	0.17	

Com: Commercial, SHT: Solution heat treatment, PBS: phosphate buffered saline; HBSS: HBSS balanced salt solution; SBF: stimulated body fluid; AU: Artificial urine; WB: Whole blood; AP: Artificial plasma; HP: Human plasma; RSS: Ringer's saline solution; MEM: minimum essential medium; E_corr_: electrochemical voltage; I_corr_: current density; C.R: corrosion rate; C. R_imm_: corrosion rate evaluated from immersion corrosion studies; C. R_ele_: corrosion rate evaluated from electrochemical corrosion studies.

Mostaed et al. [178] evaluated and compared the degradation behaviors of as-cast and extruded Zn-xMg (x = 0.15, 0.5, 1.0, and 3.0) alloys in HBSS. Compared to the as-cast alloys, electrochemical studies of these alloys revealed lower corrosion potential (E_corr_) and current density (I_corr_) values for the extruded Zn–Mg alloys. The *in vitro* degradation of an ECAP-processed Zn–3Mg alloy in SBF was investigated by Dambatta et al. [160]. Their results indicated that compared to as-cast alloys, the ECAP-processed alloys had better corrosion resistance, which was attributed to grain refinement in these alloys. The corrosion behaviors of as-rolled pure Zn and binary Zn-1X (x = Mg, Ca, Sr) alloys in HBSS solution for 14 and 56 d were also investigated by Li et al. [147]. Electrochemical studies of these alloys indicated that pure Zn had better corrosion resistance than as-rolled Zn-1X alloys and the corrosion rates of these alloys were in the order: pure Zn < Zn–1Mg < Zn–1Ca < Zn–1Sr. Initially, after 14 d immersion in HBSS solution, the surfaces of all samples were found to be flat; however, more apatite-like corrosion products were deposited on the surfaces of the Zn-1X alloys than on the pure Zn after immersion for 56 d. The strength of these Zn-1X alloys decreased slightly after 8 weeks’ immersion in HBSS, but these alloys retained reasonable mechanical integrity, showing their greater suitability for orthopedic applications compared to Mg-based alloys [270].

The *in vitro* corrosion behavior of Zn alloyed with crucial elements (Cu, Mn and Fe) were reported by several researchers. For instance, Tang et al. [162] studied the *in vitro* degradation behavior of Zn-xCu alloys with Cu contents from 1 to 4 wt% in c-SBF solution at 37 ᴼC for 20 days immersion period, and reported that inclusion of Cu into Zn slightly enhanced CR of Zn, but CR of alloys remained almost steady. Same research group in another study reported a lower CR of 9.4 μm/y in case of Zn–4Cu alloy in HBSS. Hou et al. [271] reported that addition of 3 wt% Cu into Zn scaffolds in c-SBF solution significantly raised the CR due to precipitation of CuZn_5_ second phase. Kafri et al. [230] developed the Zn-1.3Fe (wt.%) alloy and computed the CR in PBS solution. The acquired values of V_corr_, I_corr_ and CR of Zn-1.3Fe alloy was found to be increased notably compared to pure Zn for immersion period of 20 days (Table 6). However, in another study, they reported a reduction of CR in Zn–4Fe alloy with respect to pure Zn in the same corrosion medium owing to the passivation effect of corrosion products [272]. Addition of Mn was noted to shrink the CR of pure Zn, such as, addition of 4 and 24 wt% Mn in pure Zn dropped the CR of Zn more than 3 and 100 times, respectively, which was ascribed to finer microstructure and the formation of a fewer intermetallic compound [139].

The *in vitro* corrosion behavior of Zn alloys with other element, i.e., Ag, Al, Ge, Li, Zr were also studied and obtained results are summarized in Table 6. Like Ca, Sr and Cu, the addition of Ag [102] and Al [178], were reported to increase CR of extruded Zn alloys, conversely, CR of Zn alloys was found to be abated by adding Ge [168] and Li [103]. For instance, the corrosion properties of as-cast and HR Zn-5Ge alloy was studied by Tong et al. [168] in HBSS and noted that the CR rate of HR Zn-5Ge was almost double of the CR of as-cast Zn-5Ge. However, in both cases the CR of Zn-5Ge was much lower than that of pure Zn. Champagne et al. [273] compared the electrochemical CR of HE pure Zn, Zn-0.5Al and Zn-0.5 Mg in AU, and reported that the CR of both alloys was decreased compared to Zn counterparts, but the CR of Zn-0.5 Mg was superior than that of Zn-0.5Al alloy. For Zn based ternary alloys, Bakhsheshi-Rad et al. [136] observed that the addition of Mg (0.1–0.5) progressively increased the CR of Zn-0.5Al (Table 6). In another study, they also reported the similar degradation behavior of as cast Zn-0.5Al-0.5 Mg alloys with addition of Bi, and CR was increased from 0.148 to 0.283 mm/y [205]. Zhang et al. [239] investigated the degradation behavior of Zn-0.8Li, Zn-0.8Li-0.2Ag, and Zn-0.8Li-0.2 Mg, and their CRs were measured as 0.12 mm/y, 0.11 mm/y, and 0.17 mm/y, respectively. Higher CR of Zn-0.8Li-0.2 Mg was attributed to the formation of cathodic sites due to formation of intermetallic compounds. Nevertheless, lower CR of these alloys showed suitability for bone regeneration implant applications. Recently, Li et al. [212] investigated the *in vitro* degradation behavior of Zn-xLi (x = 0.1–1.4 wt%) alloys in SBF and the results showed the ability of Li in forming LiOH and Li_2_CO_3_-rich passivation films as corrosion products on Zn-(0.5–1.4 wt %)Li alloys. Formation of these passive films resulted in reduction of CR of Zn-1.4Li alloy (14.26 μm/y) than that of pure Zn (45.76 μm/y). They also reported that interestingly β phase degraded prior to Zn in the alloys, indicating that biomedical implants made of Zn–Li alloys are likely to degrade entirely in human body.

The *in vitro* corrosion behaviors of Zn-based alloyed with various elements have also been reported in many other studies and are summarized in Table 6.

### 5.2 In vivo degradation behavior of Zn-based materials

Various animal models such as those using Sprague–Dawley (SD) rats [78,282,283], Wistar rats [230,272,284], C57BL/6 mice [147], beagle dogs [87], white pigs [285], and rabbits [90,148] have been used in previous studies for *in vivo* degradation assessment of metallic biomaterials. The implantation sites in these animals are generally application-driven. For example, Zn was inserted into the abdominal aorta for *in vivo* assessment for cardiovascular stent applications [101,151,286], whereas it was implanted in the bone for evaluation of orthopedic applications [147,148]. Pierson et al. [287] proposed a novel and inexpensive technique for implanting wire into the arterial wall and this method was adopted by several studies [78,282,288]. The corrosion rate of an implanted material can be assessed by measuring the weight loss after a specific post-implantation duration. However, Bowen et al. [289] reported the unsuitability of this method for evaluating the *in vivo* corrosion rates of samples with large aspect ratios, e.g., metallic wires. Therefore, a new approach was proposed in their study to determine the corrosion penetration rate (CPR) by determining the reduced cross-sectional area of the specimen against implantation time, using [289]:

(11) $$CPR=\frac{\sqrt{\frac{A_{o}}{\pi }}-\sqrt{\frac{A_{t}}{\pi }}}{t}$$

where, *t*, *A*_*o*_, and *A*_*t*_ are the implantation duration, original cross-sectional area, and cross-sectional area after a specific duration, respectively. In another study, Li et al. [147] also proposed a new method to determine *in vivo* corrosion rates by employing micro-CT imaging, using:

(12) $$C.R_{in\;vivo}=\;\frac{V_{0}-V_{t}}{At}$$

where *A*, *V*_*o*_, and *V*_*t*_ are the initial surface area of the implant, the initial volume of the implant, and the volume of the implant after a specific duration, respectively.

**Fig. 15:**
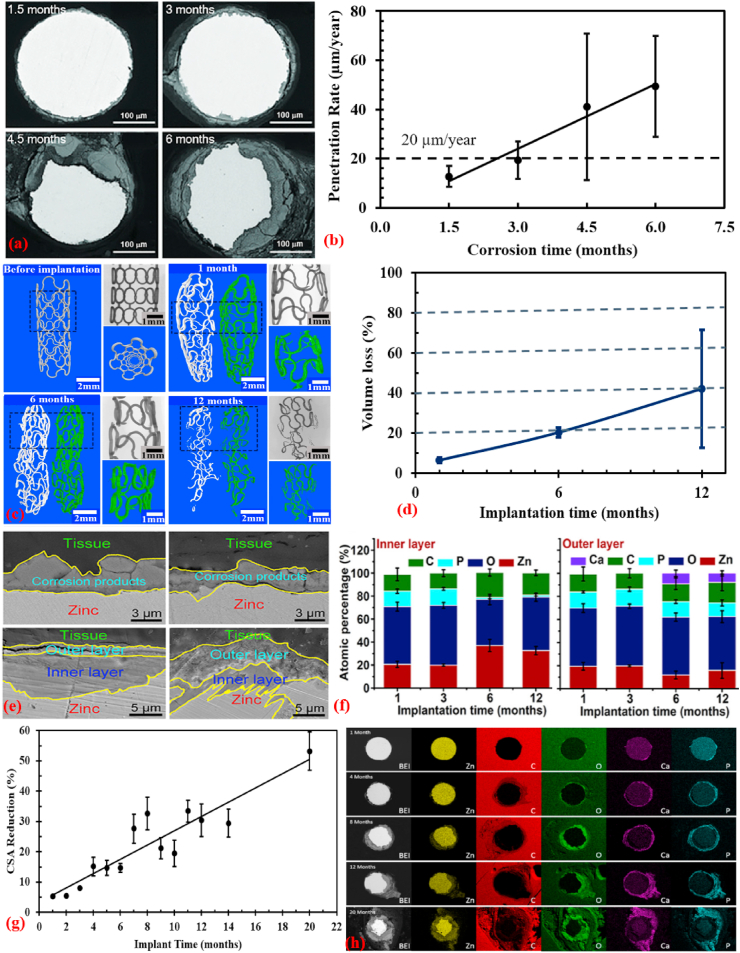
In vivo degradation behavior of Zn-based implants: (a) cross-sectional changes of Zn implant after 1.5, 3, 4.5 and 6 months in the abdominal aorta of rats, (b) CPR rate of implanted Zn wires as a function of exposure time, (c) micro-CT images of Zn stents after 0, 1, 6 and 12 months of implantation, (d) volume loss of Zn stent at different exposure duration, (e) SEM images of corrosion products formed on Zn implant after 1, 3, 6 and 12 months implantation, (f) elemental compositions of corrosion products (at. %) in inner and outer corrosion layers formed after 1, 3, 6, and 12 months implantation, (g) Reduction of cross-sectional area in Zn implant after different exposure time, and (h) energy dispersive X-ray (EDX) elemental maps showing the variation in cross-sectional area of corroded Zn wires. (Reproduced with permission from Refs. [78,90,283]).

**Fig. 16:**
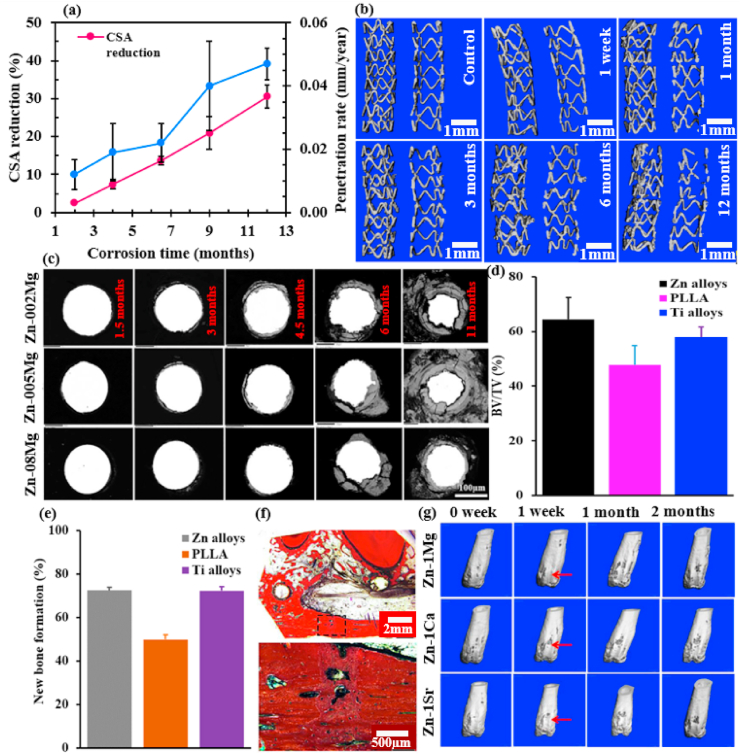
In vivo corrosion behavior of Zn-based alloys: (a) CSA reduction and penetration rate of implanted Zn–Li wires as a function of corrosion time, (b) micro-CT 3D images of Zn–Mg–Cu alloy stents after 1 week, 1, 3, 6, and 12 months of implantation in the rabbit carotid arteries (Each time point is composed of two images: the left one is a complete 3D reconstruction and the right one is a lengthwise section of the stent), (c) cross-sectional changes from different implantation Zn–Mg wires after 1.5, 3, 4.5, 6 and 11 months' *in vivo*, (d) BV/TV with implantation time for the Zn (Zn-2.5Mg-2.5Fe) alloys, PLLA and Ti alloys at 4 weeks post operation, (e) percentage of new bone formation for the Zn alloys, PLLA and Ti alloys at 4 weeks implantation time, (f) undecalcified bone histomorphometry of the mandibles at 12 weeks post operation in the Zn alloys, and (g) micro-CT 3D images of Zn-1X pin group implanted in the femora of mice, red arrows show the increase in bone density. (Reproduced with permissions from Ref. [87,101,147,151,165]).

The *in vivo* degradation behaviors of pure Zn and its alloys have been reported in several studies [87,101,151,165,283] and some of the results obtained for pure Zn are summarized in Fig. 15, while those for Zn-based alloys are presented in Fig. 16. For example, Bowen et al. [78] first reported the *in vivo* corrosion rate of a pure Zn implant placed into the abdominal aorta of SD rats. The corrosion rate of the Zn implant, which was measured by the post-implantation changes in the cross-sectional area, increased gradually over time in the aorta, as shown in Fig. 15a and b. Similarly, Yang et al. [90] investigated the *in vivo* degradation behavior of pure Zn stent in a rabbit model. The implanted Zn stent retained its mechanical integrity up to 6 months, but degraded almost 42% of its volume after 1 year of implantation, as shown in Fig. 15c and d. Their study also investigated the chemical composition of the corrosion products that were formed on the Zn stent, as shown in Fig. 15e and f. The inner layer of the corrosion was composed of Zn, carbon (C), phosphate (P), and oxygen (O_2_), while the outer layer contained the additional element of Ca (Fig. 15f). Recently, Drelich et al. [283] investigated the *in vivo* degradation behavior of Zn wires implanted in the murine artery for a long duration (up to 20 months). Their study revealed a stable degradation rate of the implant for at least a 20-month period, as shown in Fig. 15g. They observed that degraded Zn wire was substituted for by corrosion products, as shown in Fig. 15h. The cross-sectional area of the wire changed due to the deposition of corrosion products; however, the implant retained its original shape.

Zhao et al. [101] implanted Zn and Zn–Li wires in the abdominal aorta of SD rats for 2–12 months and observed that a reduction in the cross-sectional area of the alloys progressively increased with implantation time, resulting in loss of the circular wire's integrity (Fig. 16a). Lin et al. [165] implanted Zn-0.02Mg-0.02Cu stents in rabbit carotid arteries for maximum 1 year and obtained micro-CT 3D reconstruction images, as shown in Fig. 16b, which revealed that after a 1-week corrosion period, the stent had almost no corrosion; later, the stent was partly corroded by fracturing and attenuating struts, yet it was found to be almost intact after up to 6 months of implantation time, although acute localized corrosion with vanishing several struts was noticed after 1 year of implantation. The *in vivo* degradation behavior of Zn-xMg (x = 0.002, 0.005, 0.08 wt%) alloys was studied by Jin et al. [151] using an SD rat model for a 1.5–11 month implantation period and they reported that with progression of time, the cross-sectional area of the metallic specimens became shorter with a more irregular shape, as shown in Fig. 16c. They also reported that the CR of Zn-0.002 Mg and Zn-0.005 Mg alloys at 1.5 months were higher than that of P–Zn but very close to the benchmark value for an endovascular stent (0.02 mm/y) [290]. However, the CR of Zn-0.008 Mg were found to increase to 0.027 mm/y after 6 months' implantation time. Bowen et al. [146] investigated the *in vivo* corrosion behaviors of P–Zn and Zn-xAl alloys (x = 1, 3, 5 wt%) by inserting strips of these alloys into the arterial wall of SD rats for 6 months, and reported that the P–Zn was corroded from the surface into the interior. A segment of the P–Zn strip stayed intact after 6 months' exposure, but earlier (within 1.5 months or even less) degradation and cracking were observed in the strips of Zn–Al alloys. The *in vivo* CR was found to rise with increasing wt.% of Al and the most notable fragmentation was seen in the Zn–5Al among all the investigated Zn–Al alloys. Wang et al. [87] implanted a Zn alloy (Zn-2.5Mg-2.5Fe), a Ti alloy, and poly-l-lactic acid (PLLA) into beagle dogs for 6 months to evaluate and compare the *in vivo* degradation behaviors, and observed that after 4 weeks the size of the bone calluses in PLLA was much greater than those in the Ti and Zn alloys; however, histomorphometry at 4 weeks revealed that the values for bone volume/total volume, BV/TV (Fig. 16d), and trabecular thickness (Tb·Th) were notably improved in the Zn alloy, which indicates that the Zn-based implants caused fast bone remodeling during fracture healing (Fig. 16e). This was also confirmed by the result of undecalcified bone histomorphometry, as shown in Fig. 16f. Similar to the ternary Zn-2.5Mg-2.5Fe alloy, Li et al. [147] observed that binary Zn–1Mg, Zn–1Ca, and Zn–1Sr alloys were also able to influence new bone formation. They implanted several pins of these three alloys into the femora of mice from 1 to 8 weeks, and the micro-CT 3D images in Fig. 16g clearly indicate that after 7 d there were successive variations in the bone at the distal femora, which suggests new bone formation and remodeling. The *in vivo* corrosion and biocompatibility properties of several biodegradable Zn and Zn alloys are summarized in Table 7.

**Table 7:** In vivo corrosion and biocompatibility of biodegradable Zn and Zn alloys.

Materials	**Animal model (implanted site)**	**Duration (months)**	**Corrosion rate (mm/y)**	Residual area (vol%)		Biocompatibility		Key findings	Refs.	
Pure Zn										
Zn (wire)	SD rat (aorta)	1.5	0.012	97	♦♦♦		Zn wire remained intact up to 4 months and then corrosion accelerated. The corrosion products on Zn after 4.5 and 6 months were mainly made of ZnO and ZnCO3.			[78]
		3.0	0.02	93						
		4.5	0.042	76						
		6.0	0.048	63						
	SD rat (aorta)	2.5	–	–	♦♦♦		Low cell densities and neointimal tissue thickness, along with tissue regeneration within the corroding implant, point to optimal biocompatibility of corroding zinc			[282]
		6.5	–	–						
ZnExt&Dwg (wire)	SD rat (aorta)	2	0.020	95	♦♦♦		Extruded Zn wire exhibited nearly linear relationship between the % of area reduction (AR) and time, and uniform gradual acceleration of biodegradation and moderate inflammation with nonobstructive neointima.			[101]
		4	0.030	85						
		6	0.019	85						
		8	0.035	–						
		10	0.016	–						
		12	0.023	69						
Zn (wire)	SD rat (aorta)	3	0.020	92	♦♦♦		Zn wires exhibited steady corrosion without local toxicity for up to at least 20 months post implantation, despite a steady build-up of passivating corrosion products and intense fibrous encapsulation of the wire.			[283]
		6	0.019	85						
		9	0.019	79						
		12	0.023	70						
		14	0.019	70						
		20	0.026	47						
ZnExt (rod)	SD rat (femur)	2	0.137	95	♦♦♦		Dark brown degradation products spread into the surrounding tissue with newly formed woven bone dispersed in it.			[213]
Zn Alloyed with nutrient elements (Ca, Mg and Sr)										
Zn-1CaHR (pin)	Mice (femur)	2	0.190	–	♦♦		Promoted bone growth.			[147]
Zn-0.8CaHE (rod)	SD rat (femur)	2	0.130	95	♦♦		A greater amount of new bone tissues (NBTs) were observed surrounding the implants and the osteocytes in the new bone tissue arranged in an organized way.			[213]
Zn-0.002MgHE + DW (wire)	SD rat (aorta)	1.5	0.029	95	♦♦♦		Zn–Mg alloys displayed uniform degradation and the increase of degradation rates in later stages of implantation was detected. Slightly decrease in biocompatibility with increasing Mg content was observed.			[151]
		3	0.020	92						
		4.5	0.027	85						
		6	0.033	77						
		11	0.051	43						
Zn-0.005MgHE + DW (wire)	SD rat (aorta)	1.5	0.021	96	♦♦					
		3	0.020	93						
		4.5	0.023	87						
		6	0.030	78						
		11	0.039	54						
Zn-0.08MgHE + DW (wire)	SD rat (aorta)	1.5	0.012	98	♦♦					
		3	0.013	95						
		4.5	0.015	91						
		6	0.027	80						
		11	0.023	71						
Zn-0.8MgHE (rod)	SD rat (femur)	2	0.146	95			A lot of NBTs were observed surrounding the implants with no signs of osteolysis, deformity or dislocation.			[213]
Zn-1MgHR (pin)	Mice (femur)	2	0.170	–	♦♦♦		Promoted bone growth.			[147]
Zn–1Mg-0.1CaHE (rod)	SD rat (subcutaneous)	3	0.050	–	♦♦		Zn–Mg–Ca alloys could be safely used by adding Mg to adjust the degradation property.			[180]
Zn-0.02 Mg-0.02CuHE + DW (stent)	New Zealand rabbits (artery)	1	0.078	83	♦♦♦		The stent corroded slowly, and no obvious intimal hyperplasia was observed till 6 months. After that corrosion accelerated. In addition, no obvious thrombosis and systemic toxicity during implantation period were observed.			[165]
		3	0.027	83						
		6	0.023	71						
		12	0.040	42						
Zn-2.5Mg-2.5FeHE + DW (rod)	Beagle dogs (dorsal)	1	0.033	–	♦♦		Zinc-based alloy osteosynthesis system possessed uniform and slow corrosion leading to adequate degradation behavior in 6 months.			[87]
		3	0.078	–						
		6	0.094	–						
Zn-0.1SrHE (rod)	SD rat (femur)	2	0.127	95.5	♦♦		A great proportion of NBTs were observed surrounding the implants.			[213]
Zn-1SrHR (pin)	Mice (femur)	2	0.220	–	♦♦		Promoted bone growth.			[147]
Zn-1.1SrHR (wire)	SD rat (aorta)	1	–	–	♦♦		New bone formation was observed around the implant. Also, some fibrotic and collagenous tissues between the implants and newly formed bones were also observed.			[216]
Zn Alloyed with crucial elements (Cu, Fe and Mn)										
Zn-0.4CuHE (rod)	SD rat (femur)	2	0.250	92	♦♦		Dark brown degradation products spread into the surrounding tissue with newly formed woven bone dispersed in it.			[213]
Zn-0.8CuCast (stent)	White pigs (artery)	3	–	92	♦♦		Stent provided sufficient structural support and exhibited an appropriate degradation rate during 24 months of implantation without degradation product accumulation, thrombosis, or inflammation response.			[285]
		6	–	79						
		9	–	77						
		12	–	74						
		18	–	56						
		24	–	28						
Zn-0.4FeHE (rod)	SD rat (femur)	2	0.150	94.5	♦♦		Dark brown degradation products spread into the surrounding tissue with newly formed woven bone dispersed in it.			[213]
Zn-1.3FeCast (disk)	Wistar rat (subcutaneous)	3.5	0.115	–	♦♦		The implantation of alloy did not increase the amount of zinc in blood beyond the acceptable level and there were no signs of infection.			[230]
Zn-2FeCast (disk)	Wistar rat (subcutaneous)	3.5	0.115	–	♦♦		No signs of anemia, inflammation or necrosis.			[284]
		6.0	0.055	–						
Zn-0.1MnHE (rod)	SD rat (femur)	2	0.127	96	♦♦		New bone tissues were observed surrounding the implants. Osteocytes in the new bone tissue arranged in organized way.			[213]
Zn Alloyed with other elements										
Zn-1AlHR (strips)	SD rat (aorta)	1.5	–	83	♦♦		The alloys showed acceptable biocompatibility with surrounding arterial tissue. No necrotic tissue was detected, while some inflammation was observed. Biocorrosion rates were higher at initial stages than that of pure Zn.			[288]
		3	–	81						
		4.5	–	66						
		6	–	50						
Zn-3AlHR (strips)		1.5	–	67	♦♦					
		3	–	66						
		4.5	–	62						
		6	–	52						
Zn-5AlHR (strips)		1.5	–	89	♦♦					
		3	–	75						
		4.5	–	67						
		6	–	57						
Zn-2AgHE (rod)	SD rat (femur)	2	0.187	93.5	♦♦		A localized degradation mode was observed with new bone formation and direct contact between new bone and implants at 8 weeks.			[213]
Zn-0.1LiHE + DW (wire)	SD rat (aorta)	2	0.008	98	♦♦		The alloy degraded ~ 30% of its original volume after 12 months and revealed almost linear relationship with the % of AR and time, indicating uniform gradual acceleration of biodegradation. Medium inflammation with non-obstructive neointima was observed.			[101]
		4	0.016	92						
		6.5	0.019	86						
		9	0.038	79						
		12	0.045	70						
Zn-0.4LiHE (rod)	SD rat (femur)	2	0.156	93.5	♦♦		Implant maintained its integrity at 8 weeks and degraded uniformly. Larger amounts of NBTs were observed surrounding the implants.			[213]

SD rat: Sprague–Dawley rat; biocompatibility rating: ♦♦♦- excellent, ♦♦- good, ♦- poor.

## 6 Zn-based composites

Compared to Mg and Fe, Zn-based materials show intermediate corrosion rates, as revealed by several studies which have been discussed in previous sections. In these studies, pure Zn was alloyed with several alloying elements such as Mg, Ca, Sr, Cu, Mn, Fe, Ag, Al, Ge, Ti, Zr etc. to improve its mechanical and corrosion properties, which have been summarized in Table 5, Table 6. Several studies have also reported the promising mechanical, corrosion, and biocompatibility properties of Zn-based composites containing various reinforcement materials [91,[291], [292], [293], [294]]. These studies revealed considerable increases in the mechanical strength of pure Zn matrices containing bio-inert and bioactive ceramic reinforcement materials such as calcium phosphate (CaP), hydroxyapatite (HA), Bioglass, and tri-calcium phosphate (β-TCP) [91,293,294]. Table 8 summarizes the properties and applications of these various reinforcement materials for Zn-based composites.

**Table 8:** Properties and biomedical applications of various ceramic reinforcement materials [40,295,296].

Type	Properties	Applications
**Alumina (Al**_**2**_**O**_**3**_**)**	Excellent abrasion resistance and compressive strength. Bioinert.	Femoral head, knee prosthesis, bone screws and plates, and permeable coatings for stems.
**Zirconia (ZrO**_**2**_**)**	High fracture toughness and flexural strength, but low Young's modulus. Bioinert and biocompatible.	Artificial knees, bone screws and plates etc.
**Bioglass**	Biocompatible, bioactive and nontoxic. Poor ductility.	Wound healing, cochlear implants, and peripheral nerve, spinal cord and ligament repair.
**Hydroxyapatite (HA)**	Bioresorbable, bioactive and biocompatible; Composition and properties closer to natural bone.	Femoral knee, femoral hip, tibial components, acetabular cup.
**Tri-calcium phosphate (β-TCP)**	Bioresorbable, bioactive and biocompatible; Similar composition to natural bone and good osteoconductive.	Femoral knee, hip prostheses, tibial components, bone plates and screws and cardiovascular stents.

### 6.1 Bioceramic reinforcements in Zn-based composites

Ceramic biocomposites may contain various reinforcing particles such as tungsten carbide (WC), Titanium diboride (TiB_2_), alumina (Al_2_O_3_), zirconia (ZrO_2_), HA, and β-TCP. Al_2_O_3_ is a chemically inert material and possesses excellent hardness and abrasion resistance, which may increase the life span of Zn-based implant materials. Its excellent wear and friction properties *in vivo* further suggest its suitability for artificial-joint surfaces [297]. The chemical inertness of Al_2_O_3_ is advantageous for biomedical applications as it results in excellent biocompatibility and non-sensitization of tissues [298]. From the perspective of mechanical properties, the superior compressive strength of Al_2_O_3_ makes it better suited to hard-tissue applications under compressive loading, such as artificial joints and dental applications. Similarly, because of its bio-inertness, non-toxicity, high mechanical strength, and fracture toughness, ZrO_2_ is also used in orthopedic applications [299]. HA (Ca_10_(PO_4_)_6_(OH)_2_) is another calcium phosphate–based bioceramic material which has been extensively used as a reinforcing material in Fe- [300], Mg- [301,302], and Zn-based biodegradable matrices [91,303]. HA possesses a hexagonal crystal structure with a characteristic Ca/P ratio of 1.67. β-TCP has similar compositional properties to human bone and is extensively used for orthopedic applications due to its excellent biocompatibility with bone cells and its potential to stimulate new bone ingrowth [304]. Therefore, the addition of β-TCP particles as reinforcements in biodegradable metal matrices not only enhances their mechanical properties, but also significantly increases the biocompatibility of these composite materials [293]. Table 9, Table 10 summarize the mechanical and corrosion properties of various Zn-based composites, respectively.

**Table 9:** Mechanical properties of various Zn-based composites.

Composition & Fabrication	**Relative Density (%)**	Mechanical properties						Ref.
		σ_TYS_ (MPa)	σ_UTS_ (MPa)	E (%)	σ_CYS_ (MPa)	σ_UCS_ (MPa)	H (HV)	
**Zn**^**SPS**^	99.524	–	–	–	53 ± 18	171 ± 14	43.8 ± 1.3	[91]
**Zn-1HA**^**SPS**^	96.503	–	–	–	70 ± 11	158 ± 8	45.7 ± 1.5	
**Zn-5HA**^**SPS**^	94.925	–	–	–	43 ± 11	109 ± 10	43.6 ± 1.0	
**Zn-10HA**^**SPS**^	92.995	–	–	–	47 ± 10	74 ± 5	44.3 ± 1.5	
**Zn**^**HE**^	98.879	–	–	–	154 ± 11	244 ± 2	45.6 ± 2.0	[305]
**Zn-8HA**^**HE**^	94.480	–	–	–	113 ± 5	169 ± 4	44.7 ± 4.5	
**Zn-3HA**^**PM**^	–				110.56	–	–	[310]
**Zn**^**SPS**^	–	–	–	–	92 ± 1	129 ± 2	36.8 ± 1.4	[311]
**Zn-8HA**^**SPS**^	–	–	–	–	68 ± 7	89 ± 7	34.3 ± 4.5	
**Zn**^**SPS**^	–	–	–	–	81 ± 5	–	33 ± 2	[312]
**Zn-16HA**^**SPS**^	–	–	–	–	46 ± 3	65 ± 5	24 ± 5	
**Zn**^**SPS**^	99.130	–	–	–	54 ± 17	–	42.9 ± 2.3	[292]
**Zn–1Mg**^**SPS**^	99.91	–	–	–	134 ± 16	–	75.7 ± 9.5	
**Zn–2Mg**^**SPS**^	100.0	–	–	–	152 ± 11	–	69.5 ± 3.3	
**Zn–5Mg**^**SPS**^	100.0	–	–	–	183 ± 27	–	80.8 ± 9.9	
**Zn**^**PM**^	–	114 ± 5	156 ± 5	35 ± 4	170 ± 6	215 ± 4	–	[291]
**Zn-5Mg**^**PM**^	–	148 ± 6	183 ± 4	16 ± 2	209 ± 6	256 ± 6	–	
**Zn-1Mg**^**HE**^	–	226.2	300.5	5.8	–	–	–	[294]
**Zn–1Mg-1β-TCP**^**HE**^	–	249.3	330.1	11.7	–	–	–	
**Zn–1Mg-0 TCP**^**HE**^	–	236 ± 5	315 ± 8	6.7 ± 2	–	–	–	[293]
**Zn–1Mg-1 TCP**^**HE**^	–	251 ± 7	331 ± 9	11.7 ± 3	–	–	–	
**Zn–1Mg-3 TCP**^**HE**^	–	220 ± 8	308 ± 7	4.5 ± 2	–	–	–	
**Zn–1Mg-5 TCP**^**HE**^	–	195 ± 8	299 ± 9	3.9 ± 3	–	–	–	
**Zn–2Mg-6SiC**^**PM**^	90.30	–	–	–	–	225	96.79	[313]
**Zn**^**CC+M**^	–	–	–	–	23.4 ± 2.1	51 ± 3.6	40.6	[307]
**Zn-10 vol% WC**^**CC+M**^		–	–	–	116 ± 11	507 ± 65	60.1	
**Zn**^**HR**^	–	36.6	98.9	71.9	–	–	34.4	[309]
**Zn-3 vol% TiB2**^**HR**^	–	70.2	143.8	22.8	–	–	63.9	
**Zn-2Fe**^**Cast**^	–	66 ± 10	92 ± 12	1.4 ± 0.4	–	–	47.9	[308]
**Zn–2Fe-8 vol% WC**^**Cast**^	–	50 ± 2	121 ± 6	8.7 ± 2.8	–	–	59.3	
**Zn–2Fe-6 vol% WC**^**HR**^	–	92 ± 8	156 ± 6	15.2 ± 0.2	–	–	–	
**Zn**^**PM**^	–	–	–	–	79.29	83.45	27.0 ± 3.7	[314]
**Zn-1ND**^**PM**^	97.872	–	–	–	59.69	73.56	12.1 ± 4.9	
**Zn-2.5ND**^**PM**^	91.040	–	–	–	43.39	58.81	8.4 ± 0.15	
**Zn-0MWCNTs**^**SPS+HR**^	–	–	123	–	–	–	65.7	[315]
**Zn-0.5MWCNTs**^**SPS+HR**^	–	–	151	–	–	–	71.7	
**Zn-1MWCNTs**^**SPS+HR**^	–	–	174	–	–	–	73.0	
**Zn-1.5MWCNTs**^**SPS+HR**^	–	–	217	–	–	–	80.4	
**Zn-2MWCNTs**^**SPS+HR**^	–	–	267		–	–	87.8	
**Zn-3MWCNTs**^**SPS+HR**^	–	–	281	4	–	–	89.6

**Table 10:** Degradation and corrosion properties of various Zn-based composites.

Composition &Fabrication	Corrosion medium	Immersion time (day)	Corrosion properties				Ref.
			Icorr (μA/cm^2^)	Ecorr (V)	C.R_(elec)_ (mm/y)	C.R_(imm)_ (mm/y)	
**Zn**^**SPS**^	HBSS	50	4.900 ± 2.810	−0.942 ± 0.075	0.073 ± 0.042	0.0048	[91]
**Zn-1HA**^**SPS**^		50	21.076 ± 3.251	−1.281 ± 0.037	0.327 ± 0.050	0.0026	
**Zn-5HA**^**SPS**^		50	39.127 ± 0.661	−1.274 ± 0.005	0.630 ± 0.011	0.0101	
**Zn-10HA**^**SPS**^		50	51.044 ± 1.803	−1.290 ± 0.010	0.856 ± 0.031	0.0250	
**Zn**^**HE**^	SBF	14	–	–	–	0.15	[305]
**Zn-8HA**^**HE**^		14	–	–	–	0.40	
**Zn-3HA**^**PM**^		–	5.164	−1.070	0.084 ± 0.012	–	[310]
**Zn**^**SPS**^		14	–	–	0.9	0.26	[312]
**Zn-16HA**^**SPS**^		14	–	–	1.5	0.41	
**Zn**^**SPS**^	HBSS	50	5.730 ± 2.777	−0.978 ± 0.026	0.085 ± 0.041	–	[292]
**Zn–1Mg**^**SPS**^		50	7.550 ± 0.900	−1.066 ± 0.104	0.114 ± 0.012	–	
**Zn–2Mg**^**SPS**^		50	13.891 ± 3.927	−1.101 ± 0.050	0.209 ± 0.059	–	
**Zn–5Mg**^**SPS**^		50	27.638 ± 4.833	−1.312 ± 0.012	0.427 ± 0.077	–	
**Zn**^**PM**^	SBF	14	–	−0.98	–	0.0137	[291]
**Zn-5Mg**^**PM**^		14	–	−1.42	–	0.0016	
**Zn-1Mg**^**HE**^		14	–	–	–	0.045	[294]
**Zn–1Mg-1β-TCP**^**HE**^		14	–	–	–	0.046	
**Zn–1Mg-0 TCP**^**HE**^		30	32.8	−1.177	0.491	–	[293]
**Zn–1Mg-1 TCP**^**HE**^		30	48.9	−1.225	0.732	–	
**Zn–1Mg-3 TCP**^**HE**^		30	59.6	−1.274	0.893	–	
**Zn–1Mg-5 TCP**^**HE**^		30	82.7	−1.335	1.239	–	
**Zn–2Mg-6SiC**^**PM**^		21	0.103	−0.581	0.025	–	[316]
**Zn**^**Cast**^	HBSS	28	0.81 ± 0.39	−0.966 ± 0.003	0.012 ± 0.007	–	[308]
**Zn-2Fe**^**Cast**^		28	0.82 ± 0.16	−0.959 ± 0.007	0.012 ± 0.002	–	
**Zn–2Fe-8 vol% WC**^**Cast**^		28	1.34 ± 0.50	−0.977 ± 0.022	0.020 ± 0.007	–	
**Zn**^**PM**^		60	–	–	–	0.77 mg/d	[314]
**Zn-1ND**^**PM**^		60	–	–	–	0.45 mg/d	
**Zn-2.5ND**^**PM**^		60	–	–	–	0.26 mg/d	

**Fig. 17:**
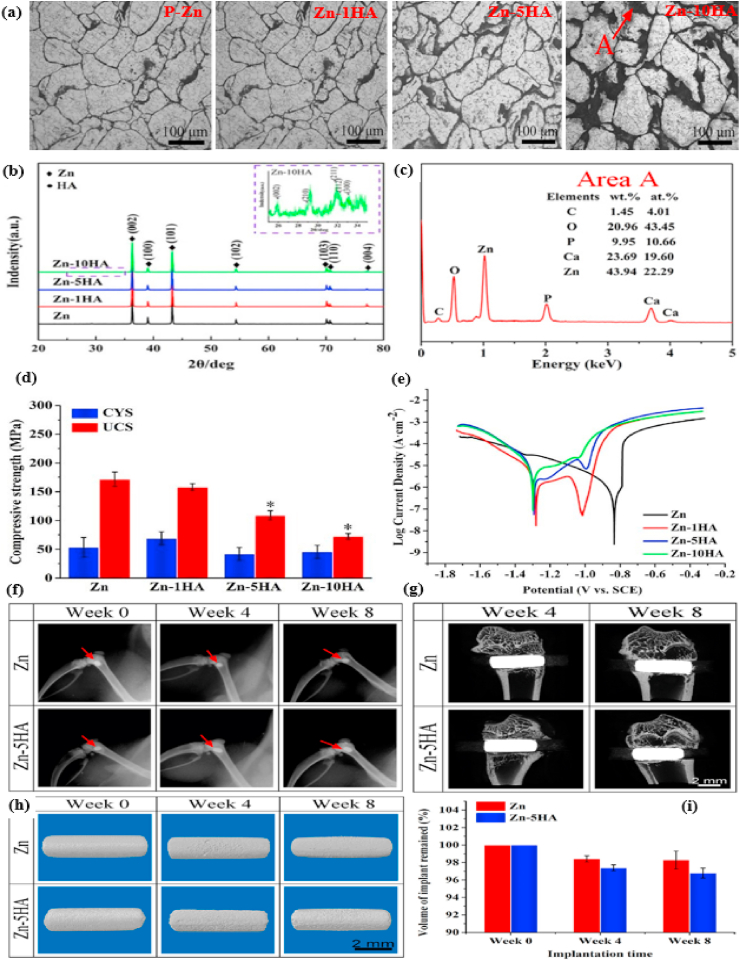
Microstructure, mechanical, and corrosion properties of Zn-xHA (x = 0, 1, 5, 10 wt%) composites, (a) optical micrographs showing the distribution of HA phase in Zn, (b) XRD pattern of Zn-HA composites with an inset showing the XRD peaks in angular range of 25^ᴼ^-35^ᴼ^, (c) EDX analysis of area A in (a), (d) compressive behavior of Zn-HA composites, and (e) polarization curves showing an electrochemical corrosion behavior of Zn-HA composites in HBSS; Micro-CT analysis. (f) Radiographs and (g) *in vivo* 2D images of implants. (h) In vivo 3D images, and (i) remaining volume of implants. (Reproduced with permission from Ref. [91]).

Yang et al. [91] fabricated Zn–(1, 5, 10 wt%) HA composites using the SPS technique and investigated their microstructure, mechanical properties, and *in vitro* degradation behaviors; their results are presented in Fig. 17. HA particles were mainly distributed along the grain boundaries of the Zn matrices (Fig. 17a), which was complemented by the X-ray diffraction and energy dispersive X-ray analysis results (Fig. 17b and c) which revealed peaks associated with HA. However, the addition of HA particles to Zn matrices did not enhance their mechanical and corrosion properties, as shown in Fig. 17d and e. Micro-CT analysis was performed on both the P–Zn and Zn-5HA composites to study the *in vivo* degradation behavior and new bone formation. No dislocation was found in any of the implants and no gas cavities were observed around the Zn and Zn-5HA composite implants (Fig. 17f). In contrast, cross-sections of femurs with the implants after 4 weeks showed formation of new bone in both samples and over time the amount of new bone mass increased surrounding the implants; however, in contrast to the matrix, the composite sample revealed better bone integration ability, i.e., direct and compact bone bonding was observed in the composite implant (Fig. 17g). Conversely, 3D reconstruction images of both implants showed a homogeneous and mild degradation progress (Fig. 17h) and both implants retained mechanical integrity up to 8 weeks’ implantation time, but the composite implant degraded slightly faster than its counterpart (pure–Zn), as shown in Fig. 17i. Recently, Pinc et al. [305] fabricated a Zn-8HA composite via extrusion and compared its properties with pure Zn. They reported that the addition of HA to pure Zn decreased the mechanical properties (σ_CYS_ and σ_UTS_) of the composite by almost 30%, although these levels are suitable for cancellous bone replacement.

**Fig. 18:**
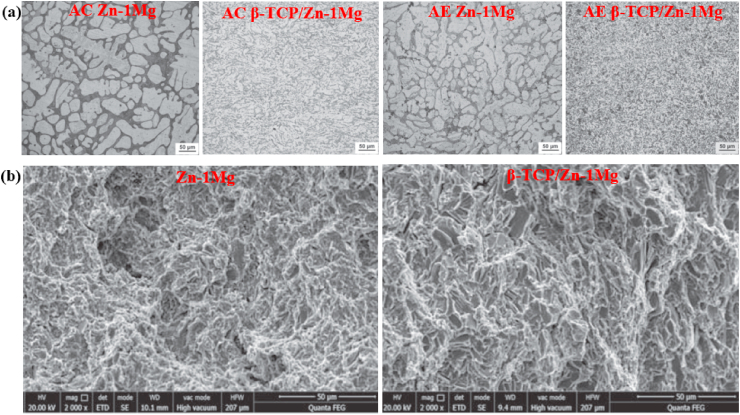
(a) Microstructure and (b) fracture surfaces after tensile test of Zn–1Mg alloy and β-TCP/Zn–1Mg composites. (Reproduced with permission from Ref. [294]).

Guoliang et al. [294] investigated the mechanical and corrosion behaviors of a bioabsorbable β-TCP/Zn–1Mg composite fabricated via HE. The microstructural and fracture behaviors of the composites are shown in Fig. 18. The addition of 1 vol% β-TCP particles to the Zn–1Mg matrix resulted in grain refinement of the Zn matrix (Fig. 18a) which significantly enhanced its tensile strength and ductility as compared to the unreinforced Zn–1Mg matrix. The fracture surfaces of the unreinforced Zn–1Mg matrix were primarily composed of cleavage facets with few dimples, whereas the fracture surfaces of the Zn matrices containing 1 vol% β-TCP revealed fewer cleavage facets and more dimpled areas (Fig. 18b), showing the better plasticity of this material under tensile loading. The *in vitro* degradation behavior of both the unreinforced Zn–Mg and the β-TCP/Zn–1Mg via immersion in SBD solution showed a steady corrosion rate of 0.05 mm/y; however, electrochemical testing of these materials revealed slightly higher corrosion rates in the composites than in the unreinforced Zn–1Mg matrix.

**Fig. 19:**
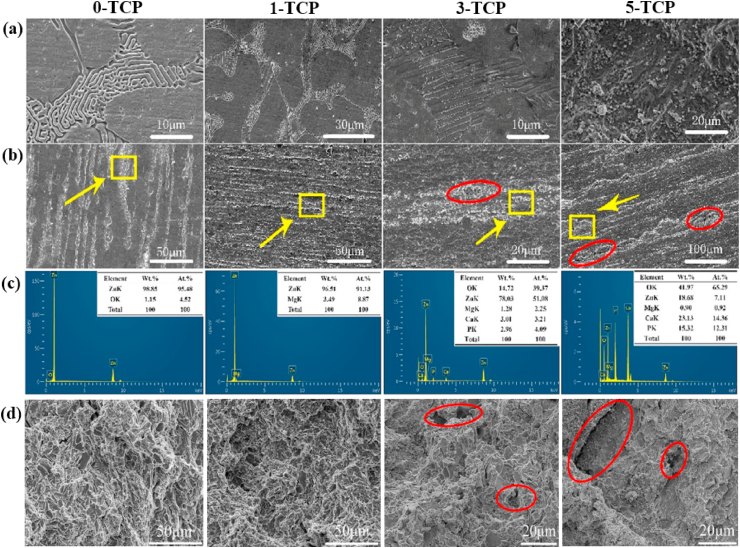
SEM images of as cast and extruded Zn–1Mg-x β-TCP (x = 0, 1, 3, 5 vol %) composites: (a) as-cast; (b) extruded; (c) EDX spectra of the yellow rectangular and red elliptical areas indicated in the extruded composites samples in (b); and (d) fracture surfaces of the Zn-based composites after tensile tests (The red elliptical areas show the hole defect appearing in the fractured surfaces). (Reproduced with permission from Refs. [293]).

**Fig. 20:**
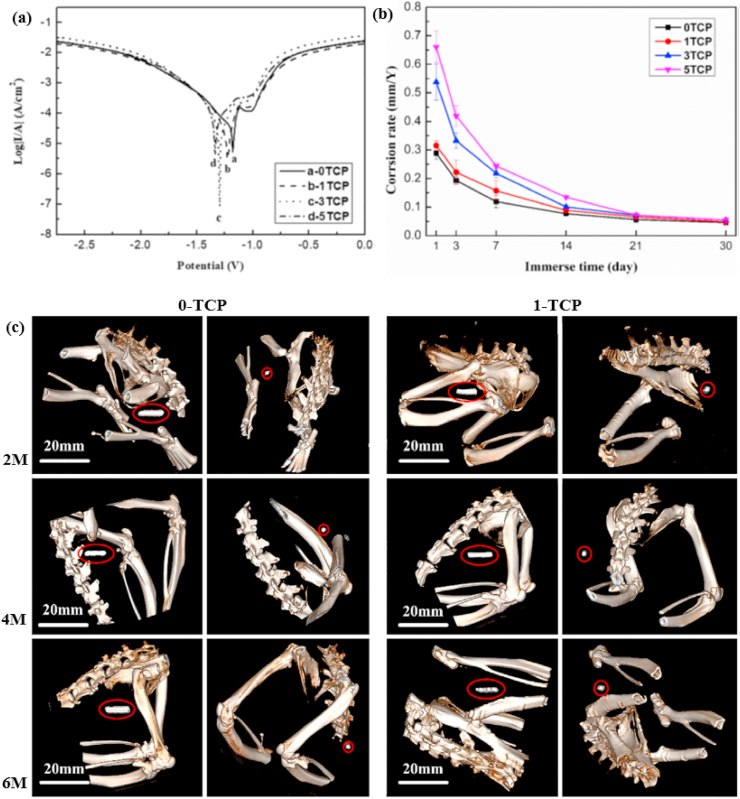
(a) Potentiodynamic polarization curves of Zn–1Mg-x β-TCP (x = 0, 1, 3, 5 vol %) composites soaked in SBF, (b) corrosion rates of Zn–1Mg-x β-TCP composites at different immersion durations in SBF, and (c) micro-CT 3D images of Zn–1Mg-x β-TCP composites after 2–6 months of implantation (The red ellipse areas show the horizontal and vertical position of the implant). (Reproduced with permission from Refs. [293]).

In another study by Pan et al. [293], the microstructures, mechanical properties, and degradation behaviors of Zn–1Mg-xβ-TCP (x = 0, 1, 3, 5 vol%) composites were investigated, and their microstructural and degradation properties are summarized, respectively, in Fig. 19, Fig. 20. The microstructures of the as-cast Zn–1Mg-xβ-TCP composites are shown in Fig. 19a and are primarily composed of a eutectic mixture of an α-Zn matrix and secondary phases such as Mg_2_Zn_11_, whereas the β-TCP particles were mainly distributed along the grain boundaries in the Zn–1Mg matrix, which contributed to dispersion strengthening in these composites. The EDS data (rectangular area pointed to by the yellow arrow) in the extruded micrographs indicated that less than 3 vol% β-TCP addition could not be detected (Fig. 19b and c). In contrast, excessive addition of β-TCP particles formed an agglomeration, indicated by the red ellipse in the extruded micrographs (Fig. 19b). The tensile test results for these composites revealed that their mechanical properties increased first and then reduced with increasing vol% of β-TCP (>1 vol%). Among all the composites, the Zn–1Mg-1 vol% β-TCP composite exhibited the best mechanical properties, as its values for σ_TYS_, σ_UTS_, ε, and E were measured at approximately 251 MPa, 331 MPa, 12%, and 125 GPa, respectively, which were reported to be suitable for orthopedic implant applications. The fracture morphology of the matrix contained cleavage fractures and a few dimples, while quasi-cleavage and microporous aggregate fractures were observed in the composite reinforced with 1 vol% β-TCP particles. Moreover, pore size gradually increased with increasing β-TCP particle content and adversely affected the mechanical properties (Fig. 19d). However, the *in vitro* C.R was increased by increasing the volume fraction of β-TCP particles in these composites (Fig. 20a), while the CRs of the four samples exhibited a similar nature with prolonged immersion time, as shown in Fig. 20b. The *in vivo* results obtained by micro-CT tomography showed that after 2 months' implantation, the surfaces of the implants slightly corroded but the implants still possessed their entire morphology, indicating good mechanical integrity of the implant after 2 months’ implantation (Fig. 20c). After 4 months, the edge of the implant was observed to be rougher as compared to 2 months. The surface of the composites containing 1 vol% TCP was slightly more corroded than the unreinforced matrix. In contrast, 6 months later the roughness on the edge of the Zn–1Mg-1β-TCP implant was even more evident; however, the degradation of the unreinforced Zn matrix (0-TCP) was more stable.

The potential of WC nanoparticles as a reinforcement material in Zn matrices for biodegradable implant applications were studied in Ref. [306,307]. The addition of WC nanoparticles (0–10 vol%) to Zn matrices improved the hardness of the monolithic Zn without adversely affecting the CR. The evaluation of the biodegradation displayed that the WC nanoparticles did not impact the release rate of Zn ions, and no detectable levels of tungsten ions were released from any of the nanocomposites [306]. The microwires fabricated from Zn–10 vol% WC nanocomposite could be used for stent weaving application [307]. The same research group in another study reported the mechanical and corrosion properties of WC reinforced Zn–2Fe based nanocomposites. With addition of 8 vol% WC in as-cast Zn–2Fe had improved ductility from 1.4 to 8.6%, while maintaining high mechanical strength. Corrosion test results confirmed that the suitable CR of Zn–2Fe was not impacted by the addition of WC nanoparticles [308]. Recently, Guan et al. [309] fabricated Zn–3TiB_2_ nanocomposite via ultrasound processing and hot rolling. With 3 vol% TiB_2_ nanoparticles, the mechanical strength of zinc has been significantly enhanced, e.g., H, σ_TYS_ and σ_UTS_, by 85, 90 and 45%, respectively, while ε retained 23% indicating the it as promising candidate for biodegradable medical devices.

### 6.2 Carbonaceous reinforcements in Zn-based composites

In recent years, various carbonaceous materials such as carbon nanotubes (CNTs) and graphene have also been utilized as reinforcing particulate materials in metallic biomaterials [317]. The mechanical properties such as strength, ductility, and fracture mode of fabricated metal matrix composites (MMCs) strongly depend upon the size and dispersion of these reinforcing particulates in the metal matrices [[318], [319], [320]]. Studies have elucidated that the strength of MMCs can be enhanced by the addition of uniformly distributed nanoscale carbonaceous particulates to the metal matrices. Carbon nanomaterials, such as single-walled carbon nanotubes (SWCNTs), multi-walled carbon nanotubes (MWCNTs), and graphene nanoplatelets (GNPs), exhibit great potential as nano-reinforcing materials in MMCs because of their high surface areas, extraordinary mechanical strength, and chemical stability [[321], [322], [323]]. Table 11 summarizes the characteristics and mechanical properties of various carbonaceous reinforcement materials.

**Table 11:** Mechanical properties of various carbonaceous reinforcements.

Materials	**Diameter (nm)**	Aspect ratio	Tensile Strength (GPa)	**E (GPa)**	**ε (%)**	Ref.
**SWCNT**	1–2	100-10,000	–	1000	–	[324]
**MWCNT**	5–50	100-10,000	150	270–950	12	[325,326]
**Graphene**	–	1000–10000	130	1000	–	[327,328]

CNTs are an allotropic form of C with a tubular morphology that is composed of a single layers of C atoms, and can be single-walled (SWCNT) with a diameter <1 nm or multi-walled (MWCNT), containing several concentrically interconnected nanotubes with diameters ≥100 nm [329]. These nanoscale materials exhibit remarkable mechanical strength (117% stronger than steel) and chemical stability. These carbonaceous materials have not only found potential applications in various advanced engineering applications such as field emitters, Li-ion batteries, and electrical contact materials, but have also gained the attention of the scientific community as they exhibit immense potential for biomedical applications including hard-tissue implants, scaffolds, and nanoscale biosensors [[330], [331], [332], [333]]. Similar to CNTs, graphene (with single-layer sp^2^-hybridized C atoms) is another fascinating and unique nanomaterial which demonstrates 2D properties such as superb mechanical, thermal, and electrical properties [334,335]. Extensive research has been carried out in recent years to exploit its unique mechanical and biological properties in a variety of applications since its discovery; however, research on its clinical applications is not still sufficiently reported [317,321,[336], [337], [338]]. At present, low-content MWCNTs and GNPs are used as reinforcing agents in biodegradable MMCs for biomedical applications [322,337,[339], [340], [341], [342], [343], [344]]. Yu et al. [314] studied PM-processed Zn–nanodiamond (ND) composites and reported that the grain size of the composites increased with higher ND content, causing reduced mechanical properties of the composites; however, compared to pure Zn, the composites displayed higher corrosion resistance in SBF solution. The mechanical and corrosion properties of all Zn–ND composites are summarized in Table 9, Table 10. Recently, Cu-coated MWCNTs that reinforced a Zn MMC (MWCNTs/Zn) were fabricated by Hongmei et al. [315] using a combination of electroless deposition (ED), SPS, and HR techniques. They reported that the mechanical properties of the composites gradually increased as the content of the MWCNTs increased and the 3 vol% Cu-coated MWCNTs/Zn composite displayed the maximum UTS (281 MPa), but its elongation was only 4%. Two key strengthening mechanisms in the composites were load transfer and grain refining effects. However, to date, no report has been found on biodegradable Zn-based composites reinforced using GNPs.

## 7 Summary and future directions

Zn-based BMs have already gained significant attention and are considered the next generation of biodegradable metallic biomaterials for clinical applications including tissue regeneration, bone implants, wound closure devices and cardiovascular stents. Extensive research has been carried out in recent years to investigate the properties of various Zn-based alloys and composites for biomedical applications. This study critically reviewed the current progress and challenges in the development of biodegradable Zn-based materials. An ideal biodegradable metallic implant should exhibit a suitable combination of biocompatibility, biodegradability and mechanical properties (σ_TYS_, σ_UTS_, and ε) for bone-tissue engineering. Although pure Zn exhibits good corrosion resistance in the physiological environment, its inadequate mechanical properties do not fulfil the requirements (σ_TYS_ > 200 MPa, σ_UTS_ > 300 MPa, and ε > 15–18%) for biodegradable implant applications. However, these widely accepted values were adapted from the specifications of permanent implant metal of 316L stainless steel (SS) stents. Typically, these values are applicable for permanent implants and may not be optimal for biodegradable metallic implants. Further, there are some commercially available temporary stents, which exhibit mechanical properties remarkably lower than those of 316L SS (e.g., poly-l-lactic acid (PLLA) with σ_UTS=_~70 MPa and ε = ~6%), yet are effective. Currently, nearly equivalent mechanical properties are desired for both vascular stents and bone implant devices, despite these distinct applications. Consequently, these phenomena justify the demand of independent re-evaluation of the mechanical requirements for biodegradable metallic devices.

Further, it is required to define standard application-driven specifications. However, since the current set of mechanical benchmarks requires further research into Zn-based materials, recent studies have suggested that these materials can fulfil the mechanical requirements by tailoring of their chemical compositions and special fabrication techniques followed by post thermomechanical processing. This review explained that the conventional metal forming processing of Zn-xMg alloys (x = 0.08–1.2 wt%) provides the best combination of mechanical properties, biocompatibility, and biodegradability for biodegradable implant applications. Recent developments on Zn–Li alloys demonstrated combination of excellent mechanical properties, suitable degradation behavior, and biocompatibility properties, indicating a suitability of these materials for biomedical applications.

Compared to Zn alloys containing various alloying elements, Zn-based composites have not been studied extensively, as only a few studies have reported a suitable combination of mechanical and corrosion properties for Zn-HA, Zn-MWCNTs and Zn–Mg–TCP composites. Zn-based implant materials have the potential to replace Mg-based and Fe-based alloys due to their good mechanical properties, biocompatibility and suitable degradation rate. A number of *in vitro* and *in vivo* studies on binary Zn–Mg, Zn–Ca, Zn–Sr and Zn–Li alloys have reported promising results for bone implant and vascular stent applications which are summarized in this review. Nevertheless, there are still several challenges and research gaps that need to be addressed before clinical trials of Zn-based materials.

The key conclusions from this study are as follows:

• The addition of new alloying elements should be assessed in Zn alloys in addition to the common elements of Mg, Ca, Sr, Mn, and Cu. Ti possesses good biocompatibility, and its alloys have been utilized in medical applications since the 1950s. A small amount of Ti addition can refine the grain size, thus can enhance the mechanical properties of Zn alloys.
• It is reported that the properties of biodegradable Zn-based alloys and composites heavily rely on the choice of fabrication techniques. Therefore, most of the studies have focused on investigating the effect of processing parameters of conventional processes on the mechanical and corrosion properties of Zn alloys. However, further research is required to investigate the biomechanical properties of Zn alloys processed by other advanced manufacturing techniques such as additive manufacturing. The best combination of strength and ductility in Zn alloys was achieved using SPD techniques such as hydrostatic extrusion. The AM or PM techniques have gained significant attention for fabrication of Mg-based alloys and composites, but they have not been fully explored for production of biodegradable Zn-based alloys and composites. However, in some cases, PM techniques followed by conventional processes such as extrusion, forging, and rolling have shown promising mechanical properties. Other advanced fabrication techniques, such as electron beam melting and electroforming are also feasible and could offer some distinct advantages. Thus, it would be interesting to pursue future studies to assess the capabilities of non-traditional processes on Zn alloys and composites for satisfying the requirements of biodegradable metal implants.
• Biodegradable implants are expected to maintain mechanical integrity during the healing process. Hence, time-dependent effects particularly age-hardening and strain-rate sensitivity should be evaluated in future investigations of absorbable Zn-based materials. Moreover, the data on dynamic properties such as corrosion-fatigue and creep, tribological properties, and natural aging for biodegradable Zn alloys and composites are currently insufficient. So, these properties should be investigated for better understanding of the loss of mechanical integrity during implant degradation and this is crucial for preventing implant's failure during service.
• The addition of nano-reinforcements (nano-diamond, CNTs, and graphene) can significantly improve the mechanical properties of Mg-based biomaterials. Thus, the effects of addition of nano-reinforcements in pure Zn and Zn alloys should be studied in future.
• Surface treatments, such as coating techniques can alter the properties of biodegradable metals. Biodegradable metal coatings, such as Zn-coated Mg or Fe on existing biodegradable metals can also be considered. Moreover, advanced surface treatments such as plasma surface engineering, magnetron sputtering, and electrochemical polymerisation could be used to alter the properties of monolithic Zn materials.
• Zn-based scaffolds should be explored for biodegradable implant applications, where materials require an open-cell porous structure mimicking that of cancellous bone. A few studies reported the fabrication of porous Zn-based implant materials via AM, SPS, and foam replication techniques and indicated promising results. Thus, it would be interesting to see the properties of Zn-based materials with an open-cell porous structure.
• It is important to understand the effect of physiological elements on corrosion of Zn-based materials. A thorough investigation of the impact of element (O), ions (Cl^−^, CO_3_^−^, HPO_4_^−^), and compounds (CO_2_) in the physiological environment on the degradation behavior of Zn-based materials could be beneficial to interpret the inconsistency between the *in vitro* and *in vivo* degradation rates.

This study reviewed the latest development in the fabrication of biodegradable Zn-based alloys and composites for biomedical implant applications. The processing techniques, metallurgical characteristics, microstructures, textures, wear and friction behaviors, and biomechanical and biodegradation properties have been described and discussed, along with their advantages and limitations.

## Declaration of competing interest

The authors declare that they have no known competing financial interests or personal relationships that could have appeared to influence the work reported in this paper.

SWCNT: Single-walled carbon nanotube; MWCNT: Multi-walled carbon nanotube.

## References

[1] Triclot P. (2010). Metal-on-metal: history, state of the art. Int. Orthop..

[2] Gotman I. (1997). Characteristics of metals used in implants. J. Endourol..

[3] Toong D.W.Y., Ng J.C.K., Huang Y., Wong P.E.H., Leo H.L., Venkatraman S.S., Ang H.Y. (2020). Bioresorbable metals in cardiovascular stents: material insights and progress. Materialia.

[4] Liu Y., Zheng Y., Chen X.H., Yang J.A., Pan H., Chen D., Wang L., Zhang J., Zhu D., Wu S., Yeung K.W.K., Zeng R.C., Han Y., Guan S. (2019). Fundamental theory of biodegradable metals—definition, criteria, and design. Adv. Funct. Mater..

[5] Witte F. (2010). The history of biodegradable magnesium implants: a review. Acta Biomater..

[6] Love B.J. (2017). Biomaterials: a Systems Approach to Engineering Concepts.

[7] Long M., Rack H.J. (1998). Titanium alloys in total joint replacement-a materials science perspective. Biomaterials.

[8] Muley S.V., Vidvans A.N., Chaudhari G.P., Udainiya S. (2016). An assessment of ultra fine grained 316L stainless steel for implant applications. Acta Biomater..

[9] Hinüber C., Kleemann C., Friederichs R.J., Haubold L., Scheibe H.J., Schuelke T., Boehlert C., Baumann M.J. (2010). Biocompatibility and mechanical properties of diamond‐like coatings on cobalt‐chromium‐molybdenum steel and titanium‐aluminum‐vanadium biomedical alloys. J. Biomed. Mater. Res..

[10] Patel B., Inam F., Reece M., Edirisinghe M., Bonfield W., Huang J., Angadji A. (2010). A novel route for processing cobalt-chromium-molybdenum orthopaedic alloys. J. R. Soc. Interface.

[11] Yoda K., Suyalatu, Takaichi A., Nomura N., Tsutsumi Y., Doi H., Kurosu S., Chiba A., Igarashi Y., Hanawa T. (2012). Effects of chromium and nitrogen content on the microstructures and mechanical properties of as-cast Co–Cr–Mo alloys for dental applications. Acta Biomater..

[12] Oliveira N.T.C., Guastaldi A.C. (2009). Electrochemical stability and corrosion resistance of Ti–Mo alloys for biomedical applications. Acta Biomater..

[13] Ehtemam-Haghighi S., Prashanth K.G., Attar H., Chaubey A.K., Cao G.H., Zhang L.C. (2016). Evaluation of mechanical and wear properties of TixNb7Fe alloys designed for biomedical applications. Mater. Des..

[14] Zhang S., Zhang X., Zhao C., Li J., Song Y., Xie C., Tao H., Zhang Y., He Y., Jiang Y., Bian Y. (2010). Research on an Mg–Zn alloy as a degradable biomaterial. Acta Biomater..

[15] Tahmasebifar A., Kayhan S.M., Evis Z., Tezcaner A., Çinici H., Koç M. (2016). Mechanical, electrochemical and biocompatibility evaluation of AZ91D magnesium alloy as a biomaterial. J. Alloys Compd..

[16] Saini M., Singh Y., Arora P., Arora V., Jain K. (2015). Implant biomaterials: a comprehensive review. World J. Clin. Cases.

[17] Manam N., Harun W., Shri D.N.A., Ghani S.A.C., Kurniawan T., Ismail M., Ibrahim M. (2017). Study of corrosion in biocompatible metals for implants: a review. J. Alloys Compd..

[18] Biesiekierski A., Wang J., Abdel-Hady Gepreel M., Wen C. (2012). A new look at biomedical Ti-based shape memory alloys. Acta Biomater..

[19] Witte F., Hort N., Vogt C., Cohen S., Kainer K., Willumeit R., Feyerabend F. (2008). Degradable biomaterials based on magnesium corrosion. Curr. Opin. Solid State Mater. Sci..

[20] Niinomi M. (2002). Recent metallic materials for biomedical applications. Metall. Mater. Trans..

[21] Li Y., Wen C., Mushahary D., Sravanthi R., Harishankar N., Pande G., Hodgson P. (2012). Mg–Zr–Sr alloys as biodegradable implant materials. Acta Biomater..

[22] Ali M., Hussein M.A., Al-Aqeeli N. (2019). Magnesium-based composites and alloys for medical applications: a review of mechanical and corrosion properties. J. Alloys Compd..

[23] Zhao D., Witte F., Lu F., Wang J., Li J., Qin L. (2017). Current status on clinical applications of magnesium-based orthopaedic implants: a review from clinical translational perspective. Biomaterials.

[24] Chaya A., Yoshizawa S., Verdelis K., Myers N., Costello B.J., Chou D.-T., Pal S., Maiti S., Kumta P.N., Sfeir C. (2015). In vivo study of magnesium plate and screw degradation and bone fracture healing. Acta Biomater..

[25] Heiden M., Walker E., Stanciu L. (2015). Biomaterials, Magnesium, iron and zinc alloys, the trifecta of bioresorbable orthopaedic and vascular implantation-a review. J. Biotechnol..

[26] Dewidar M., Yoon H.-C., Lim J. (2006). Mechanical properties of metals for biomedical applications using powder metallurgy process: a review. Met. Mater. Int..

[27] Zheng Y.F., Gu X.N., Witte F. (2014). Biodegradable metals. Mater. Sci. Eng. R Rep..

[28] Hermawan H. (2012). Biodegradable Metals: from Concept to Applications.

[29] Yin Z.-Z., Qi W.-C., Zeng R.-C., Chen X.-B., Gu C.-D., Guan S.-K., Zheng Y.-F. (2020). Advances in coatings on biodegradable magnesium alloys. J. Magnes. Alloy..

[30] Staiger M.P., Pietak A.M., Huadmai J., Dias G. (2006). Magnesium and its alloys as orthopedic biomaterials: a review. Biomaterials.

[31] Chen Q., Thouas G.A. (2015). Metallic implant biomaterials. Mater. Sci. Eng. R Rep..

[32] Dhillon M., Prabhakar S., Prasanna C. (2008). Preliminary experience with biodegradable implants for fracture fixation. Indian J. Orthop..

[33] Shao Y., Zeng R.-C., Li S.-Q., Cui L.-Y., Zou Y.-H., Guan S.-K., Zheng Y.-F. (2020). Advance in antibacterial magnesium alloys and surface coatings on magnesium alloys: a review. Acta Metall. Sin-Engl..

[34] Seitz J.M., Durisin M., Goldman J., Drelich J.W. (2015). Recent advances in biodegradable metals for medical sutures: a critical review. Adv. Healthc. Mater..

[35] Su Y., Wang Y., Tang L., Zheng Y., Qin Y.-X., Zhu D., Li B., Webster T. (2017). Development of biodegradable Zn-based medical implants. Orthopedic Biomaterials.

[36] Bowen P.K., Shearier E.R., Zhao S., Guillory R.J., Zhao F., Goldman J., Drelich J.W. (2016). Biodegradable metals for cardiovascular stents: from clinical concerns to recent Zn‐alloys. Ad. Healthc. Mater..

[37] Venezuela J., Dargusch M.S. (2019). The influence of alloying and fabrication techniques on the mechanical properties, biodegradability and biocompatibility of zinc: a comprehensive review. Acta Biomater..

[38] Hermawan H. (2018). Updates on the research and development of absorbable metals for biomedical applications. Prog. Biomater..

[39] Liu C., Lin G., Yang D., Qi M. (2006). In vitro corrosion behavior of multilayered Ti/TiN coating on biomedical AISI 316L stainless steel. Surf. Coating. Technol..

[40] Radha R., Sreekanth D. (2017). Insight of magnesium alloys and composites for orthopedic implant applications-a review. J. Magnes. Alloy..

[41] Li H.F., Shi Z.Z., Wang L.N. (2020). Opportunities and challenges of biodegradable Zn-based alloys. J. Mater. Sci. Technol..

[42] Shuai C., Li S., Peng S., Feng P., Lai Y., Gao C. (2019). Biodegradable metallic bone implants. Mater. Chem. Front..

[43] Virtanen S. (2011). Biodegradable Mg and Mg alloys: corrosion and biocompatibility. Mater. Sci. Eng. B.

[44] Chen Y., Xu Z., Smith C., Sankar J. (2014). Recent advances on the development of magnesium alloys for biodegradable implants. Acta Biomater..

[45] Wegener B., Sievers B., Utzschneider S., Müller P., Jansson V., Rößler S., Nies B., Stephani G., Kieback B., Quadbeck P. (2011). Microstructure, cytotoxicity and corrosion of powder-metallurgical iron alloys for biodegradable bone replacement materials. Mater. Sci. Eng. B.

[46] Francis A., Yang Y., Virtanen S., Boccaccini A. (2015). Iron and iron-based alloys for temporary cardiovascular applications. J. Mater. Sci. Mater. Med..

[47] Munir K., Lin J., Wen C., Wright P.F.A., Li Y. (2020). Mechanical, corrosion, and biocompatibility properties of Mg-Zr-Sr-Sc alloys for biodegradable implant applications. Acta Biomater..

[48] Čapek J., Kubásek J., Pinc J., Drahokoupil J., Čavojský M., Vojtěch D. (2020). Extrusion of the biodegradable ZnMg0.8Ca0.2 alloy-The influence of extrusion parameters on microstructure and mechanical characteristics. J. Mech. Behav. Biomed. Mater..

[49] Hernández-Escobar D., Champagne S., Yilmazer H., Dikici B., Boehlert C.J., Hermawan H. (2019). Current status and perspectives of zinc-based absorbable alloys for biomedical applications. Acta Biomater..

[50] He J., Li D.-W., He F.-L., Liu Y.-Y., Liu Y.-L., Zhang C.-Y., Ren F., Ye Y.-J., Deng X.-D., Yin D.-C. (2020). A study of degradation behaviour and biocompatibility of Zn-Fe alloy prepared by electrodeposition. Mater. Sci. Eng. C.

[51] Song M.-S., Zeng R.-C., Ding Y.-F., Li R.W., Easton M., Cole I., Birbilis N., Chen X.-B. (2019). Recent advances in biodegradation controls over Mg alloys for bone fracture management: a review. J. Mater. Sci. Technol..

[52] Agarwal S., Curtin J., Duffy B., Jaiswal S. (2016). Biodegradable magnesium alloys for orthopaedic applications: a review on corrosion, biocompatibility and surface modifications. Mater. Sci. Eng. C.

[53] Yang J., Koons G.L., Cheng G., Zhao L., Mikos A.G., Cui F. (2018). A review on the exploitation of biodegradable magnesium-based composites for medical applications. Biomed. Mater..

[54] Tam M., Gómez S., González-Gross M., Marcos A. (2003). Possible roles of magnesium on the immune system. Eur. J. Clin. Nutr..

[55] Li H., Yang H., Zheng Y., Zhou F., Qiu K., Wang X. (2015). Design and characterizations of novel biodegradable ternary Zn-based alloys with IIA nutrient alloying elements Mg, Ca and Sr. Mater. Des..

[56] Sezer N., Evis Z., Kayhan S.M., Tahmasebifar A., Koç M. (2018). Review of magnesium-based biomaterials and their applications. J. Magnes. Alloy..

[57] Li L.-Y., Cui L.-Y., Zeng R.-C., Li S.-Q., Chen X.-B., Zheng Y., Kannan M.B. (2018). Advances in functionalized polymer coatings on biodegradable magnesium alloys – a review. Acta Biomater..

[58] Ding Z.-Y., Cui L.-Y., Zeng R.-C., Zhao Y.-B., Guan S.-K., Xu D.-K., Lin C.-G. (2018). Exfoliation corrosion of extruded Mg-Li-Ca alloy. J. Mater. Sci. Technol..

[59] Atrens A., Liu M., Zainal Abidin N.I. (2011). Corrosion mechanism applicable to biodegradable magnesium implants. Mater. Sci. Eng. B.

[60] Sanchez A.H.M., Luthringer B.J.C., Feyerabend F., Willumeit R. (2015). Mg and Mg alloys: how comparable are in vitro and in vivo corrosion rates? A review. Acta Biomater..

[61] Atrens A., Song G.L., Liu M., Shi Z., Cao F., Dargusch M.S. (2015). Review of recent developments in the field of magnesium corrosion. Adv. Eng. Mater..

[62] Cao F., Song G.-L., Atrens A. (2016). Corrosion and passivation of magnesium alloys. Corrosion Sci..

[63] Zivic F., Grujovic N., Pellicer E., Sort J., Mitrovic S., Adamovic D., Vulovic M., Zivic F., Affatato S., Trajanovic M., Schnabelrauch M., Grujovic N., Choy K.L. (2018). Biodegradable metals as biomaterials for clinical practice: iron-Based materials. Biomaterials in Clinical Practice : Advances in Clinical Research and Medical Devices.

[64] Schinhammer M., Hänzi A.C., Löffler J.F., Uggowitzer P.J. (2010). Design strategy for biodegradable Fe-based alloys for medical applications. Acta Biomater..

[65] Zhu D., Su Y., Young M.L., Ma J., Zheng Y., Tang L. (2017). Biological responses and mechanisms of human bone marrow mesenchymal stem cells to Zn and Mg biomaterials. ACS Appl. Mater. Interfaces.

[66] Cheng J., Liu B., Wu Y.H., Zheng Y.F. (2013). Comparative invitro study on pure metals (Fe, Mn, Mg, Zn and W) as biodegradable metals. J. Mater. Sci. Technol..

[67] Peuster M., Hesse C., Schloo T., Fink C., Beerbaum P., von Schnakenburg C.J.B. (2006). Long-term biocompatibility of a corrodible peripheral iron stent in the porcine descending aorta. Biomaterials.

[68] Dambatta M.S., Kurniawan D., Izman S., Yahaya B., Hermawan H. (2015). Review on Zn-based alloys as potential biodegradable medical devices materials. Appl. Mech. Mater..

[69] Ma J., Zhao N., Zhu D. (2015). Endothelial cellular responses to biodegradable metal zinc. ACS Biomater. Sci. Eng..

[70] Ulum M.F., Arafat A., Noviana D., Yusop A.H., Nasution A.K., Abdul Kadir M.R., Hermawan H. (2014). In vitro and in vivo degradation evaluation of novel iron-bioceramic composites for bone implant applications. Mater. Sci. Eng. C.

[71] Liu X., Sun J., Yang Y., Pu Z., Zheng Y. (2015). In vitro investigation of ultra-pure Zn and its mini-tube as potential bioabsorbable stent material. Mater. Lett..

[72] Zhao L., Wang X., Wang T., Xia Y., Cui C. (2019). Mechanical properties and biodegradation of porous Zn-1Al alloy scaffolds. Mater. Lett..

[73] Yang D., Chen J., Chen W., Wang L., Wang H., Jiang J., Ma A. (2017). Fabrication of cellular Zn–Mg alloy foam by gas release reaction via powder metallurgical approach. J. Mater. Sci. Technol..

[74] Tong X., Shi Z., Xu L., Lin J., Zhang D., Wang K., Li Y., Wen C. (2020). Degradation behavior, cytotoxicity, hemolysis, and antibacterial properties of electro-deposited Zn–Cu metal foams as potential biodegradable bone implants. Acta Biomater..

[75] Li Y., Pavanram P., Zhou J., Lietaert K., Taheri P., Li W., San H., Leeflang M.A., Mol J.M.C., Jahr H., Zadpoor A.A. (2020). Additively manufactured biodegradable porous zinc. Acta Biomater..

[76] Li Y., Pavanram P., Zhou J., Lietaert K., Bobbert F.S.L., Kubo Y., Leeflang M.A., Jahr H., Zadpoor A.A. (2020). Additively manufactured functionally graded biodegradable porous zinc. Biomater. Sci..

[77] Cockerill I., Su Y., Sinha S., Qin Y.-X., Zheng Y., Young M.L., Zhu D. (2020). Porous zinc scaffolds for bone tissue engineering applications: a novel additive manufacturing and casting approach. Mater. Sci. Eng. C.

[78] Bowen P.K., Drelich J., Goldman J. (2013). Zinc exhibits ideal physiological corrosion behavior for bioabsorbable stents. Adv. Mater..

[79] Levy G., Goldman J., Aghion E. (2017). The prospects of zinc as a structural material for biodegradable implants-a review paper. Metals.

[80] Li G., Yang H., Zheng Y., Chen X.-H., Yang J.-A., Zhu D., Ruan L., Takashima K. (2019). Challenges in the use of zinc and its alloys as biodegradable metals: perspective from biomechanical compatibility. Acta Biomater..

[81] Christopher J.F., Jae-Young K., Ashley I.B. (2005). The neurobiology of zinc in health and disease. Nat. Rev. Neurosci..

[82] Plum L.M., Rink L., Haase H. (2010). The essential toxin: impact of zinc on human health. Int. J. Environ. Res. Publ. Health.

[83] Yue R., Niu J., Li Y., Ke G., Huang H., Pei J., Ding W., Yuan G. (2020). In vitro cytocompatibility, hemocompatibility and antibacterial properties of biodegradable Zn-Cu-Fe alloys for cardiovascular stents applications. Mater. Sci. Eng. C.

[84] Jimnez M., Abradelo C., San Romn J., Rojo L. (2019). Bibliographic review on the state of the art of strontium and zinc based regenerative therapies. Recent developments and clinical applications. J. Mater. Chem. B.

[85] Venezuela J.J.D., Johnston S., Dargusch M.S. (2019). The prospects for biodegradable zinc in wound closure applications. Adv. Healthc. Mater..

[86] Amano H., Miyake K., Hinoki A., Yokota K., Kinoshita F., Nakazawa A., Tanaka Y., Seto Y., Uchida H. (2020). Novel zinc alloys for biodegradable surgical staples. World J. Clin. Cases.

[87] Wang X., Shao X., Dai T., Xu F., Zhou J.G., Qu G., Tian L., Liu B., Liu Y. (2019). In vivo study of the efficacy, biosafety, and degradation of a zinc alloy osteosynthesis system. Acta Biomater..

[88] Su Y., Cockerill I., Wang Y., Qin Y.-X., Chang L., Zheng Y., Zhu D. (2019). Zinc-based biomaterials for regeneration and therapy. Trends Biotechnol..

[89] Mostaed E., Sikora-Jasinska M., Drelich J.W., Vedani M. (2018). Zinc-based alloys for degradable vascular stent applications. Acta Biomater..

[90] Yang H., Wang C., Liu C., Chen H., Wu Y., Han J., Jia Z., Lin W., Zhang D., Li W., Yuan W., Guo H., Li H., Yang G., Kong D., Zhu D., Takashima K., Ruan L., Nie J., Li X., Zheng Y. (2017). Evolution of the degradation mechanism of pure zinc stent in the one-year study of rabbit abdominal aorta model. Biomaterials.

[91] Yang H., Qu X., Lin W., Wang C., Zhu D., Dai K., Zheng Y. (2018). In vitro and in vivo studies on zinc-hydroxyapatite composites as novel biodegradable metal matrix composite for orthopedic applications. Acta Biomater..

[92] Bian D., Deng J., Li N., Chu X., Liu Y., Li W., Cai H., Xiu P., Zhang Y., Guan Z., Zheng Y., Kou Y., Jiang B., Chen R. (2018). In vitro and in vivo studies on biomedical magnesium low-alloying with elements gadolinium and zinc for orthopedic implant applications. ACS Appl. Mater. Interfaces.

[93] Chou J., Komuro M., Hao J., Kuroda S., Hattori Y., Ben‐Nissan B., Milthorpe B., Otsuka M. (2016). Bioresorbable zinc hydroxyapatite guided bone regeneration membrane for bone regeneration. Clin. Oral Implants Res..

[94] Vojtech D., Kubasek J., Serak J., Novak P. (2011). Mechanical and corrosion properties of newly developed biodegradable Zn-based alloys for bone fixation. Acta Biomater..

[95] Black J., Hastings G. (1998). Handbook of Biomaterial Properties.

[96] Giesen E.B.W., Ding M., Dalstra M., van Eijden T.M.G.J. (2001). Mechanical properties of cancellous bone in the human mandibular condyle are anisotropic. J. Biomech..

[97] Chen J., Tan L., Yu X., Etim I.P., Ibrahim M., Yang K. (2018). Mechanical properties of magnesium alloys for medical application: a review. J. Mech. Behav. Biomed. Mater..

[98] Niinomi M. (1998). Mechanical properties of biomedical titanium alloys. Mater. Sci. Eng., A.

[99] Mani G., Feldman M.D., Patel D., Agrawal C.M. (2007). Coronary stents: a materials perspective. Biomaterials.

[100] Čapek J., Jablonská E., Lipov J., Kubatík T.F., Vojtěch D. (2018). Preparation and characterization of porous zinc prepared by spark plasma sintering as a material for biodegradable scaffolds. Mater. Chem. Phys..

[101] Zhao S., Seitz J.-M., Eifler R., Maier H.J., Guillory R.J., Earley E.J., Drelich A., Goldman J., Drelich J.W. (2017). Zn-Li alloy after extrusion and drawing: structural, mechanical characterization, and biodegradation in abdominal aorta of rat. Mater. Sci. Eng. C.

[102] Sikora-Jasinska M., Mostaed E., Mostaed A., Beanland R., Mantovani D., Vedani M. (2017). Fabrication, mechanical properties and in vitro degradation behavior of newly developed Zn-Ag alloys for degradable implant applications. Mater. Sci. Eng. C.

[103] Zhao S., McNamara C.T., Bowen P.K., Verhun N., Braykovich J.P., Goldman J., Drelich J.W. (2017). Structural characteristics and in vitro biodegradation of a novel Zn-Li alloy prepared by induction melting and hot rolling. Metall. Mater. Trans..

[104] Gong H., Wang K., Strich R., Zhou J.G. (2015). In vitro biodegradation behavior, mechanical properties, and cytotoxicity of biodegradable Zn–Mg alloy. J. Biomed. Mater. Res. B.

[105] Yue R., Huang H., Ke G., Zhang H., Pei J., Xue G., Yuan G. (2017). Microstructure, mechanical properties and in vitro degradation behavior of novel Zn-Cu-Fe alloys. Mater. Char..

[106] Ehsan M., Malgorzata S.-J., Ali M., Diego M., Richard B., Maurizio V. (2016). Characterization of novel Zn-based alloys for biodegradable stent application. Front. Bioeng. Biotech. 10th World Biomaterials Congress.

[107] Katarivas Levy G., Leon A., Kafri A., Ventura Y., Drelich J., Goldman J., Vago R., Aghion E. (2017). Evaluation of biodegradable Zn-1%Mg and Zn-1%Mg-0.5%Ca alloys for biomedical applications. J. Mater. Sci. Mater. Med..

[108] Ghomashchi M.R., Vikhrov A. (2000). Squeeze casting: an overview. J. Mater. Process. Technol..

[109] Çay F., Can Kurnaz S. (2005). Hot tensile and fatigue behaviour of zinc–aluminum alloys produced by gravity and squeeze casting. Mater. Des..

[110] Vida T.A., Conde A., Freitas E.S., Arenas M.A., Cheung N., Brito C., de Damborenea J., Garcia A. (2017). Directionally solidified dilute Zn-Mg alloys: correlation between microstructure and corrosion properties. J. Alloys Compd..

[111] Verissimo N., Freitas E., Cheung N., Garcia A., Osório W. (2017). The effects of Zn segregation and microstructure length scale on the corrosion behavior of a directionally solidified Mg-25 wt.%Zn alloy. J. Alloys Compd..

[112] Verissimo N.C., Brito C., Santos W.L.R., Cheung N., Spinelli J.E., Garcia A. (2016). Interconnection of Zn content, macrosegregation, dendritic growth, nature of intermetallics and hardness in directionally solidified Mg–Zn alloys. J. Alloys Compd..

[113] Santos W.L.R., Cruz C.B., Spinelli J.E., Cheung N., Garcia A. (2018). Tailoring microstructure, tensile properties and fracture process via transient directional solidification of Zn-Sn alloys. Mater. Sci. Eng., A.

[114] Guleryuz L., Ipek R., Arıtman I., Karaoglu S. (2017). Microstructure and Mechanical Properties of Zn-Mg Alloys as Implant Materials Manufactured by Powder Metallurgy method AIP Conference Proceedings.

[115] Miranda-Hernández J.G., Herrera-Hernández H., González-Morán C.O., Rivera Olvera J.N., Estrada-Guel I., Botello Villa F. (2017). Synthesis and characterization of Zn-Ni advanced alloys prepared by mechanical milling and sintering at solid-state process. Ann. Mater. Sci. Eng..

[116] Verma R., Srinivasan A., Nath S.K., Jayaganthan R. (2019). Tensile and fracture toughness behaviour of ultrafine grained Mg-4Zn-4Gd alloy processed through hot rolling followed by hot pressing. Mater. Sci. Eng., A.

[117] Krystýnova M., Doležal P., Fintova S., Březina M., Zapletal J., Wasserbauer J. (2017). Preparation and characterization of zinc materials prepared by powder metallurgy. Metals.

[118] Sharma N., Alam S., Ray B., Cavaliere P. (2019). Fundamentals of spark plasma sintering (SPS): an ideal processing technique for fabrication of metal matrix nanocomposites. Spark Plasma Sintering of Materials.

[119] Munir Z., Anselmi-Tamburini U., Ohyanagi M. (2006). The effect of electric field and pressure on the synthesis and consolidation of materials: a review of the spark plasma sintering method. J. Mater. Sci..

[120] Rudinsky S., Hendrickx P., Bishop D.P., Brochu M. (2016). Spark plasma sintering and age hardening of an Al–Zn–Mg alloy powder blend. Mater. Sci. Eng., A.

[121] Bose S., Ke D., Sahasrabudhe H., Bandyopadhyay A. (2018). Additive manufacturing of biomaterials. Prog. Mater. Sci..

[122] Bandyopadhyay A., Heer B. (2018). Additive manufacturing of multi-material structures. Mater. Sci. Eng. R Rep..

[123] Wang X., Xu S., Zhou S., Xu W., Leary M., Choong P., Qian M., Brandt M., Xie Y.M. (2016). Topological design and additive manufacturing of porous metals for bone scaffolds and orthopaedic implants: a review. Biomaterials.

[124] Ngo T.D., Kashani A., Imbalzano G., Nguyen K.T.Q., Hui D. (2018). Additive manufacturing (3D printing): a review of materials, methods, applications and challenges. Compos. B Eng..

[125] Harun W.S.W., Kamariah M.S.I.N., Muhamad N., Ghani S.A.C., Ahmad F., Mohamed Z. (2018). A review of powder additive manufacturing processes for metallic biomaterials. Powder Technol..

[126] Aboulkhair N.T., Simonelli M., Salama E., Rance G.A., Neate N.C., Tuck C.J., Esawi A.M.K., Hague R.J.M. (2019). Evolution of carbon nanotubes and their metallurgical reactions in Al-based composites in response to laser irradiation during selective laser melting. Mater. Sci. Eng., A.

[127] Sova A., Smurov S., Grigoriev A., Okunkova I., Sova I. (2013). Potential of cold gas dynamic spray as additive manufacturing technology. Int. J. Adv. Manuf. Technol..

[128] Chen W., Thornley L., Coe H.G., Tonneslan S.J., Vericella J.J., Zhu C., Duoss E.B., Hunt R.M., Wight M.J., Apelian D., Pascall A.J., Kuntz J.D., Spadaccini C.M. (2017). Direct metal writing: controlling the rheology through microstructure. Appl. Phys. Lett..

[129] Matthews M.J., Guss G., Drachenberg D.R., Demuth J.A., Heebner J.E., Duoss E.B., Kuntz J.D., Spadaccini C.M. (2017). Diode-based additive manufacturing of metals using an optically-addressable light valve. Optic Express.

[130] Wen P., Jauer L., Voshage M., Chen Y., Poprawe R., Schleifenbaum J.H. (2018). Densification behavior of pure Zn metal parts produced by selective laser melting for manufacturing biodegradable implants. J. Mater. Process. Technol..

[131] Wen P., Qin Y., Chen Y., Voshage M., Jauer L., Poprawe R., Schleifenbaum J.H. (2019). Laser additive manufacturing of Zn porous scaffolds: shielding gas flow, surface quality and densification. J. Mater. Sci. Technol..

[132] Montani M., Demir A.G., Mostaed E., Vedani M., Previtali B. (2017). Processability of pure Zn and pure Fe by SLM for biodegradable metallic implant manufacturing. Rapid Prototyp. J..

[133] Demir A.G., Monguzzi L., Previtali B. (2017). Selective laser melting of pure Zn with high density for biodegradable implant manufacturing. Addit. Manuf..

[134] Galib R.H., Sharif A. (2015). Development of Zn-Mg alloys as a degradable biomaterial. Adv. Alloys Compd..

[135] Liu X., Sun J., Qiu K., Yang Y., Pu Z., Li L., Zheng Y. (2016). Effects of alloying elements (Ca and Sr) on microstructure, mechanical property and in vitro corrosion behavior of biodegradable Zn-1.5Mg alloy. J. Alloys Compd..

[136] Bakhsheshi-Rad H.R., Hamzah E., Low H.T., Kasiri-Asgarani M., Farahany S., Akbari E., Cho M.H. (2017). Fabrication of biodegradable Zn-Al-Mg alloy: mechanical properties, corrosion behavior, cytotoxicity and antibacterial activities. Mater. Sci. Eng. C.

[137] Vida T.A., Brito C., Lima T.S., Spinelli J.E., Cheung N., Garcia A. (2019). Near-eutectic Zn-Mg alloys: interrelations of solidification thermal parameters, microstructure length scale and tensile/corrosion properties. Curr. Appl. Phys..

[138] Yan Y., Liu H., Fang H., Yu K., Zhang T., Xu X., Zhang Y., Dai Y. (2018). Effects of the intermetallic phases on microstructure and properties of biodegradable magnesium matrix and zinc matrix prepared by powder metallurgy. Mater. Trans..

[139] Sotoudeh Bagha P., Khaleghpanah S., Sheibani S., Khakbiz M., Zakeri A. (2018). Characterization of nanostructured biodegradable Zn-Mn alloy synthesized by mechanical alloying. J. Alloys Compd..

[140] Wen P., Voshage M., Jauer L., Chen Y., Qin Y., Poprawe R., Schleifenbaum J.H. (2018). Laser additive manufacturing of Zn metal parts for biodegradable applications: processing, formation quality and mechanical properties,. Mater. Des..

[141] Shuai C., Xue L., Gao C., Yang Y., Peng S., Zhang Y. (2018). Selective laser melting of Zn-Ag alloys for bone repair: microstructure, mechanical properties and degradation behaviour. Virtual Phys. Prototyp..

[142] Yang Y., Yuan F., Gao C., Feng P., Xue L., He S., Shuai C. (2018). A combined strategy to enhance the properties of Zn by laser rapid solidification and laser alloying. J. Mech. Behav. Biomed. Mater..

[143] Shuai C., Cheng Y., Yang Y., Peng S., Yang W., Qi F. (2019). Laser additive manufacturing of Zn-2Al part for bone repair: formability, microstructure and properties. J. Alloys Compd..

[144] Kalpakjian S.A., Schmid S.R., Sekar K.S.V. (2016). Manufacturing : Engineering and Technology.

[145] Shi Z.-Z., Yu J., Liu X.-F., Wang L.-N. (2018). Fabrication and characterization of novel biodegradable Zn-Mn-Cu alloys. J. Mater. Sci. Technol..

[146] Bowen P.K., Seitz J.M., Guillory R.J., Braykovich J.P., Zhao S., Goldman J., Drelich J.W. (2018). Evaluation of wrought Zn-Al alloys (1, 3, and 5 wt % Al) through mechanical and in vivo testing for stent applications. J. Biomed. Mater. Res. B.

[147] Li H., Xie X., Zheng Y., Cong Y., Zhou F., Qiu K., Wang X., Chen S., Huang L., Tian L. (2015). Development of biodegradable Zn-1X binary alloys with nutrient alloying elements Mg, Ca and Sr. Sci. Rep..

[148] Xiao C., Wang L., Ren Y., Sun S., Zhang E., Yan C., Liu Q., Sun X., Shou F., Duan J., Wang H., Qin G. (2018). Indirectly extruded biodegradable Zn-0.05wt%Mg alloy with improved strength and ductility: in vitro and in vivo studies. J. Mater. Sci. Technol..

[149] Niu J., Tang Z., Huang H., Pei J., Zhang H., Yuan G., Ding W. (2016). Research on a Zn-Cu alloy as a biodegradable material for potential vascular stents application. Mater. Sci. Eng. C.

[150] Wang C., Yu Z., Cui Y., Zhang Y., Yu S., Qu G., Gong H. (2016). Processing of a novel Zn alloy micro-tube for biodegradable vascular stent application. J. Mater. Sci. Technol..

[151] Jin H., Zhao S., Guillory R., Bowen P.K., Yin Z., Griebel A., Schaffer J., Earley E.J., Goldman J., Drelich J.W. (2018). Novel high-strength, low-alloys Zn-Mg (< 0.1 wt% Mg) and their arterial biodegradation. Mater. Sci. Eng. C.

[152] Lin J., Tong X., Shi Z., Zhang D., Zhang L., Wang K., Wei A., Jin L., Lin J., Li Y., Wen C. (2020). A biodegradable Zn-1Cu-0.1Ti alloy with antibacterial properties for orthopedic applications. Acta Biomater..

[153] Shen C., Liu X., Fan B., Lan P., Zhou F., Li X., Wang H., Xiao X., Li L., Zhao S., Guo Z., Pu Z., Zheng Y. (2016). Mechanical properties, in vitro degradation behavior, hemocompatibility and cytotoxicity evaluation of Zn-1.2Mg alloy for biodegradable implants. RSC Adv..

[154] Jarzębska A., Bieda M., Kawałko J., Rogal Ł., Koprowski P., Sztwiertnia K., Pachla W., Kulczyk M. (2018). A new approach to plastic deformation of biodegradable zinc alloy with magnesium and its effect on microstructure and mechanical properties. Mater. Lett..

[155] Valiev R.Z., Langdon T.G. (2006). Principles of equal-channel angular pressing as a processing tool for grain refinement. Prog. Mater. Sci..

[156] Srinivasarao B., Zhilyaev A.P., Langdon T.G., Pérez-Prado M.T. (2013). On the relation between the microstructure and the mechanical behavior of pure Zn processed by high pressure torsion. Mater. Sci. Eng., A.

[157] Arab S.M., Akbarzadeh A. (2013). The effect of equal channel angular pressing process on the microstructure of AZ31 Mg alloy strip shaped specimens. J. Magnes. Alloy..

[158] Sitdikov O., Sakai T., Goloborodko A., Miura H., Kaibyshev R. (2004). Effect of pass strain on grain refinement in 7475 Al alloy during hot multidirectional forging. Mater. Trans..

[159] Edalati K., Horita Z. (2011). Significance of homologous temperature in softening behavior and grain size of pure metals processed by high-pressure torsion. Mater. Sci. Eng., A.

[160] Dambatta M.S., Izman S., Kurniawan D., Hermawan H. (2017). Processing of Zn-3Mg alloy by equal channel angular pressing for biodegradable metal implants. J. King Saud Univ. Sci..

[161] Hernández-Escobar D., Marcus J., Han J.-K., Unocic R.R., Kawasaki M., Boehlert C.J. (2020). Effect of post-deformation annealing on the microstructure and micro-mechanical behavior of Zn–Mg hybrids processed by High-Pressure Torsion. Mater. Sci. Eng., A.

[162] Tang Z., Niu J., Huang H., Zhang H., Pei J., Ou J., Yuan G. (2017). Potential biodegradable Zn-Cu binary alloys developed for cardiovascular implant applications. J. Mech. Behav. Biomed. Mater..

[163] Tang Z., Huang H., Niu J., Zhang L., Zhang H., Pei J., Tan J., Yuan G. (2017). Design and characterizations of novel biodegradable Zn-Cu-Mg alloys for potential biodegradable implants. Mater. Des..

[164] Kubásek J., Vojtěch D., Jablonská E., Pospíšilová I., Lipov J., Ruml T. (2016). Structure, mechanical characteristics and in vitro degradation, cytotoxicity, genotoxicity and mutagenicity of novel biodegradable Zn–Mg alloys. Mater. Sci. Eng. C.

[165] Lin S., Ran X., Yan X., Yan W., Wang Q., Yin T., Zhou J.G., Hu T., Wang G. (2019). Corrosion behavior and biocompatibility evaluation of a novel zinc‐based alloy stent in rabbit carotid artery model. J. Biomed. Mater. Res. B.

[166] Kang L., Zhai C.-P., Ming C., Lei L. (2018). Investigation on microstructures and mechanical properties of extruded biodegradable Zn-1Mg-xZr (x=0-0.4%) alloys. Proceedings of the International Conference on Computer, Communications and Mechatronics Engineering.

[167] Li P., Schille C., Schweizer E., Rupp F., Heiss A., Legner C., Klotz U.E., Geis-Gerstorfer J., Scheideler L. (2018). Mechanical characteristics, in vitro degradation, cytotoxicity, and antibacterial evaluation of Zn-4.0Ag alloy as a biodegradable material. Int. J. Mol. Sci..

[168] Tong X., Zhang D., Zhang X., Su Y., Shi Z., Wang K., Lin J., Li Y., Lin J., Wen C. (2018). Microstructure, mechanical properties, biocompatibility, and in vitro corrosion and degradation behavior of a new Zn–5Ge alloy for biodegradable implant materials. Acta Biomater..

[169] Shi Z.-Z., Li H.-Y., Xu J.-Y., Gao X.-X., Liu X.-F. (2020). Microstructure evolution of a high-strength low-alloy Zn–Mn–Ca alloy through casting, hot extrusion and warm caliber rolling. Mater. Sci. Eng., A.

[170] Bednarczyk W., Wątroba M., Kawałko J., Bała P. (2019). Can zinc alloys be strengthened by grain refinement? A critical evaluation of the processing of low-alloyed binary zinc alloys using ECAP. Mater. Sci. Eng., A.

[171] Bednarczyk W., Wątroba M., Kawałko J., Bała P. (2019). Determination of room-temperature superplastic asymmetry and anisotropy of Zn-0.8Ag alloy processed by ECAP. Mater. Sci. Eng., A.

[172] Demirtas M., Purcek G., Yanar H., Zhang Z.J., Zhang Z.F. (2015). Effect of equal-channel angular pressing on room temperature superplasticity of quasi-single phase Zn–0.3Al alloy. Mater. Sci. Eng., A.

[173] Bednarczyk W., Kawałko J., Wątroba M., Bała P. (2018). Achieving room temperature superplasticity in the Zn-0.5Cu alloy processed via equal channel angular pressing. Mater. Sci. Eng., A.

[174] Hernández-Escobar D., Rahman Z.U., Yilmazer H., Kawasaki M., Boehlert C.J. (2018). Microstructural evolution and intermetallic formation in Zn-Mg hybrids processed by high-pressure torsion. Philos. Mag. A.

[175] Bednarczyk W., Kawałko J., Wątroba M., Gao N., Starink M.J., Bała P., Langdon T.G. (2020). Microstructure and mechanical properties of a Zn-0.5Cu alloy processed by high-pressure torsion. Mater. Sci. Eng., A.

[176] Butts D.A., Gale W.F. (2003). Equilibrium diagrams. Smithells Met. Ref. B..

[177] Kubásek J., Pospíšilová I., Vojtěch D., Jablonská E., Ruml T. (2014). Structural, mechanical and cytotoxicity characterization of as-cast biodegradable Zn-xMg (x = 0.8-8.3 %) alloys. Mater. Tehnol..

[178] Mostaed E., Sikora-Jasinska M., Mostaed A., Loffredo S., Demir A.G., Previtali B., Mantovani D., Beanland R., Vedani M. (2016). Novel Zn-based alloys for biodegradable stent applications: design, development and in vitro degradation. J. Mech. Behav. Biomed. Mater..

[179] Ardakani M.S., Mostaed E., Sikora-Jasinska M., Kampe S.L., Drelich J.W. (2020). The effects of alloying with Cu and Mn and thermal treatments on the mechanical instability of Zn-0.05Mg alloy. Mater. Sci. Eng., A.

[180] Yang L., Guo P., Niu Z., Li F., Song Z., Xu C., Liu H., Sun W., Ren T. (2019). Influence of Mg on the mechanical properties and degradation performance of as-extruded Zn-Mg-Ca alloys: in vitro and in vivo behavior. J. Mech. Behav. Biomed. Mater..

[181] Zou Y., Chen X., Chen B. (2018). Effects of Ca concentration on degradation behavior of Zn-xCa alloys in Hank's solution. Mater. Lett..

[182] Zofková I., Nemcikova P., Matucha P. (2013). Trace elements and bone health. Clin. Chem. Lab. Med..

[183] Walsh P.M., O'Connor J.M., Strain J.J., New S., Bonjour J.P. (2003). The role of trace elements in bone health. Nutritional Aspects of Bone Health Cambridge.

[184] Bost M., Houdart S., Oberli M., Kalonji E., Huneau J.-F., Margaritis I. (2016). Dietary copper and human health: current evidence and unresolved issues. J. Trace Elem. Med. Biol..

[185] Harris E.D. (2004). A Requirement for copper in angiogenesis. Nutr. Rev..

[186] Uriu-Adams J.Y., Keen C.L. (2005). Copper, oxidative stress, and human health. Mol. Aspect. Med..

[187] Erikson K.M., Syversen T., Aschner J.L., Aschner M. (2005). Interactions between excessive manganese exposures and dietary iron-deficiency in neurodegeneration. Environ. Toxicol. Pharmacol..

[188] Sun S., Ren Y., Wang L., Yang B., Li H., Qin G. (2017). Abnormal effect of Mn addition on the mechanical properties of as-extruded Zn alloys. Mater. Sci. Eng., A.

[189] Shi Z.-Z., Yu J., Liu X.-F. (2018). Microalloyed Zn-Mn alloys: from extremely brittle to extraordinarily ductile at room temperature. Mater. Des..

[190] Guo P., Li F., Yang L., Bagheri R., Zhang Q., Li B.Q., Cho K., Song Z., Sun W., Liu H. (2019). Ultra-fine-grained Zn-0.5Mn alloy processed by multi-pass hot extrusion: grain refinement mechanism and room-temperature superplasticity. Mater. Sci. Eng., A.

[191] Shi Z.-Z., Yu J., Liu X.-F., Zhang H.-J., Zhang D.-W., Yin Y.-X., Wang L.-N. (2019). Effects of Ag, Cu or Ca addition on microstructure and comprehensive properties of biodegradable Zn-0.8Mn alloy. Mater. Sci. Eng. C.

[192] Shi Z.-Z., Gao X.-X., Chen H.-T., Liu X.-F., Li A., Zhang H.-J., Wang L.-N. (2020). Enhancement in mechanical and corrosion resistance properties of a biodegradable Zn-Fe alloy through second phase refinement. Mater. Sci. Eng. C.

[193] Dutkiewicz J. (1992). The Zn-Zr (zinc-zirconium) system. J. Phase Equil..

[194] Wątroba M., Bednarczyk W., Kawałko J., Bała P. (2018). Effect of zirconium microaddition on the microstructure and mechanical properties of Zn-Zr alloys. Mater. Char..

[195] Alexander J.W. (2009). History of the medical use of silver. Surg. Infect..

[196] Burdușel A.-C., Gherasim O., Grumezescu A.M., Mogoantă L., Ficai A., Andronescu E. (2018). Biomedical applications of silver nanoparticles: an up-to-date overview. Nanomaterials.

[197] Fordham W.R., Redmond S., Westerland A., Cortes E.G., Walker C., Gallagher C., Medina C.J., Waecther F., Lunk C., Ostrum R.F., Caputo G.A., Hettinger J.D., Krchnavek R.R. (2014). Silver as a bactericidal coating for biomedical implants. Surf. Coating. Technol..

[198] García‐Contreras R., Argueta‐Figueroa L., Mejía‐Rubalcava C., Jiménez‐Martínez R., Cuevas‐Guajardo S., Sánchez‐Reyna P.A., Mendieta‐Zeron H. (2011). Perspectives for the use of silver nanoparticles in dental practice. Int. Dent. J..

[199] Simchi A., Tamjid E., Pishbin F., Boccaccini A.R. (2011). Recent progress in inorganic and composite coatings with bactericidal capability for orthopaedic applications. Nanomed. Nanotechnol. Biol. Med..

[200] Xie Y., Zhao L., Zhang Z., Wang X., Wang R., Cui C. (2018). Fabrication and properties of porous Zn-Ag alloy scaffolds as biodegradable materials. Mater. Chem. Phys..

[201] Mirza A., King A., Troakes C., Exley C. (2017). Aluminium in brain tissue in familial Alzheimer's disease. J. Trace Elem. Med. Biol..

[202] Exley C., House E.R. (2011). Aluminium in the human brain. Monatsh. Chem..

[203] Homayun B., Afshar A. (2014). Microstructure, mechanical properties, corrosion behavior and cytotoxicity of Mg–Zn–Al–Ca alloys as biodegradable materials. J. Alloys Compd..

[204] Ratna Sunil B., Sampath Kumar T.S., Chakkingal U., Nandakumar V., Doble M., Devi Prasad V., Raghunath M. (2016). In vitro and in vivo studies of biodegradable fine grained AZ31 magnesium alloy produced by equal channel angular pressing. Mater. Sci. Eng. C.

[205] Bakhsheshi-Rad H.R., Hamzah E., Low H.T., Cho M.H., Kasiri-Asgarani M., Farahany S., Mostafa A., Medraj M. (2017). Thermal characteristics, mechanical properties, in vitro degradation and cytotoxicity of novel biodegradable Zn–Al–Mg and Zn–Al–Mg–xBi alloys. Acta Metall. Sin-Engl..

[206] Ferner R.E., Smith J.M., Koren G. (1992). Lithium and pregnancy. Lancet.

[207] Aral H., Vecchio-Sadus A. (2008). Toxicity of lithium to humans and the environment-A literature review. Ecotoxicol. Environ. Saf..

[208] Young W. (2009). Review of lithium effects on brain and blood. Cell Transplant..

[209] Liu Y., Wu Y., Bian D., Gao S., Leeflang S., Guo H., Zheng Y., Zhou J. (2017). Study on the Mg-Li-Zn ternary alloy system with improved mechanical properties, good degradation performance and different responses to cells. Acta Biomater..

[210] Pelton A.D. (1991). The Li-Zn (Lithium-Zinc) system. J. Phase Equil..

[211] Zhu S., Wu C., Li G., Zheng Y., Nie J.-F. (2020). Microstructure, mechanical properties and creep behaviour of extruded Zn-xLi (x = 0.1, 0.3 and 0.4) alloys for biodegradable vascular stent applications. Mater. Sci. Eng., A.

[212] Li Z., Shi Z.-Z., Hao Y., Li H.-F., Zhang H.-J., Liu X.-F., Wang L.-N. (2020). Insight into role and mechanism of Li on the key aspects of biodegradable Zn-Li alloys: microstructure evolution, mechanical properties, corrosion behavior and cytotoxicity. Mater. Sci. Eng. C.

[213] Yang H., Jia B., Zhang Z., Qu X., Li G., Lin W., Zhu D., Dai K., Zheng Y. (2020). Alloying design of biodegradable zinc as promising bone implants for load-bearing applications. Nat. Commun..

[214] Kubasek J., Vojtěch D. (2012). Zn-based alloys as an alternative biodegradable materials. J. Proc. Metal.

[215] Liu S., Kent D., Doan N., Dargusch M., Wang G. (2019). Effects of deformation twinning on the mechanical properties of biodegradable Zn-Mg alloys. Bioact. Mater..

[216] Zhu D., Cockerill I., Su Y., Zhang Z., Fu J., Lee K.-W., Ma J., Okpokwasili C., Tang L., Zheng Y., Qin Y.-X., Wang Y. (2019). Mechanical strength, biodegradation, and in vitro and in vivo biocompatibility of Zn biomaterials. ACS Appl. Mater. Interfaces.

[217] Lietaert K., Zadpoor A.A., Sonnaert M., Schrooten J., Weber L., Mortensen A., Vleugels J. (2020). Mechanical properties and cytocompatibility of dense and porous Zn produced by laser powder bed fusion for biodegradable implant applications. Acta Biomater..

[218] Wang L.-Q., Ren Y.-P., Sun S.-N., Zhao H., Li S., Qin G.-W. (2017). Microstructure, mechanical properties and fracture behavior of as-extruded Zn–Mg binary alloys. Acta Metall. Sin-Engl..

[219] Wang L., He Y., Zhao H., Xie H., Li S., Ren Y., Qin G. (2018). Effect of cumulative strain on the microstructural and mechanical properties of Zn-0.02 wt%Mg alloy wires during room-temperature drawing process. J. Alloys Compd..

[220] Shen C., Liu X., Fan B., Lan P., Zhou F., Li X., Wang H., Xiao X., Li L., Zhao S., Guo Z., Pu Z., Zheng Y. (2016). Mechanical properties, in vitro degradation behavior, hemocompatibility and cytotoxicity evaluation of Zn-1.2Mg alloy for biodegradable implants. RSC Adv..

[221] Kubásek J., Vojtěch D., Pospíšilová I., Michalcová A., Maixner J. (2016). Microstructure and mechanical properties of the micrograined hypoeutectic Zn–Mg alloy. Int. J. Miner. Metall. Mater..

[222] Dambatta M.S., Izman S., Kurniawan D., Farahany S., Yahaya B., Hermawan H. (2015). Influence of thermal treatment on microstructure, mechanical and degradation properties of Zn–3Mg alloy as potential biodegradable implant material. Mater. Des..

[223] Pospíšilová I., Soukupová V., Vojtěch D. (2017). Influence of calcium on the structure and mechanical properties of biodegradable zinc alloys. Mater. Sci. Forum.

[224] Lin S., Wang Q., Yan X., Ran X., Wang L., Zhou J.G., Hu T., Wang G. (2019). Mechanical properties, degradation behaviors and biocompatibility evaluation of a biodegradable Zn-Mg-Cu alloy for cardiovascular implants. Mater. Lett..

[225] Liu X., Sun J., Yang Y., Zhou F., Pu Z., Li L., Zheng Y. (2016). Microstructure, mechanical properties, in vitro degradation behavior and hemocompatibility of novel Zn–Mg–Sr alloys as biodegradable metals. Mater. Lett..

[226] Liu X., Sun J., Zhou F., Yang Y., Chang R., Qiu K., Pu Z., Li L., Zheng Y. (2016). Micro-alloying with Mn in Zn–Mg alloy for future biodegradable metals application. Mater. Des..

[227] Ren T., Gao X., Xu C., Yang L., Guo P., Liu H., Chen Y., Sun W., Song Z. (2019). Evaluation of as‐extruded ternary Zn–Mg–Zr alloys for biomedical implantation material: in vitro and in vivo behavior. Mater. Corros..

[228] Li P., Zhang W., Dai J., Xepapadeas A.B., Schweizer E., Alexander D., Scheideler L., Zhou C., Zhang H., Wan G., Geis-Gerstorfer J. (2019). Investigation of zinc-copper alloys as potential materials for craniomaxillofacial osteosynthesis implants. Mater. Sci. Eng. C.

[229] Zhang L., Liu X.Y., Huang H., Zhan W. (2019). Effects of Ti on microstructure, mechanical properties and biodegradation behavior of Zn-Cu alloy. Mater. Lett..

[230] Kafri A., Ovadia S., Goldman J., Drelich J., Aghion E. (2018). The suitability of Zn-1.3%Fe alloy as a biodegradable implant material. Metals.

[231] Jia B., Yang H., Han Y., Zhang Z., Qu X., Zhuang Y., Wu Q., Zheng Y., Dai K. (2020). In vitro and in vivo studies of Zn-Mn biodegradable metals designed for orthopedic applications. Acta Biomater..

[232] Shi Z.Z., Yu J., Liu X.F., Zhang H.J., Zhang D.W., Yin Y.X., Wang L.N. (2019). Effects of Ag, Cu or Ca addition on microstructure and comprehensive properties of biodegradable Zn-0.8Mn alloy. Mater. Sci. Eng. C.

[233] Chen H.-T., Shi Z.-Z., Liu X.-F. (2020). Microstructure and mechanical properties of extruded and caliber rolled biodegradable Zn-0.8Mn-0.4Ag alloy with high ductility. Mater. Sci. Eng., A.

[234] Shi Z.-Z., Li Z.-L., Bai W.-S., Tuoliken A., Yu J., Liu X.-F., Fe Mn (2019). Zn13 phase and its core-shell structure in novel biodegradable Zn-Mn-Fe alloys. Mater. Des..

[235] Mostaed E., Sikora-Jasinska M., Ardakani M.S., Mostaed A., Reaney I.M., Goldman J., Drelich J.W. (2020). Towards revealing key factors in mechanical instability of bioabsorbable Zn-based alloys for intended vascular stenting. Acta Biomater..

[236] Wang C., Yang H.T., Li X., Zheng Y.F. (2016). In vitro evaluation of the feasibility of commercial Zn alloys as biodegradable metals. J. Mater. Sci. Technol..

[237] Dai Y., Zhang Y., Liu H., Fang H., Li D., Xu X., Yan Y., Chen L., Lu Y., Yu K. (2019). Mechanical strengthening mechanism of Zn-Li alloy and its mini tube as potential absorbable stent material. Mater. Lett..

[238] Zhu S., Wu C., Li G., Zheng Y., Nie J.-F. (2019). Creep properties of biodegradable Zn-0.1Li alloy at human body temperature: implications for its durability as stents. Mater. Res. Lett..

[239] Zhang Y., Yan Y., Xu X., Lu Y., Chen L., Li D., Dai Y., Kang Y., Yu K. (2019). Investigation on the microstructure, mechanical properties, in vitro degradation behavior and biocompatibility of newly developed Zn-0.8%Li-(Mg, Ag) alloys for guided bone regeneration. Mater. Sci. Eng. C.

[240] Li Z., Shi Z.-Z., Hao Y., Li H.-F., Liu X.-F., Volinsky A.A., Zhang H.-J., Wang L.-N. (2019). High-performance hot-warm rolled Zn-0.8Li alloy with nano-sized metastable precipitates and sub-micron grains for biodegradable stents. J. Mater. Sci. Technol..

[241] Yin Z. (2017). Microstructural Evolution and Mechanical Properties of Zn-Ti Alloys for Biodegradable Stent Applications.

[242] Türk A., Kurnaz C., Şevik H. (2007). Comparison of the wear properties of modified ZA-8 alloys and conventional bearing bronze. Mater. Des..

[243] Prasad B.K., Patwardhan A.K., Yegneswaran A.H. (1996). Dry sliding wear characteristics of some zinc-aluminium alloys: a comparative study with a conventional bearing bronze at a slow speed. Wear.

[244] Savaşkan T., Maleki R.A. (2014). Friction and wear properties of Zn-25Al-based bearing alloys. Tribol. Trans..

[245] Pürçek G., Savaşkan T., Küçükömeroğlu T., Murphy S. (2002). Dry sliding friction and wear properties of zinc-based alloys. Wear.

[246] Pola A., Montesano L., Gelfi M., La Vecchia G.M. (2016). Comparison of the sliding wear of a novel Zn alloy with that of two commercial Zn alloys against bearing steel and leaded brass. Wear.

[247] Hanna M.D., Carter J.T., Rashid M.S. (1997). Sliding wear and friction characteristics of six Zn-based die-casting alloys. Wear.

[248] Ares A.E., Gassa L.M., Schvezov C.E., Rosenberger M.R. (2012). Corrosion and wear resistance of hypoeutectic Zn–Al alloys as a function of structural features. Mater. Chem. Phys..

[249] Abou El-khair M.T., Daoud A., Ismail A. (2004). Effect of different Al contents on the microstructure, tensile and wear properties of Zn-based alloy. Mater. Lett..

[250] Şevik H. (2014). The effect of silver on wear behaviour of zinc–aluminium-based ZA-12 alloy produced by gravity casting. Mater. Char..

[251] Savaşkan T., Pürçek G., Hekimoğlu A.P. (2003). Effect of copper content on the mechanical and tribological properties of ZnAl27-based alloys. Tribol. Lett..

[252] Lin J., Tong X., Wang K., Shi Z., Li Y., Dargusch M., Wen C. (2020). Biodegradable Zn–3Cu and Zn–3Cu–0.2Ti alloys with ultrahigh ductility and antibacterial ability for orthopedic applications. J. Mater. Sci. Technol..

[253] de La Fuente D., Castaño J.G., Morcillo M. (2007). Long-term atmospheric corrosion of zinc. Corrosion Sci..

[254] Li P., Schille C., Schweizer E., Kimmerle-Müller E., Rupp F., Heiss A., Legner C., Klotz U.E., Geis-Gerstorfer J., Scheideler L. (2019). Selection of extraction medium influences cytotoxicity of zinc and its alloys. Acta Biomater..

[255] Lin S., Ran X., Yan X., Wang Q., Zhou J.G., Hu T., Wang G. (2019). Systematical evolution on a Zn-Mg alloy potentially developed for biodegradable cardiovascular stents. J. Mater. Sci. Mater. Med..

[256] Törne K., Larsson M., Norlin A., Weissenrieder J. (2016). Degradation of zinc in saline solutions, plasma, and whole blood. J. Biomed. Mater. Res. B.

[257] Zhang X.G. (2013). Corrosion and Electrochemistry of Zinc.

[258] ASTM, ASTM (2014). G59-97 Standard Test Method for Conducting Potentiodynamic Polarization Resistance Measurements.

[259] ASTM, ASTM (2004). G31-72 standard practice for laboratory immersion corrosion testing of metals.

[260] Liu L., Gebresellasie K., Collins B., Zhang H., Xu Z., Young-Choon L. (2018). Degradation rates of pure zinc, magnesium, and magnesium alloys measured by volume loss, mass loss, and hydrogen evolution. Appl. Sci..

[261] Dambatta M.S., Murni N., Izman S., Kurniawan D., Froemming G., Hermawan H. (2015). In vitro degradation and cell viability assessment of Zn–3Mg alloy for biodegradable bone implants. Proc. Inst. Mech. Eng. H J. Eng. Med..

[262] Chen Y., Zhang W., Maitz M.F., Chen M., Zhang H., Mao J., Zhao Y., Huang N., Wan G. (2016). Comparative corrosion behavior of Zn with Fe and Mg in the course of immersion degradation in phosphate buffered saline. Corrosion Sci..

[263] Jablonská E., Vojtěch D., Fousová M., Kubásek J., Lipov J., Fojt J., Ruml T. (2016). Influence of surface pre-treatment on the cytocompatibility of a novel biodegradable Zn-Mg alloy. Mater. Sci. Eng. C.

[264] Liu L., Meng Y., Dong C., Yan Y., Volinsky A.A., Wang L.-N. (2018). Initial formation of corrosion products on pure zinc in simulated body fluid. J. Mater. Sci. Technol..

[265] Krężel A., Maret W. (2016). The biological inorganic chemistry of zinc ions. Arch. Biochem. Biophys..

[266] Thomas S., Birbilis N., Venkatraman M.S., Cole I.S. (2012). Corrosion.

[267] Liu L., Meng Y., Volinsky A.A., Zhang H.-J., Wang L.-N. (2019). Influences of albumin on in vitro corrosion of pure Zn in artificial plasma. Corrosion Sci..

[268] Guo H., Cao R.H., Zheng Y.F., Bai J., Xue F., Chu C.L. (2019). Diameter-dependent in vitro performance of biodegradable pure zinc wires for suture application. J. Mater. Sci. Technol..

[269] Meng Y., Liu L., Zhang D., Dong C., Yan Y., Volinsky A.A., Wang L.-N. (2019). Initial formation of corrosion products on pure zinc in saline solution. Bioact. Mater..

[270] Li N., Zheng Y. (2013). Novel magnesium alloys developed for biomedical application: a review. J. Mater. Sci. Technol..

[271] Hou Y., Jia G., Yue R., Chen C., Pei J., Zhang H., Huang H., Xiong M., Yuan G. (2018). Synthesis of biodegradable Zn-based scaffolds using NaCl templates: relationship between porosity, compressive properties and degradation behavior. Mater. Char..

[272] Kafri A., Ovadia S., Yosafovich-Doitch G., Aghion E. (2019). The effects of 4%Fe on the performance of pure zinc as biodegradable implant material. Ann. Biomed. Eng..

[273] Champagne S., Mostaed E., Safizadeh F., Ghali E., Vedani M., Hermawan H. (2019). In vitro degradation of absorbable zinc alloys in artificial urine. Materials.

[274] Fu J., Su Y., Qin Y.-X., Zheng Y., Wang Y., Zhu D. (2020). Evolution of metallic cardiovascular stent materials: a comparative study among stainless steel, magnesium and zinc. Biomaterials.

[275] Sheng Y., Zhou H., Li Z., Chen L., Wang X., Zhao X., Li W. (2019). Improved blood compatibility and cyto-compatibility of Zn-1Mg via plasma electrolytic oxidation. Materialia.

[276] Alves M.M., Proek T., Santos C.F., Montemor M.F. (2017). Evolution of the in vitro degradation of Zn-Mg alloys under simulated physiological conditions. RSC Adv..

[277] SathishKumar G., Parameswaran P., Vijayan V., Yokeswaran R. (2020). Effects of Ca, Cu concentration on degradation behavior of Zn alloys in Hank's solution. Met. Powder Rep..

[278] Shuai C., Dong Z., He C., Yang W., Peng S., Yang Y., Qi F. (2020). A peritectic phase refines the microstructure and enhances Zn implants. J. Mater. Res. Technol..

[279] Sun J., Zhang X., Shi Z.-Z., Gao X.-X., Liu X.-F., Wang J.-Q., Wang L.-N. (2020). Adjusting comprehensive properties of biodegradable Zn-Mn alloy through solution heat-treatment. Mater. Today Commun..

[280] Wątroba M., Bednarczyk W., Kawałko J., Mech K., Marciszko M., Boelter G., Banzhaf M., Bała P. (2019). Design of novel Zn-Ag-Zr alloy with enhanced strength as a potential biodegradable implant material. Mater. Des..

[281] Kannan M., Moore C., Saptarshi S., Somasundaram S., Rahuma M., Lopata A. (2017). Biocompatibility and biodegradation studies of a commercial zinc alloy for temporary mini-implant applications. Sci. Rep..

[282] Bowen P.K., Guillory R.J., Shearier E.R., Seitz J.-M., Drelich J., Bocks M., Zhao F., Goldman J. (2015). Metallic zinc exhibits optimal biocompatibility for bioabsorbable endovascular stents. Mater. Sci. Eng. C.

[283] Drelich A.J., Zhao S., Guillory R.J., Drelich J.W., Goldman J. (2017). Long-term surveillance of zinc implant in murine artery: surprisingly steady biocorrosion rate. Acta Biomater..

[284] Kafri A., Ovadia S., Yosafovich-Doitch G., Aghion E. (2018). In vivo performances of pure Zn and Zn–Fe alloy as biodegradable implants. J. Mater. Sci. Mater. Med..

[285] Zhou C., Li H.-F., Yin Y.-X., Shi Z.-Z., Li T., Feng X.-Y., Zhang J.-W., Song C.-X., Cui X.-S., Xu K.-L., Zhao Y.-W., Hou W.-B., Lu S.-T., Liu G., Li M.-Q., Ma J.-Y., Toft E., Volinsky A.A., Wan M., Yao X.-J. (2019). Long-term in vivo study of biodegradable Zn-Cu stent: a 2-year implantation evaluation in porcine coronary artery. Acta Biomater..

[286] Shearier E.R., Bowen P.K., He W., Drelich A., Drelich J., Goldman J., Zhao F. (2016). In vitro cytotoxicity, adhesion, and proliferation of human vascular cells exposed to zinc. ACS Biomater. Sci. Eng..

[287] Pierson D., Edick J., Tauscher A., Pokorney E., Bowen P., Gelbaugh J., Stinson J., Getty H., Lee C.H., Drelich J., Goldman J. (2012). A simplified in vivo approach for evaluating the bioabsorbable behavior of candidate stent materials. J. Biomed. Mater. Res. B.

[288] Guillory R.J., Bowen P.K., Hopkins S.P., Shearier E.R., Earley E.J., Gillette A.A., Aghion E., Bocks M., Drelich J.W., Goldman J. (2016). Corrosion characteristics dictate the long-term inflammatory profile of degradable zinc arterial implants. ACS Biomater. Sci. Eng..

[289] Bowen P.K., Drelich A., Drelich J., Goldman J. (2015). Rates of in vivo (arterial) and in vitro biocorrosion for pure magnesium. J. Biomed. Mater. Res..

[290] Atrens A., Liu M., Abidin N.I.Z., Song G.L., Song G.-l. (2011). Corrosion of magnesium (Mg) alloys and metallurgical influence. Corrosion of Magnesium Alloys.

[291] Kubásek J., Dvorský D., Čapek J., Pinc J., Vojtěch D. (2019). Zn-Mg biodegradable composite: novel material with tailored mechanical and corrosion properties. Materials.

[292] Yang H., Qu X., Lin W., Chen D., Zhu D., Dai K., Zheng Y. (2019). Enhanced osseointegration of Zn-Mg composites by tuning the release of Zn ions with sacrificial Mg-rich anode design. ACS Biomater. Sci. Eng..

[293] Pan C., Sun X., Xu G., Su Y., Liu D. (2020). The effects of β-TCP on mechanical properties, corrosion behavior and biocompatibility of β-TCP/Zn-Mg composites. Mater. Sci. Eng. C.

[294] Guoliang L., Guangquan X., Bobo H., Li L., Debao L. (2019). Fabrication and properties of a biodegradable β-TCP/Zn-Mg bio-composite. Mater. Res. Express.

[295] Shahin M., Munir K., Wen C., Li Y. (2019). Magnesium matrix nanocomposites for orthopedic applications: a review from mechanical, corrosion, and biological perspectives. Acta Biomater..

[296] Baino F., Novajra G., Miguez-Pacheco V., Boccaccini A.R., Vitale-Brovarone C. (2016). Bioactive glasses: special applications outside the skeletal system. J. Non-Cryst. Solids.

[297] Park J.B., Brook R.J. (1991). Aluminum oxide: biomedical applications. Concise Encyclopedia of Advanced Ceramic Materials.

[298] Aghajani Derazkola H., Simchi A. (2018). Effects of alumina nanoparticles on the microstructure, strength and wear resistance of poly(methyl methacrylate)-based nanocomposites prepared by friction stir processing. J. Mech. Behav. Biomed. Mater..

[299] Chen Y.-W., Moussi J., Drury J.L., Wataha J.C. (2016). Zirconia in biomedical applications. Expet Rev. Med. Dev..

[300] Chang Q., Chen D.L., Ru H.Q., Yue X.Y., Yu L., Zhang C.P. (2010). Toughening mechanisms in iron-containing hydroxyapatite/titanium composites. Biomaterials.

[301] Witte F., Feyerabend F., Maier P., Fischer J., Störmer M., Blawert C., Dietzel W., Hort N. (2007). Biodegradable magnesium–hydroxyapatite metal matrix composites. Biomaterials.

[302] Zhao J., Yu Z.M., Yu K., Chen L.J. (2011). Biodegradable behaviors of Mg-6% Zn-5% hydroxyapatite biomaterial. Adv. Mater. Res..

[303] Webster T.J., Ergun C., Doremus R.H., Bizios R. (2002). Hydroxylapatite with substituted magnesium, zinc, cadmium, and yttrium. II. Mechanisms of osteoblast adhesion. J. Biomed. Mater. Res..

[304] Cui Z., Zhang Y., Cheng Y., Gong D., Wang W. (2019). Microstructure, mechanical, corrosion properties and cytotoxicity of beta-calcium polyphosphate reinforced ZK61 magnesium alloy composite by spark plasma sintering. Mater. Sci. Eng. C.

[305] Pinc J., Čapek J., Hybášek V., Průša F., Hosová K., Maňák J., Vojtěch D. (2020). Characterization of newly developed zinc composite with the content of 8 wt.% of hydroxyapatite particles processed by extrusion. Materials.

[306] Guan Z., Linsley C.S., Hwang I., Yao G., Wu B.M., Li X. (2020). Novel zinc/tungsten carbide nanocomposite as bioabsorbable implant. Mater. Lett..

[307] Hwang I., Guan Z., Li X. (2018). Fabrication of zinc–tungsten carbide nanocomposite using cold compaction followed by melting. J. Manuf. Sci. Eng..

[308] Guan Z., Linsley C.S., Pan S., DeBenedetto C., Liu J., Wu B.M., Li X. (2020). Highly ductile Zn-2Fe-WC nanocomposite as biodegradable. Material, Metallurgical and Materials Transactions A.

[309] Guan Z., Yao G., Zeng Y., Li X. (2020). Fabrication and characterization of in situ Zn-TiB_2_ nanocomposite. Procedia Manufacturing.

[310] Pathak D.K., Pandey P.M. (2020). An experimental investigation of the fabrication of biodegradable zinc–hydroxyapatite composite material using microwave sintering. Pro. Inst. Mech. Eng. C: J. Mech. Eng. Sci..

[311] Pinc J., Miklášová E., Průša F., Čapek J., Drahokoupil J., Vojtěch D. (2019). Influence of processing on the microstructure and the mechanical properties of Zn/HA8 wt.% biodegradable composite. Manuf. Technol..

[312] Jan P., Jaroslav Č., Jiří K., Filip P., Vojtěch H., Petr V., Ivona S. (2020). Characterization of a Zn-Ca_5_(PO_4_)_3_(OH) composite with a high content of the hydroxyapatite particles prepared by the spark plasma sintering process. Metals.

[313] Rai A., Rai P., Kumar V., Singh N.K., Singh V.K. (2020). Effect of sintering temperature on the physico-mechanical behavior of SiC reinforced zinc-magnesium based composite. Met. Mater. Int..

[314] Yu M., George C., Cao Y., Wootton D., Zhou J. (2014). Microstructure, corrosion, and mechanical properties of compression-molded zinc-nanodiamond composites. J. Mater. Sci..

[315] Hongmei A., Shanshan P., Zongkui Z., Yuan L., Qianfei D., Yan H., Fei Z. (2020). Microstructure and mechanical properties of zinc matrix composites reinforced with copper coated multiwall carbon nanotubes. Mater. Res. Express.

[316] Rai A., Rai P., Kumar V., Singh N.K., Singh V.K. (2020). Study of mechanical, electrochemical, cellular and antibacterial response of Zn-2Mg-6SiC biodegradable implant. Ceram. Int..

[317] Munir K.S., Wen C., Li Y. (2019). Carbon nanotubes and graphene as nanoreinforcements in metallic biomaterials: a review. Adv. Biosys..

[318] Goh C.S., Wei J., Lee L.C., Gupta M. (2007). Properties and deformation behaviour of Mg–Y_2_O_3_ nanocomposites. Acta Mater..

[319] Kang Y.-C., Chan S.L.-I. (2004). Tensile properties of nanometric Al_2_O_3_ particulate-reinforced aluminum matrix composites. Mater. Chem. Phys..

[320] Munir K.S., Li Y., Lin J., Wen C. (2018). Interdependencies between graphitization of carbon nanotubes and strengthening mechanisms in titanium matrix composites. Materialia.

[321] Shahin M., Munir K., Wen C., Li Y. (2020). Magnesium-based composites reinforced with graphene nanoplatelets as biodegradable implant materials. J. Alloys Compd..

[322] Munir K., Wen C., Li Y. (2020). Graphene nanoplatelets-reinforced magnesium metal matrix nanocomposites with superior mechanical and corrosion performance for biomedical applications. J. Magnes. Alloy..

[323] Ahmad I., Islam M., Parvez S., AlHabis N., Umar A., Munir K.S., Wang N., Zhu Y. (2019). Reinforcing capability of multiwall carbon nanotubes in alumina ceramic hybrid nanocomposites containing zirconium oxide nanoparticles. Int. J. Refract. Metals Hard Mater..

[324] Yu Files, Arepalli Ruoff (2000). Tensile loading of ropes of single wall carbon nanotubes and their mechanical properties. Phys. Rev. Lett..

[325] Demczyk B.G., Wang Y.M., Cumings J., Hetman M., Han W., Zettl A., Ritchie R.O. (2002). Direct mechanical measurement of the tensile strength and elastic modulus of multiwalled carbon nanotubes. Mater. Sci. Eng., A.

[326] Yu M.-F., Lourie O., Dyer M.J., Moloni K., Kelly T.F., Ruoff R.S. (2000). Strength and breaking mechanism of multiwalled carbon nanotubes under tensile load. Science.

[327] Lee C., Wei X., Li Q., Carpick R., Kysar J.W., Hone J. (2009). Elastic and frictional properties of graphene. Phys. Status Solidi B.

[328] Lee C., Wei X., Kysar J.W., Hone J. (2008). Measurement of the elastic properties and intrinsic strength of monolayer graphene. Science.

[329] Munir K.S., Wen C. (2016). Deterioration of the strong sp2 carbon network in carbon nanotubes during the mechanical dispersion processing-a review. Crit. Rev. Solid State Mater. Sci..

[330] Parveen S., Kumar A., Husain S., Zulfequar M., Husain M. (2018). Synthesis of highly dense and vertically aligned array of SWCNTs using a catalyst barrier layer: high performance field emitters for devices. Phys. B Condens. Matter.

[331] Saito N., Usui Y., Aoki K., Narita N., Shimizu M., Hara K., Ogiwara N., Nakamura K., Ishigaki N., Kato H., Taruta S., Endo M. (2009). Carbon nanotubes: biomaterial applications. Chem. Soc. Rev..

[332] Boccaccini A.R., Chicatun F., Cho J., Bretcanu O., Roether J.A., Novak S., Chen Q.Z. (2007). Carbon nanotube coatings on bioglass‐based tissue engineering scaffolds. Adv. Funct. Mater..

[333] Eivazzadeh-Keihan R., Maleki A., de la Guardia M., Bani M.S., Chenab K.K., Pashazadeh-Panahi P., Baradaran B., Mokhtarzadeh A., Hamblin M.R. (2019). Carbon based nanomaterials for tissue engineering of bone: building new bone on small black scaffolds: a review. J. Adv. Res..

[334] Balandin A.A., Ghosh S., Bao W., Calizo I., Teweldebrhan D., Miao F., Lau C.N. (2008). Superior thermal conductivity of single-layer graphene. Nano Lett..

[335] Bolotin K.I., Sikes K.J., Jiang Z., Klima M., Fudenberg G., Hone J., Kim P., Stormer H.L. (2008). Ultrahigh electron mobility in suspended graphene. Solid State Commun..

[336] Dasari Shareena T., McShan D., Dasmahapatra A., Tchounwou P. (2018). A review on graphene-based nanomaterials in biomedical applications and risks in environment and health. Nano-Micro Lett..

[337] Rashad M., Pan F., Tang A., Asif M., She J., Gou J., Mao J., Hu H. (2015). Development of magnesium-graphene nanoplatelets composite. J. Compos. Mater..

[338] Dadkhah M., Saboori A., Fino P. (2019). An overview of the recent developments in metal matrix nanocomposites reinforced by graphene. Materials.

[339] Rashad M., Pan F., Asif M. (2016). Exploring mechanical behavior of Mg-6Zn alloy reinforced with graphene nanoplatelets. Mater. Sci. Eng., A.

[340] Rashad M., Pan F., Asif M., Chen X. (2017). Corrosion behavior of magnesium-graphene composites in sodium chloride solutions. J. Magnes. Alloy..

[341] Parizi M.T., Ebrahimi G.R., Ezatpour H.R., Paidar M. (2019). The structure effect of carbonaceous reinforcement on the microstructural characterization and mechanical behavior of AZ80 magnesium alloy. J. Alloys Compd..

[342] Huang S.J., Abbas A., Ballóková B. (2019). Effect of CNT on microstructure, dry sliding wear and compressive mechanical properties of AZ61 magnesium alloy. J. Mater. Res. Technol..

[343] Ghodrati H., Ghomashchi R. (2019). Effect of graphene dispersion and interfacial bonding on the mechanical properties of metal matrix composites: an overview. FlatChem.

[344] Handayani M., Ganta M., Darsono N., Sulistiyono E., Lestari F.P., Erryani A., Astawa I.N.G.P., Lusiana, S Azhari N. (2019). Multi-walled carbon nanotubes reinforced-based magnesium metal matrix composites prepared by powder metallurgy. IOP Conf. Ser. Mater. Sci. Eng..

